# Supplementary material for: Homo- and heterodehydrocoupling of phosphines mediated by alkali metal catalysts
Source: Nat Commun. 2019 Jun 26;10:2786. doi: 10.1038/s41467-019-09832-4 (PMC6594957; doi:10.1038/s41467-019-09832-4)
Supplement: Supplementary file 1 — Supplementary Information [file 41467_2019_9832_MOESM1_ESM.pdf]

Supplementary Information for

Homo- and Heterodehydrocoupling of Phosphines Mediated by Alkali Metal Catalysts

Wu and Annibale *et al.*

Supplementary Methods

All manipulations were carried out under an atmosphere of dry and deoxygenated N_2 in a glovebox (H_2O and O_2 < 0.1 ppm) or using standard Schlenk methods. Glassware was pre-dried in an oven at 250 °C for several hours and cooled under dynamic vacuum prior to use. Solvents were dried and deoxygenated using a Grubbs type column and stored over activated 3 Å molecular sieves for a minimum of 24 hours. Ph_2PH was obtained from Sigma-Aldrich, other substituted secondary phosphines and $PhPH_2$ were prepared according to the literature procedure.[1,2] *t*BuOK was obtained from Alfa Aesar and was sublimed prior to use. Imines were prepared either by condensation of amines with aldehydes, ketones or the hydrosilylation of benzonitrile. Azobenzene, hydrazobenzene, benzophenone and *trans*-stilbene were obtained from Sigma-Aldrich and used as received. Compounds **3a**,[3] and **7a**,[4] were independently prepared via literature procedures. The radical anion K[PhNNPh] was independently synthesised from potassium metal and azobenzene according to the literature.[5] NMR spectra were obtained using JEOL ECP 300 (300 MHz), Bruker-400 (400 MHz), or Varian NMRS500 (500 MHz) spectrometers at 298 K unless otherwise stated. Deuterated solvents were obtained from Sigma-Aldrich and dried by storage over activated 3 Å molecular sieves for 24 hours. Air sensitive samples were loaded and capped in NMR tubes inside the glovebox and sealed with Teflon tape and Parafilm prior to removal. ^{31}P NMR kinetic experiments were carried out in hermetically sealed J. Young valved NMR tubes with a capillary containing neat PCl_3 as an internal standard. Mass spectrometry experiments were carried out by the University of Bristol Mass Spectrometry Service on a Bruker Daltronics micro TOF II with a TOF analyser. Continuous-wave X-band electron paramagnetic resonance (EPR) spectra at 9 GHz were recorded on a Bruker Biospin EMX spectrometer with a Bruker ER4119HS resonator in quartz EPR tubes fitted with J. Young valves. The spectra were obtained with 2.2 mW microwave power and 0.1 G modulation amplitude under non-saturating conditions. Simulations of experimental EPR spectra were performed with EasySpin.[6] Elemental analysis was carried out on a CE Instruments (now Thermo) elemental analyser model EA1110 by Elemental Microanalysis Ltd.

General procedure for the *t*BuOK-catalysed homodehydrocoupling of phosphines

A J. Young NMR tube was charged with 0.1 mmol of the phosphines and 0.1 mmol of the corresponding hydrogen acceptors azobenzene (**HA-2**) or *N*-benzylideneaniline (**HA-5**), 0.5 mL

of 0.02 M *t*BuOK THF solution was added to the J. Young NMR tube. The NMR tube was sealed and heated at various temperature as indicated in the corresponding Fig. 2 and Fig. 4 or Supplementary Tables for the time shown and analysed by ^{31}P NMR spectroscopy. For isolated products, the solvents/volatiles were removed under vacuum and the residue was washed with hexanes, solid products were recrystallised through vapour diffusion of hexanes into THF solutions of the product.

General procedure for the *t*BuOK-catalysed heterodehydrocoupling of phosphines

A J. Young NMR tube was charged with 0.1 mmol of the phosphines and 0.1 mmol of azobenzene (**HA-2**) or *N*-benzylideneaniline (**HA-5**), 0.5 mL of 0.02 M *t*BuOK THF solution was added to the J. Young NMR tube, then 0.1 - 0.3 mmol of the corresponding alcohol, thiol, or amine was added. The NMR tube was sealed and heated for the time shown in the Fig. 5 and analysed by ^{31}P NMR spectroscopy. For isolated products, the reactions were performed in 0.5 mmol scale of phosphine and distilled under lower pressure for liquid products or purified *via* diffusion crystallization (THF/hexane).

General procedure for the *t*BuOK-catalysed homodehydrocoupling of phosphines in the presence of hydrazobenzene

A J. Young NMR tube was charged with 0.1 mmol of the phosphines and 0.1 mmol hydrazobenzene, 0.5 mL of 0.2 M *t*BuOK THF solution was added to the J. Young NMR tube. The NMR tube was sealed and analysed directly by ^{31}P NMR spectroscopy. The isolated products were obtained *via* diffusion crystallization (THF/hexane).

General procedure for the preparation of the secondary phosphines

Under N_2 atmosphere, a 250 mL flask was equipped with a gas inlet, refluxing condenser and addition funnel. A solution of the corresponding aryl halide (3.3 eq.) in 25 mL THF was slowly added to a suspension of 1.25 g (51.2 mmol, 3.3 eq.) magnesium turnings in 30 mL THF. After stirring at room temperature until no magnesium left, the refluxing condenser was removed, the mixture was cooled with an ice bath and a solution of 2.00 mL (15.5 mmol, 1.0 eq.) diethyl phosphite in 5 mL THF was added. The mixture was stirred for additional 15 minutes at low temperatures and two hours at room temperature. Subsequently 40 mL 0.1 N HCl was added dropwise over a period of 5 minutes at 0 °C, followed by the addition of 40 mL methyl *tert*-butyl

ether (MTBE) and stirring for further 5 minutes. The upper organic phase was decanted from the formed gel. To the remaining gel 20 mL of CH_2Cl_2 was added and the mixture agitated well for additional 5 minutes. The resultant mixture was then filtered through a frit equipped with celite. After washing the celite with CH_2Cl_2 (2 x 30mL) the organic phases were combined, dried over $MgSO_4$ and filter via a cannula to a second 250 mL flask and the solvent was removed *in vacuo* to obtain the corresponding phosphine oxide. Another 250 mL flask equipped with gas inlet and addition funnel was charged with a solution of the above phosphine oxide (1 eq.) in 30 mL THF. This solution was added over a period of 15 minutes to a 1 M solution of DIBAL-H in hexane (3 eq.) and stirred for 30 min at ambient temperature. To the flask 50 mL freshly degassed MTBE was added via the addition funnel slowly. After cooling the solution to 0 °C, 30 mL degassed 2N aq. NaOH was added via the addition funnel followed by 10 mL sat. aq. NaCl. The solution was stirred for additional 5 minutes and warmed to room temperature. Stirring was subsequently stopped and the layers allowed to separate. The organic layer was then transferred via cannula to a second 250 mL flask charged with $MgSO_4$. After stirring for 10 minutes the mixture was filtered via a cannula to a third 250 mL flask and the solvent was removed *in vacuo* to obtain the phosphines.

X-ray Crystallography

X-ray diffraction quality crystals of **2j** and **3e**·(THF) were obtained by vapour diffusion of hexanes into a THF solution of the compound. X-ray diffraction experiments for compounds **2j** and **3e**·(THF) were carried out at 100 K on a Bruker APEX II diffractometer using Mo Kα radiation (λ = 0.71073 A). The data collections were performed using a CCD area detector from a single crystal mounted on a glass fibre. Intensities were integrated[7] and absorption corrections based on equivalent reflections using SADABS[8] were applied. The structures were all solved using direct methods and structures were refined against all *F*2 using ShelXL2013[9] or Olex2[10]. All of the non-hydrogen atoms were refined anisotropically. Hydrogen atoms were calculated geometrically and refined using a riding model. Compound **2j** is a racemic twin and the absolute structure was not determined due to low Friedel pair coverage. The X-ray structure of $[CyP]_4$ (**2j**) was previously reported by Burford and coworkers at 153 K[11].

Supplementary Notes

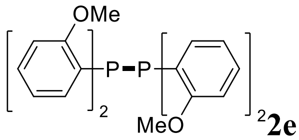
2e

White solid (45% isolated yield using **HA-2**): **^{1}H NMR** (400 MHz, Methylene Chloride-d_2) δ 7.46 (dd, J = 7.5, 1.7 Hz, 4H), 7.08 (ddd, J = 8.2, 7.3, 1.7 Hz, 4H), 6.74 (td, J = 7.4, 1.1 Hz, 4H), 6.57 – 6.39 (m, 4H), 3.41 (s, 12H). **^{13}C NMR** (101 MHz, Methylene Chloride-d_2) δ 160.9 (t, J = 9.3 Hz), 135.1 (t, J = 8.7 Hz), 129.3, 123.7, 120.1, 109.4, 55.0. **^{31}P NMR** (162 MHz, Methylene Chloride-d_2) δ -46.18. **ESI-MS**: 491.1524 m/z $[M+H]^+$ (calculated: 491.1535 m/z).

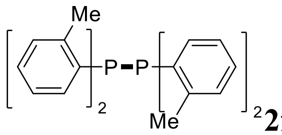
2f

White solid (73% isolated yield using **HA-5**): **^{1}H NMR** (400 MHz, THF-d_8) δ 7.40 (dd, J = 7.6, 1.4 Hz, 4H), 7.05 (td, J = 7.4, 1.4 Hz, 4H), 6.95 – 6.89 (m, 8H), 1.83 (s, 12H). **^{13}C NMR** (101 MHz, THF-d_8) δ 142.8 (t, J = 13.6 Hz), 134.9 (t, J = 7.1 Hz), 134.3 (t, J = 5.4 Hz), 129.6 (t, J = 2.5 Hz), 128.4, 125.4, 20.0. **^{31}P NMR** (162 MHz, THF-d_8) δ -37.16. **ESI-MS**: 427.1732 m/z $[M+H]^+$ (calculated: 427.1739 m/z). **Anal**. calcd for $C_{28}H_{28}P_2$ (%): C 78.86, H 6.62. Found C 78.89, H 5.97.

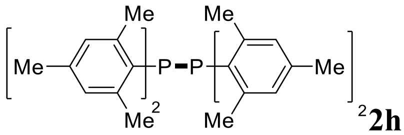
2h

White solid (55% isolated yield using **HA-2**): **^{1}H NMR** (400 MHz, Methylene Chloride-d_2) δ 6.57 (s, 4H), 2.09 (brs, 18H). **^{13}C NMR** (101 MHz, THF-d_8) δ 129.4, 22.1, 19.9. **^{31}P NMR** (162 MHz, Methylene Chloride-d_2) δ -30.30. **ESI-MS**: 539.2991 m/z $[M+H]^+$ (calculated: 539.2991 m/z).

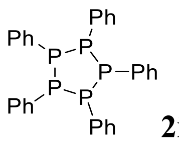
2i

White solid (60% isolated yield using **HA-5**): **^{1}H NMR** (400 MHz, Benzene-d_6) δ 7.84 (d, J = 7.4 Hz, 1H), 7.19 – 7.11 (m, 1H), 6.86-6.76 (m, 2H), 6.69 – 6.59 (m, 1H). **^{13}C NMR** (101 MHz, Benzene-d_6) δ 134.1-133.8 (m, C_{meta}), 132.7-132.5 (m, C_{ipso}), 128.7-128.5 (m, C_{ortho}), 128.4 (s, C_{para}). **^{31}P NMR** (162 MHz, Benzene-d_6) δ -1.3 – -5.6 (m). **ESI-MS**: 541.0715 m/z $[M+H]^+$ (calculated: 541.0717 m/z). **Anal**. calcd for $C_{30}H_{25}P_5$ (%): C 66.7, H 4.7. Found C 67.1, H 4.8.

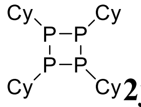
2j

White solid (60% isolated yield using **HA-2**): **^{1}H NMR** (400 MHz, Methylene Chloride-d_2) δ 1.86 (d, J = 12.9 Hz, 6H), 1.77 – 1.65 (m, 9H), 1.60 (brs, 3H), 1.46 (s, 6H), 1.21-1.12 (m, 11H), 1.04-0.99 (m, 7H), 0.84 – 0.74 (m, 2H). **^{13}C NMR** (101 MHz, Methylene Chloride-d_2) δ 38.7, 29.9 – 29.4 (m), 26.9, 26.3. **^{31}P NMR** (162 MHz, Methylene Chloride-d_2) δ -67.47. **ESI-MS**: 457.2467 m/z $[M+H]^+$ (calculated: 457.2466 m/z). For crystallographic data, see below.

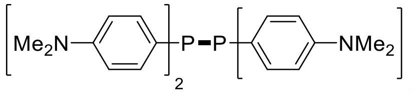
2l

White solid (81% isolated yield using hydrazobenzene): **^{1}H NMR** (500 MHz, Benzene-d_6) δ 7.82 (dt, J = 8.9, 2.7 Hz, 8H), 6.45 (d, J = 8.7 Hz, 8H), 2.36 (s, 24H). **^{13}C NMR** (126 MHz, Benzene-d_6) δ 150.4, 135.8 (t, J = 13.1 Hz), 123.4, 112.4 (t, J = 3.7 Hz), 39.5. **^{31}P NMR** (162 MHz, Benzene-d_6) δ -19.53.

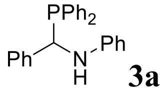
3a

White solid (95% isolated yield, 91% purity, from **HA-5** and **1a** using 10 mol% *t*BuOK in THF at 60 °C for 5 min, see Supplementary Figure 1): **^{1}H NMR** (400 MHz, THF-d_8) δ 7.42 – 7.35 (m, 2H), 7.23 – 7.16 (m, 3H), 7.11 – 7.06 (m, 1H), 7.02 – 6.98 (m, 4H), 6.96 – 6.90 (m, 5H), 6.85 (dd, J = 8.6, 7.3 Hz, 2H), 6.49 (d, J = 7.6 Hz, 2H), 6.39 (tt, J = 7.3, 1.1 Hz, 1H), 5.44 (dd, J = 7.6, 4.0 Hz, 1H), 5.05 (dd, J = 7.6, 4.6 Hz, 1H). **^{13}C NMR** (101 MHz, THF-d_8) δ 147.7 (d, J = 10.0

Hz), 140.6 (d, J = 12.0 Hz), 136.8 (d, J = 17.9 Hz), 136.3 (d, J = 13.4 Hz), 134.2 (d, J = 19.4 Hz), 133.8 (d, J = 17.0 Hz), 132.7 (d, J = 17.2 Hz), 128.6 (t, J = 15.6 Hz), 128.2 (d, J = 5.9 Hz), 127.8 (d, J = 2.3 Hz), 127.7, 127.6 (d, J = 1.4 Hz), 116.8, 113.4, 57.7 (d, J = 9.5 Hz). **^{31}P NMR** (162 MHz, THF-d_8) δ 3.27. **ESI-MS**: 390.1380 m/z $[M+Na]^+$ (calculated: 390.1382 m/z). **Anal**. calcd for $C_{25}H_{22}NP$ (%): C 81.72, H 6.04, N 3.81. Found C 81.55, H 5.81, N 3.51.

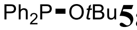

5a

Colourless liquid (88% isolated yield using **HA-2**): **^{1}H NMR** (500 MHz, THF-d_8) δ 7.48 – 7.44 (m, 4H), 7.31 – 7.21 (m, 6H), 1.39 (s, 9H). **^{13}C NMR** (126 MHz, THF-d_8) δ 144.0 (d, J = 17.1 Hz), 129.7 (d, J = 22.6 Hz), 128.3, 127.8 (d, J = 6.8 Hz), 76.1 (d, J = 13.4 Hz), 29.3 (d, J = 9.0 Hz). **^{31}P NMR** (202 MHz, THF-d_8) δ 86.88. **ESI-MS**: 281.1057 m/z $[M+Na]^+$ (calculated: 281.1066 m/z). **Anal**. calcd for $C_{16}H_{19}OP$ (%): C 74.40, H 7.41. Found C 73.95, H 7.54.

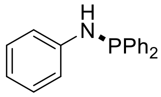

6a

White solid (80% isolated yield using **HA-2**): **^{1}H NMR** (400 MHz, THF-d_8) δ 7.32 (td, J = 7.7, 1.6 Hz, 4H), 7.22 – 7.13 (m, 5H), 7.00 – 6.91 (m, 4H), 6.59 – 6.52 (m, 1H), 5.89 (d, J = 8.6 Hz, 1H). **^{13}C NMR** (101 MHz, THF-d_8) δ 147.67 (d, J = 18.3 Hz), 140.91 (d, J = 12.7 Hz), 131.05 (d, J = 20.5 Hz), 128.65 (d, J = 1.4 Hz), 128.44, 128.07 (d, J = 6.5 Hz), 118.40 (d, J = 1.5 Hz), 115.75 (d, J = 13.9 Hz). **^{31}P NMR** (162 MHz, THF-d_8) δ 26.22. **ESI-MS**: 278.1099 m/z $[M+H]^+$ (calculated: 278.1093 m/z).

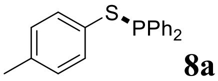

8a

White solid (85% isolated yield using **HA-2**): **^{1}H NMR** (400 MHz, THF-d_8) δ 7.48 – 7.40 (m, 4H), 7.23 – 6.92 (m, 8H), 6.93 (d, J = 7.9 Hz, 2H), 2.15 (s, 3H). **^{13}C NMR** (101 MHz, THF-d_8) δ 137.9 (d, J = 24.5 Hz), 136.9 (d, J = 1.9 Hz), 132.5 (d, J = 21.1 Hz), 132.1 (d, J = 7.0 Hz), 129.5, 129.0, 128.3 (d, J = 6.3 Hz), 20.1. **^{31}P NMR** (162 MHz, THF-d_8) δ 33.73. **ESI-MS**: 309.0864 m/z $[M+H]^+$ (calculated: 309.0861 m/z). **Anal**. calcd for $C_{19}H_{17}PS$ (%): C 74.00, H 5.56, Found C 73.70, H 5.79.

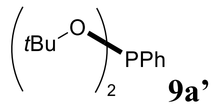

9a'

Colourless liquid (40% isolated yield using hydroazobenzene): **^{1}H NMR** (400 MHz, THF-d_8) δ 7.49 – 7.45 (m, 2H), 7.26 – 7.17 (m, 3H), 1.29 (d, J = 0.5 Hz, 18H). **^{13}C NMR** (101 MHz, THF-d_8) δ 146.3 (d, J = 13.8 Hz), 129.2 (d, J = 23.7 Hz), 128.7, 127.5 (d, J = 6.2 Hz), 76.2 (d, J = 10.1 Hz), 30.3 (d, J = 8.4 Hz). **^{31}P NMR** (162 MHz, THF-d_8) δ 139.07. **Anal**. calcd for $C_{14}H_{23}O_2P$ (%): C 66.12, H 9.12. Found C 66.10, H 9.56.

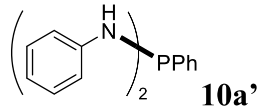

10a'

White solid (44% isolated yield using **HA-2**): **^{1}H NMR** (400 MHz, Methylene Chloride-d_2) δ 7.61 – 7.57 (m, 2H), 7.44 – 7.28 (m, 3H), 7.16 – 7.04 (m, 4H), 6.91 – 6.88 (m, 4H), 6.73 (m, 2H), 4.83 (s, 2H). **^{13}C NMR** (101 MHz, Methylene Chloride-d_2) δ 145.4 (d, J = 13.2 Hz), 141.1 (d, J = 3.3 Hz), 129.8 (d, J = 17.6 Hz), 129.2, 129.1, 128.7 (d, J = 4.7 Hz), 119.9 (d, J = 1.5 Hz), 116.4 (d, J = 11.7 Hz). **^{31}P NMR** (162 MHz, Methylene Chloride-d_2) δ 45.8. **ESI-MS**: 293.1210 m/z $[M+H]^+$ (calculated: 293.1202 m/z) **Anal**. calcd for $C_{18}H_{17}N_2P$ (%): C 73.96, H 5.86, N 9.58 Found C 73.93, H 5.88, N 9.55.

Supplementary Table 1. Initial screening of different H_2-Acceptors in the homodehydrocoupling of Ph_2PH[a]

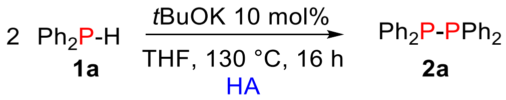

Entries	H_2-Acceptors	Conversion [%][b,c]	Yield [%][b]
		**1a**	**2a**
1	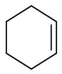	0	0
2[c]	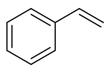	>99	0
3[c]	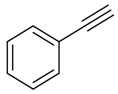	>99	0
4	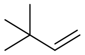	0	0
5	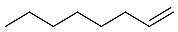	0	0
6	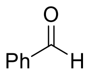	3	0
7	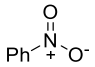	>99	0
8	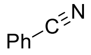	0	0

[a]Reactions were performed with 0.1 mmol Ph_2PH, 0.01 mmol *t*BuOK, 0.1 mmol different hydrogen acceptors, 0.5 mL THF in a J. Young NMR tube, 130 °C, 16 h; [b]Conversion and yields were determined by $^{31}P\{^1H\}$ NMR spectroscopy using a capillary of PCl_3 as a calibration standard. [c]Full conversion due to the hydrophosphination products.

Supplementary Table 2. Temperature and *t*BuOK loading effects in the homodehydrocoupling of Ph_2PH[a]

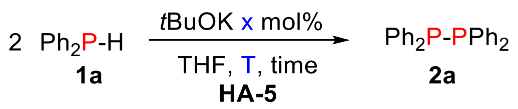

Entries	x [mol%]	T [°C]	Time [h]	Conversion [%][b] **1a**	Yield [%][b] **2a**
1	10	130	1	100	92
2	10	100	2.5	100	92
**3**	**10**	**60**	**8**	**100**	**95**
4	10	25	64	100	82
5	5	60	24	100	98
6	2.5	60	24	100	85

[a]Reactions were performed with 0.1 mmol Ph_2PH, 0.01 mmol *t*BuOK, 0.1 mmol of **HA-5**, 0.5 mL THF in a J. Young NMR tube, 130 °C. [b]Yields were determined by $^{31}P\{^1H\}$ NMR spectroscopy using a capillary of PCl_3 as a calibration standard.

Supplementary Table 3. Different bases in the homodehydrocoupling of Ph_2PH with **HA-5**[a]

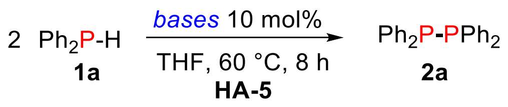

Entries	Bases	Conversion [%][b]	Yield [%][b]
		**1a**	**2a**
1	KOH	55	0
2	K_2CO_3	0	0
3	K_3PO_4	50	0
4	$NaHCO_3$	0	0
5	Et_3N	0	0
6	DABCO	0	0
7	*n*BuLi	73	0
8	NaOMe	61	0
9	NaH	100	37

[a]Reactions were performed with 0.1 mmol Ph_2PH, 0.01 mmol base, 0.1 mmol *N*-benzylideneaniline, 0.5 mL THF in a J. Young NMR tube, 60 °C, 8 h. [b]Conversion and yields were determined by $^{31}P\{^1H\}$ NMR spectroscopy using a capillary of PCl_3 as a calibration standard.

Supplementary Table 4. Effect of different *t*BuOM in the catalytic homodehydrocoupling of Ph_2PH^a

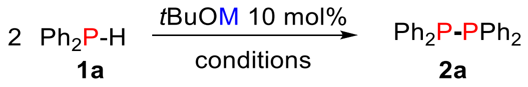

Entries	*t*BuOM	HA	T [°C]	Time [h]	Conversion [%][b] **1a**	Yield [%][b] **2a**
1	*t*BuOLi	HA-5	130	16	80	8
2		HA-2	25	1/60	100	55
3	*t*BuONa	HA-5	130	36	100	93
4		HA-2	25	1/60	100	59
5	*t*BuOK	HA-5	60	8	100	95
6		HA-2	25	1/60	100	75

[a]Reactions were performed with 0.1 mmol Ph_2PH, 0.01 mmol *t*BuOM, 0.1 mmol **HA** with 0.5 mL THF in a J. Young NMR tube. [b]Conversion and yields were determined by $^{31}P\{^1H\}$ NMR spectroscopy using a capillary of PCl_3 as a calibration standard. The differences in activity observed between the *t*BuOM catalysts might because of Lewis-acid co-activation effects and solubility differences.

Supplementary Table 5. Different imines in the *t*BuOK-catalysed homodehydrocoupling of Ph_2PH^a

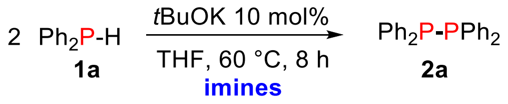

Entries	imines	Time [h]	Conversion [%][b]	Yield [%][b]
			**1a**	**2a**
1	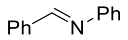	8	100	95
2	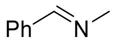	16	75	3
3	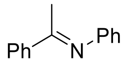	8	55	24
3	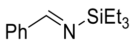	16	100	29
4	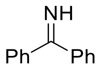	16	100	26
5	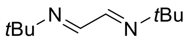	8	100	94

[a]Reactions were performed with 0.1 mmol Ph_2PH, 0.01 mmol *t*BuOK, 0.1 mmol different imines, 0.5 mL THF in a J. Young NMR tube, 60 °C, 8 h. [b]Conversion and yields were determined by ^{31}P NMR spectroscopy using a capillary of PCl_3 as a calibration standard.

Supplementary Table 6. Temperature and catalyst loading effects in the homodehydrocoupling of Ph_2PH in the presence of **HA-2**[a]

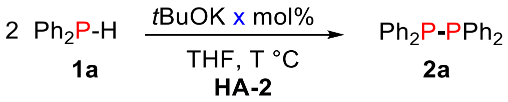

Entries	X [mol%]	T [°C]	Time [min]	Conversion [%][b] **1a**	Yield [%][b] **2a**
1	10	25	<5	100	75
2	5	25	<5	100	73
3	2.5	25	<5	100	59
4	1.0	25	<5	100	47
5	0.5	25	<5	73	39
6	0.1	25	<5	6	2
7	20	25	<5	100	68
8	10	-198 to 25[c]	<5	81	80
9	0	25	960	0	0
10	0	100	960	0	0
11	0	130	960	94	33

[a]Reactions were performed with 0.1 mmol Ph_2PH, 0.1x mmol *t*BuOK, 0.1 mmol **HA-2** with 0.5 mL THF in a J. Young NMR tube. [b]Conversion and yields were determined by $^{31}P\{^1H\}$ NMR spectroscopy using a capillary of PCl_3 as a calibration standard. [c]Reaction was run by mixing all the reagent together at -198 °C and measuring the $^{31}P\{^1H\}$ NMR spectrum while the reaction was warmed up to 25 °C.

Supplementary Table 7. Selected X-ray crystallographic information

**Compound**	**2j**	**3e**·(THF)
**Empirical formula**	$C_{24}H_{44}P_4$	$C_{31}H_{34}NO_3P$
**Formula weight**	456.47	499.56
**Temperature/K**	100(2)	100(2)
**Crystal system**	tetragonal	triclinic
**Space group**	$P\bar{4}2_1c$	$P\bar{1}$
**a/Å**	10.1045(9)	10.248(3)
**b/Å**	10.1045(9)	12.571(3)
**c/Å**	12.3267(12)	12.980(3)
**α/°**	90	61.779(15)
**β/°**	90	67.301(15)
**γ/°**	90	89.197(17)
**Volume/Å^3**	1258.6(3)	1329.9(6)
**Z**	2	2
**ρ_{calc}g/cm^3**	1.205	1.248
**μ/mm^{-1}**	0.309	0.136
**F(000)**	496.0	532.0
**Crystal size/mm^3**	0.2 × 0.2 × 0.2	0.48 × 0.22 × 0.15
**Radiation**	MoKα (λ = 0.71073)	MoKα (λ = 0.71073)
**2Θ range for data collection/°**	5.212 to 55.14	3.83 to 55.118
**Reflections collected**	11909	18859
**Independent reflections**	1466	6077
**Data/restraints/parameters**	1466/0/64	6077/0/331
**Goodness-of-fit on F^2**	1.079	1.013
**Final R indexes [I>=2σ (I)]**	R_1 = 0.0242, wR_2 = 0.0601	R_1 = 0.0363, wR_2 = 0.0810
**Final R indexes [all data]**	R_1 = 0.0263, wR_2 = 0.0609	R_1 = 0.0497, wR_2 = 0.0868
**Largest diff. peak/hole / e Å^{-3}**	0.28/-0.21	0.40/-0.24
**CCDC #**	1842531	1842532

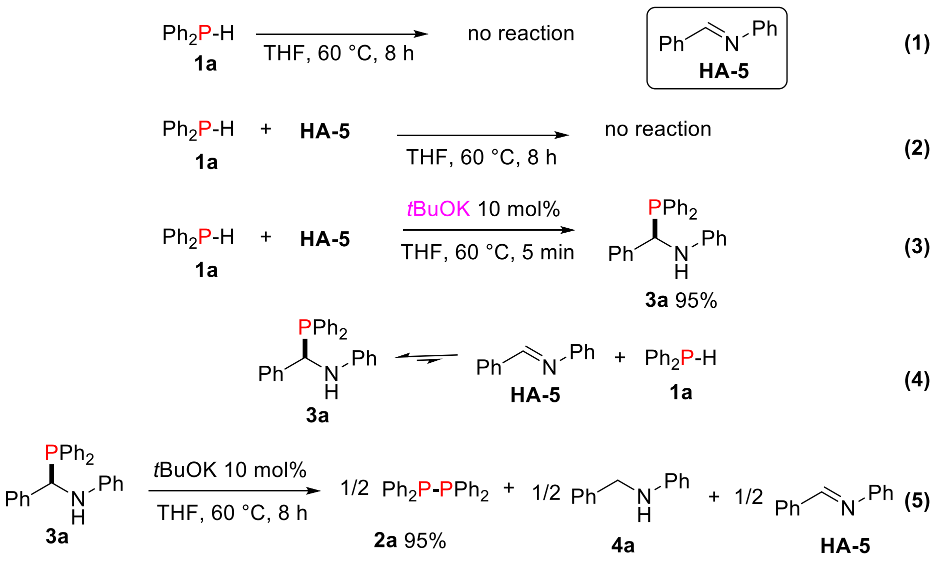

Supplementary Figure 1. Control experiments to rationalise the reaction mechanism: reactions were performed with 0.1 mmol scale of **1a** or **3a**, 0.5 mL THF in a J. Young NMR tube, 60 °C, and the rest of the conditions are shown in the equations. **3a** was also independently synthesised following a literature procedure.[3] The formation of **2a** in equation 5 presumably results from the reaction of **3a** with the $[PPh_2]^-$ anion formed from **1a** (generated by equilibration) and *t*BuOK.[12] When pentane washed samples of **3a** were dissolved in THF-d_8 or $CDCl_3$ equilibrium mixtures resulted with approximately 10% conversion of **3a** back to **HA-5** and **1a** at 25 °C.

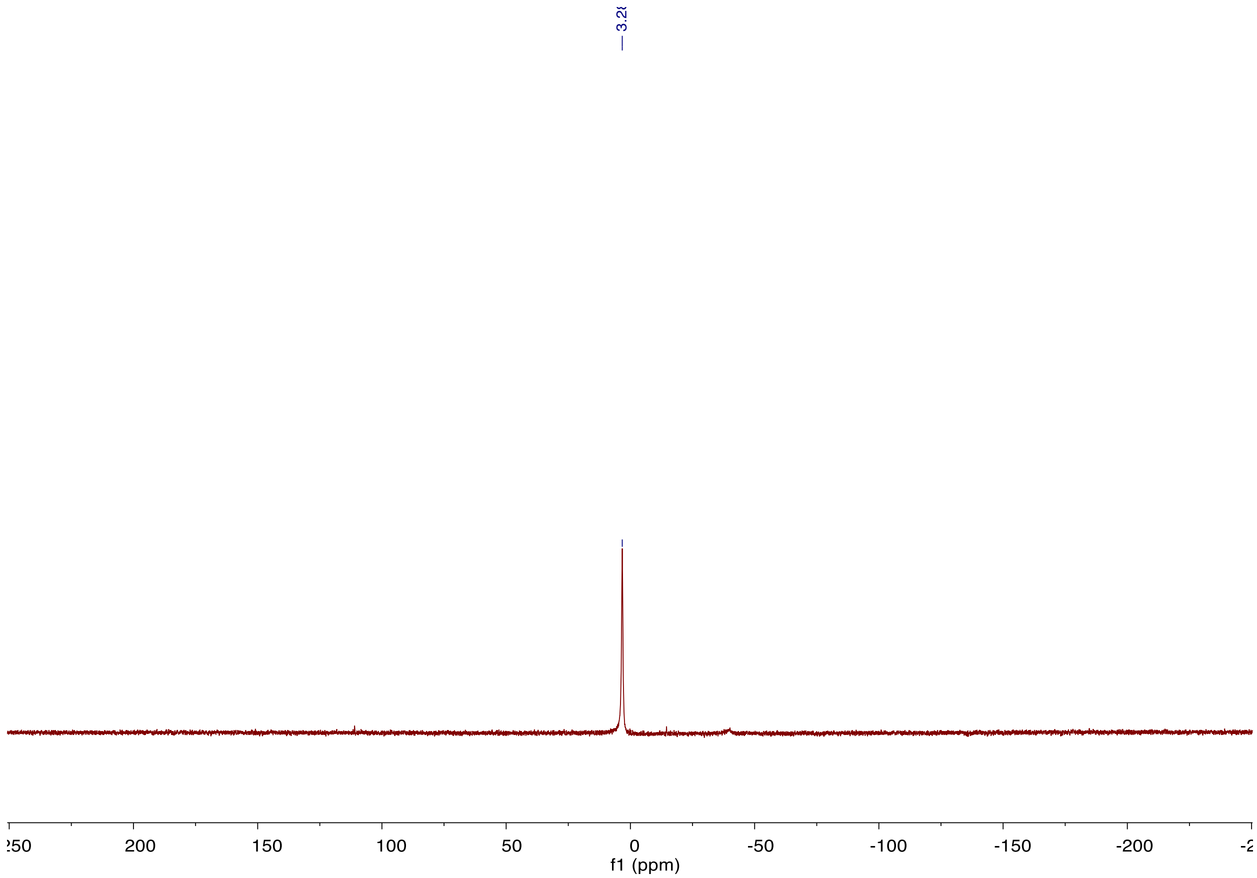

Supplementary Figure 2. $^{31}P\{^{1}H\}$ NMR spectrum (THF) after 10 mol% of *t*BuOK was added to the reaction solution containing **1a** and **HA-5**.

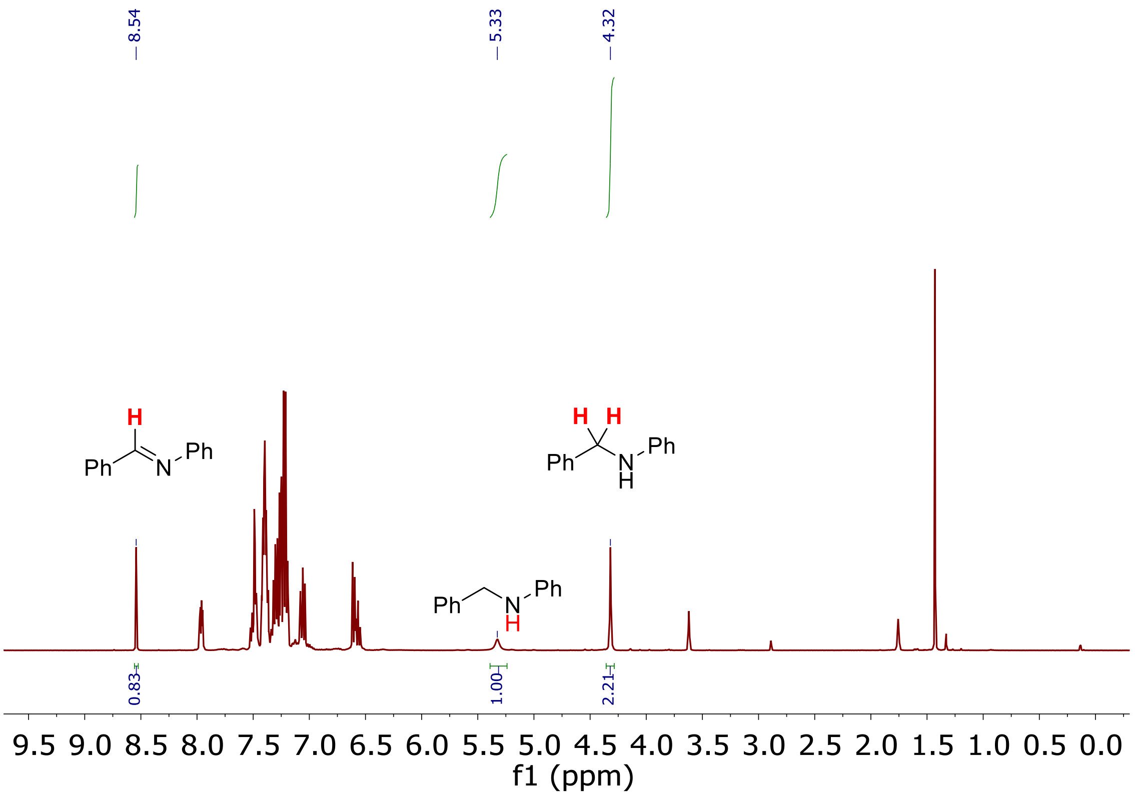

Supplementary Figure 3. ^{1}H NMR spectrum (400MHz, THF-d_8) of the reaction mixture after *t*BuOK-catalysed reaction of **3a** to **2a** and equal amounts of **HA-5** and **4a**.

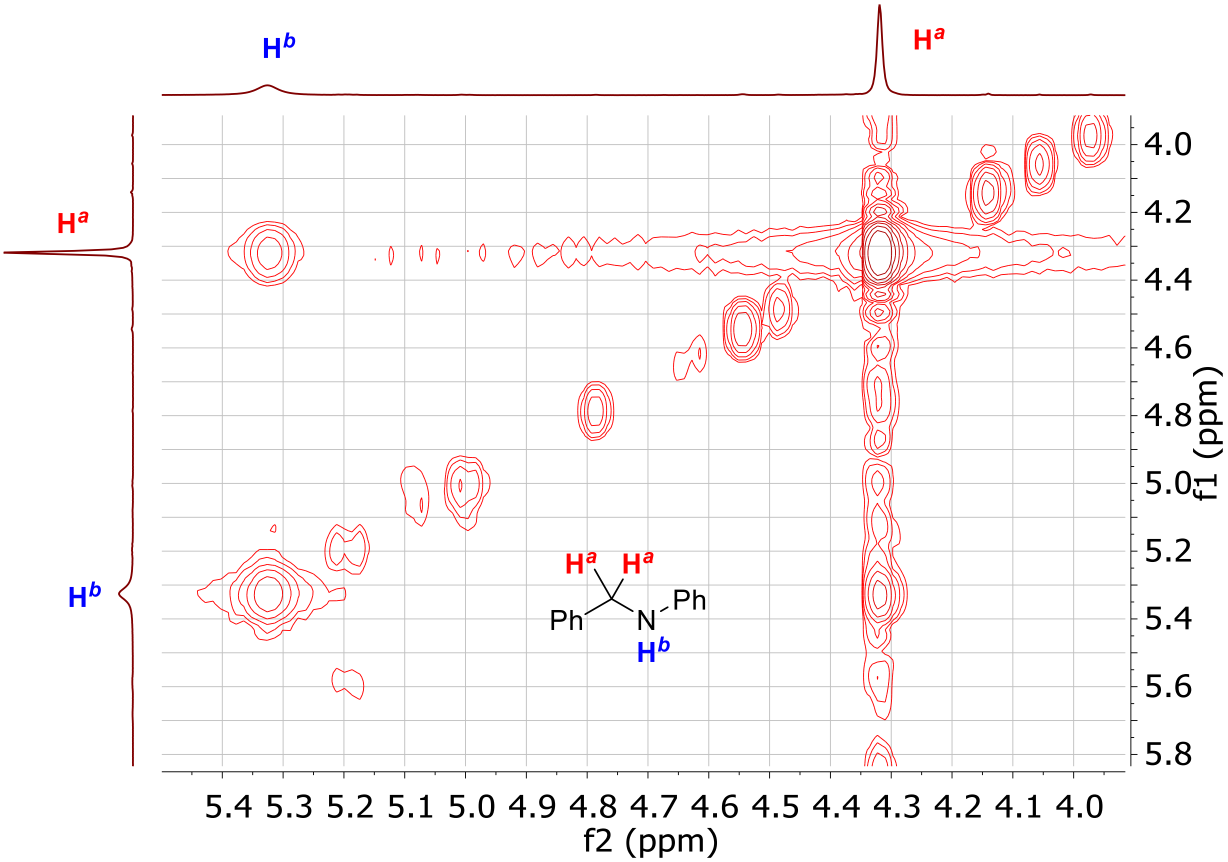

Supplementary Figure 4. ^1H-^1H COSY NMR spectrum of the reaction mixture after *t*BuOK-catalysed reaction of **3a** to a mixture of **2a, HA-5**, and **4a** in THF-d_8. Here the cross-peak allows assignment of benzylic CH_2 resonances for compound **4a** in the ^1H and ^{13}C NMR spectra.

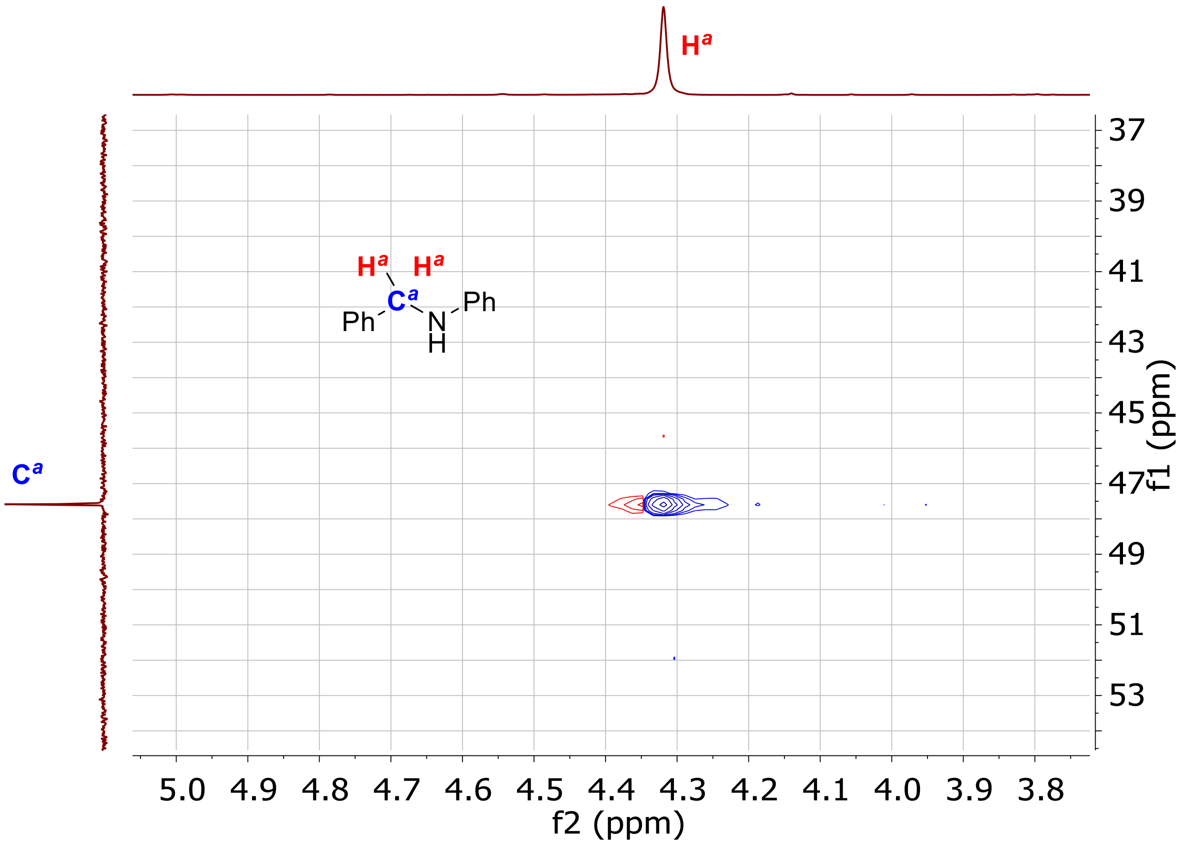

Supplementary Figure 5. ^{1}H-^{13}C HSQC NMR spectrum of the *t*BuOK-catalysed reaction of **3a** to **2a**, after reaction from the spectrum it is clearly demonstrating the structure of **4a**.

a)

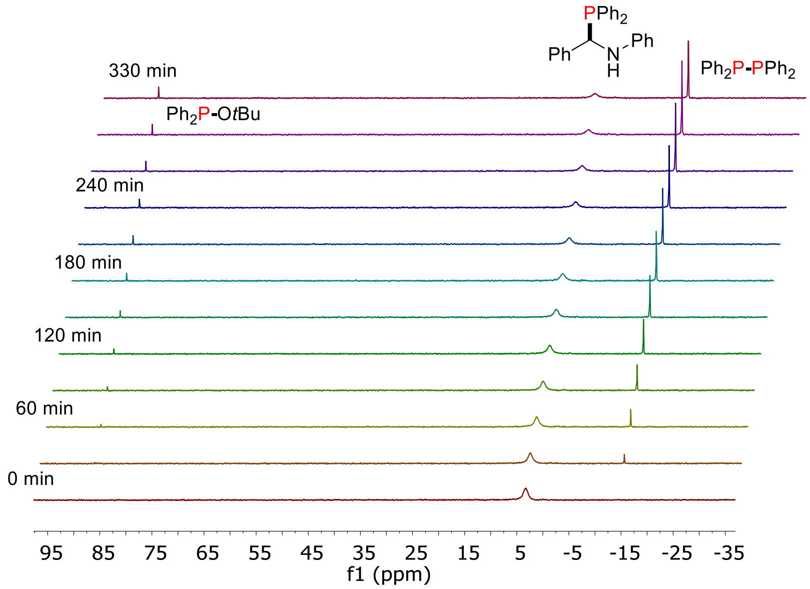

b)

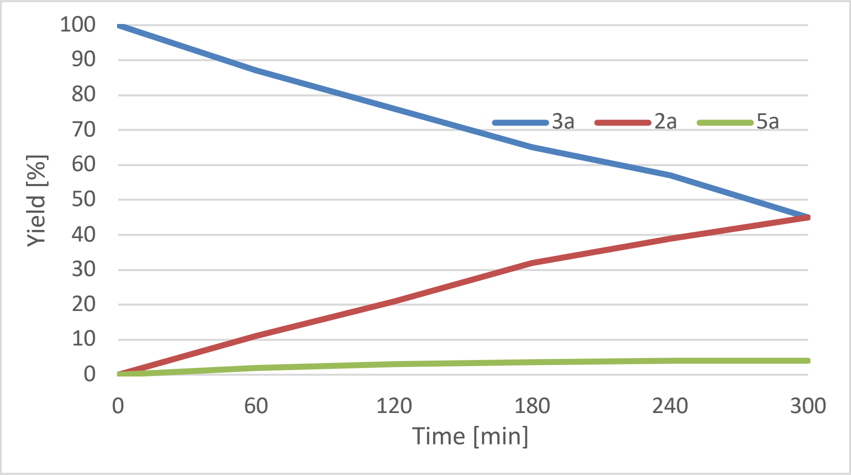

Supplementary Figure 6. Monitoring the *t*BuOK-catalysed homodehydrocoupling of Ph_2PH in the presence of **HA-5** at 30 min interval for the first 6.5 h by $^{31}P\{^1H\}$ NMR spectroscopy. a) Stacked $^{31}P\{^1H\}$ NMR spectra (THF); b) conversion vs time plot of different species.

Reaction was performed with 0.1 mmol Ph_2PH, 0.01 mmol *t*BuOK, 0.1 mmol **HA-5** and 0.5 mL THF in a J. Young NMR tube and heated at 50 °C in the NMR spectrometer (temperature was set to 15 °C lower than the boiling point of THF) and the instrument was programmed to automatically collect data over 16 h at 30 min intervals. Supplementary Figure 6 shows that during the reaction, there were no other *P*-containing species involved as reaction intermediates apart from the adduct **3a**, and minor amounts of compound **5a** by-product are also observed.

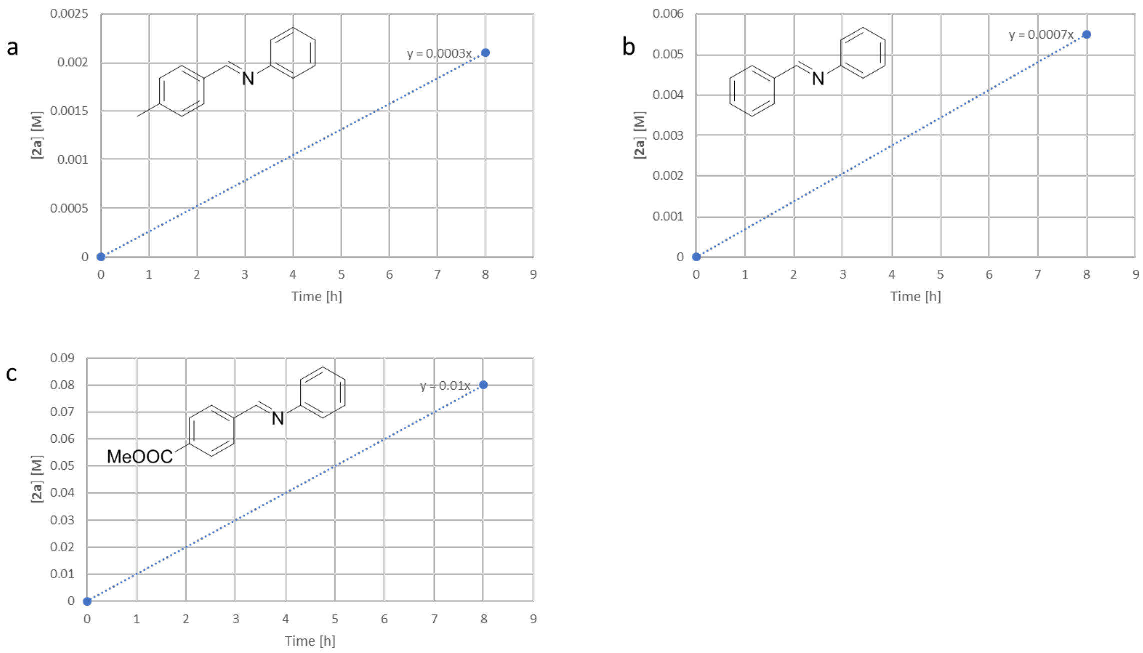

Supplementary Figure 7. Comparison of initial reaction rate with different *para*-substitution on the phenyl group of the imine at 25 °C.

Reactions were conducted with 0.1 mmol Ph_2PH, 0.01 mmol *t*BuOK, 0.1 mmol different imines with 0.5 mL THF in a J. Young NMR tube, the reactions were stopped after 8 h at 25 °C and the initial reaction rates were calculated based on the concentration of **2a**. Supplementary Figure 7 shows that with electron-withdrawing group on the *para*-position of the imine reacts faster and with electron-donating group reacts slower than the parent **HA-5** (reaction rate c>b>a) and this result support the formation of **4a**$^-$.

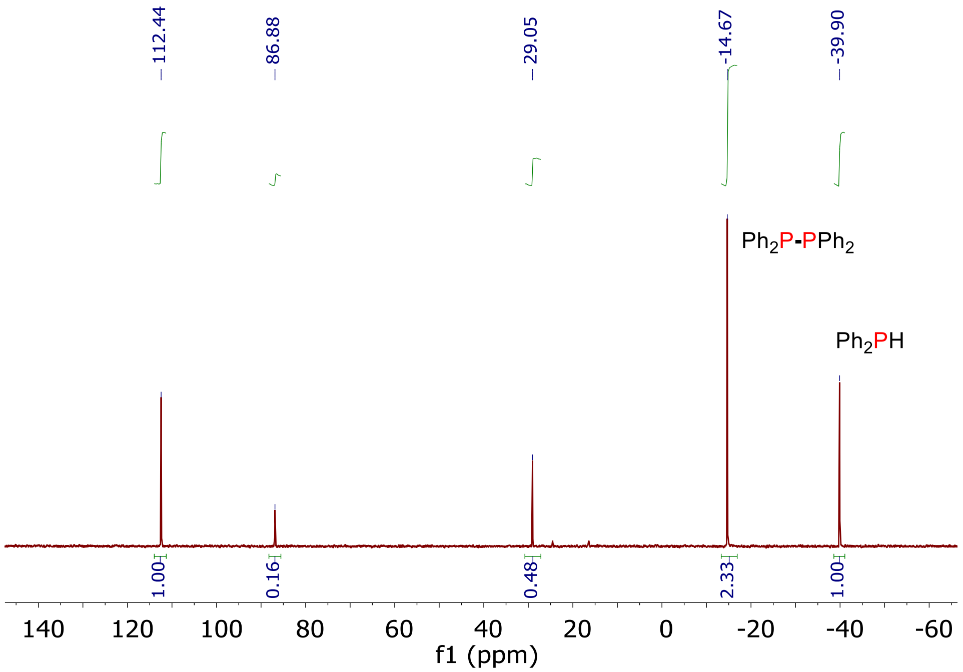

Supplementary Figure 8. $^{31}P\{^{1}H\}$ NMR spectrum (THF) from the reaction of 1 eq. Ph_2PH and 0.1 eq. *t*BuOK with 1 eq. of **HA-1** after heating at 130 °C in THF for 16 h, shows the presence of **2a** and other products.

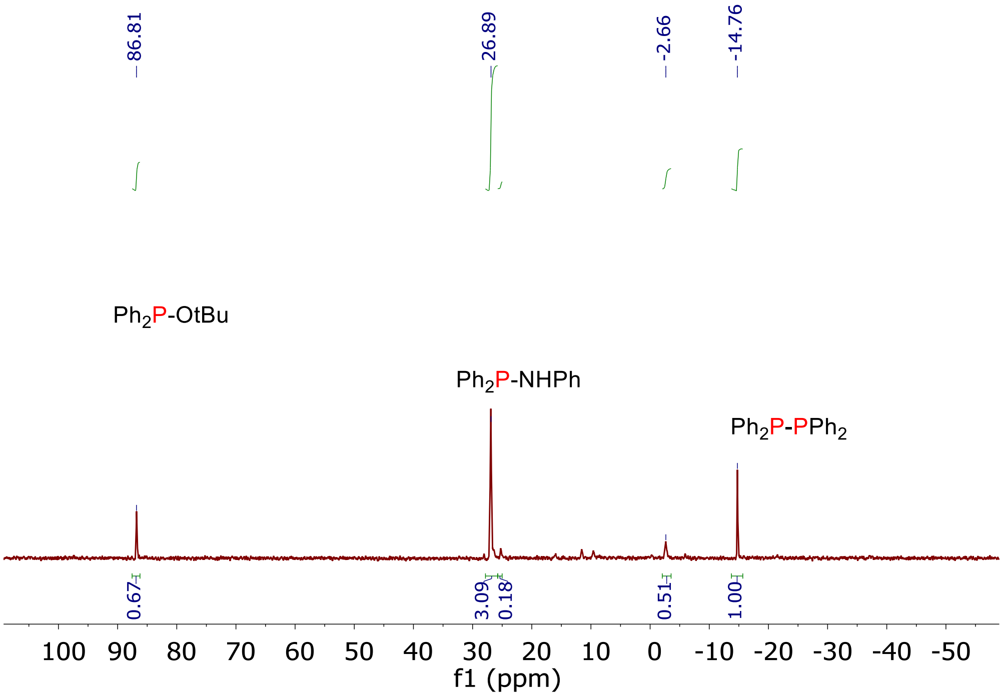

Supplementary Figure 9. $^{31}P\{^1H\}$ NMR spectrum (THF) from the reaction of 1 eq. Ph_2PH and 0.1 eq. *t*BuOK with 1 eq. of **HA-2** after heating at 130 °C in THF for 16 h, shows the presence of **2a** and other products assigned as Ph_2P-O*t*Bu (86.88 ppm) and Ph_2P-NHPh (26.89 ppm).

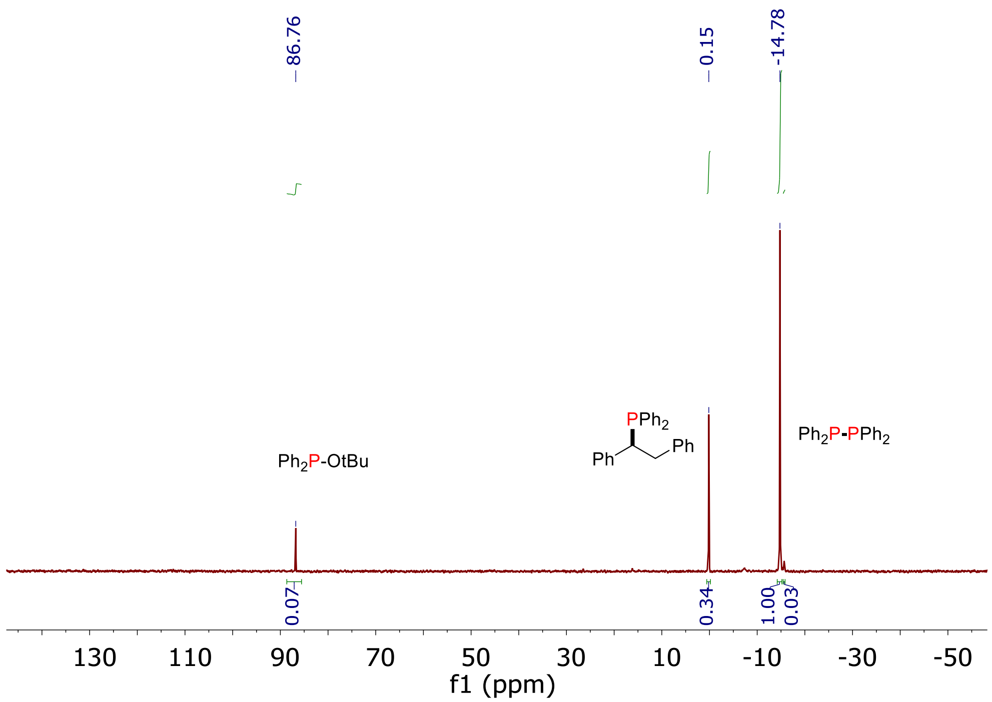

Supplementary Figure 10. $^{31}P\{^{1}H\}$ NMR spectrum (THF) from the reaction of 1 eq. Ph_2PH and 0.1 eq. *t*BuOK with 1 eq. of **HA-3** after heating at 130 °C in THF for 16 h, shows the presence of **2a** and hydrophosphination product, and Ph_2P-O*t*Bu.

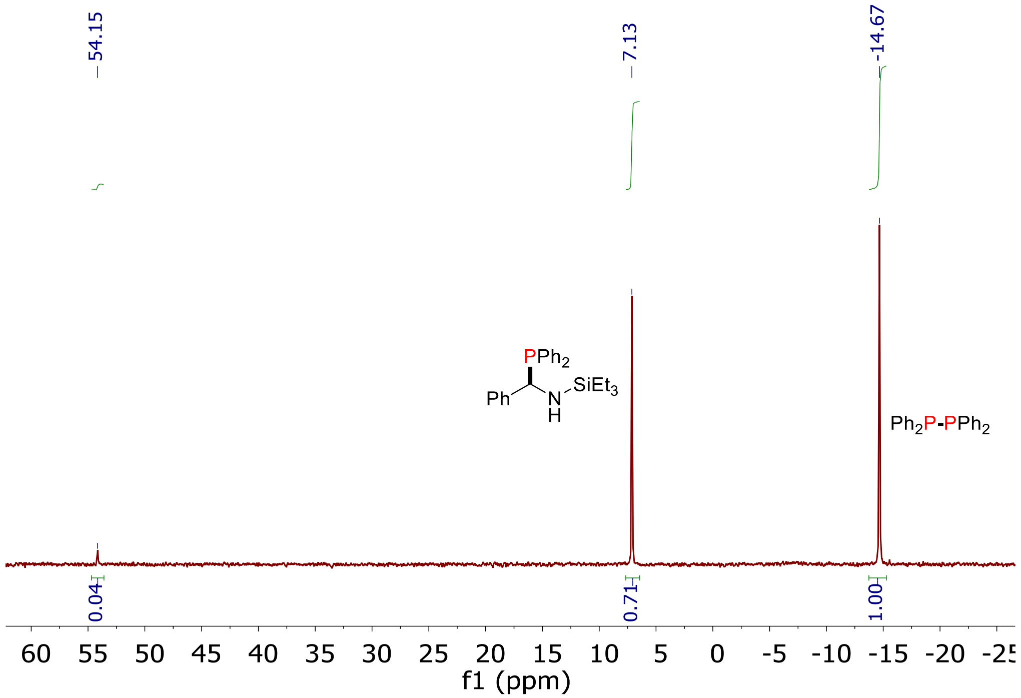

Supplementary Figure 11. $^{31}P\{^{1}H\}$ NMR spectrum (THF) from the reaction of 1 eq. Ph_2PH and 0.1 eq. *t*BuOK with 1 eq. of added **HA-4** after heating at 130 °C in THF for 16 h, shows the presence of **2a** and other products.

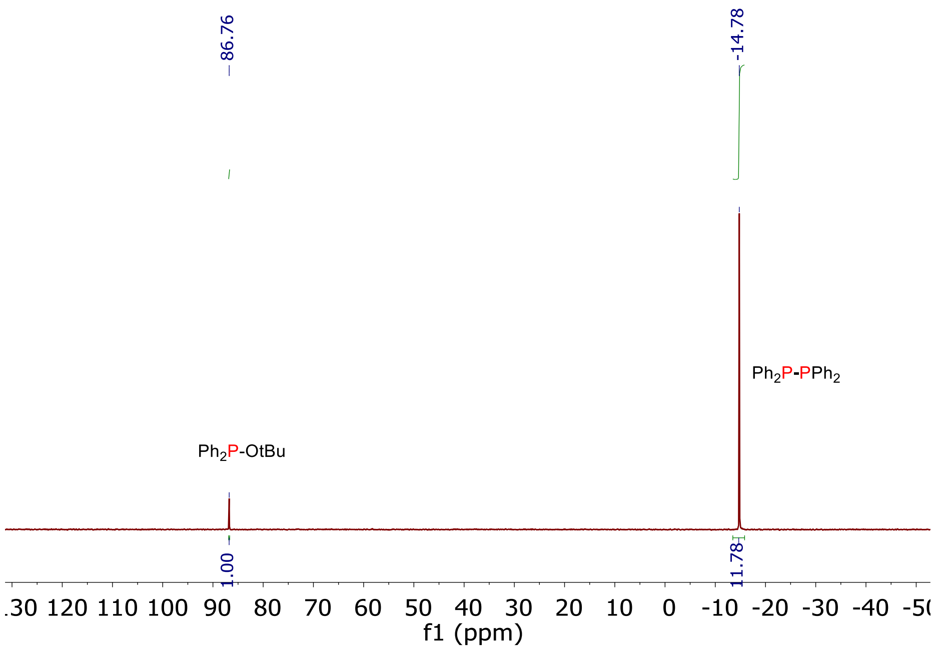

Supplementary Figure 12. $^{31}P\{^{1}H\}$ NMR spectrum (THF) from the reaction of 1 eq. Ph_2PH and 0.1 eq. *t*BuOK with 1 eq. of added **HA-5** after heating at 130 °C in THF for 16 h, shows the presence of **2a** and Ph_2P-O*t*Bu (86.88 ppm).

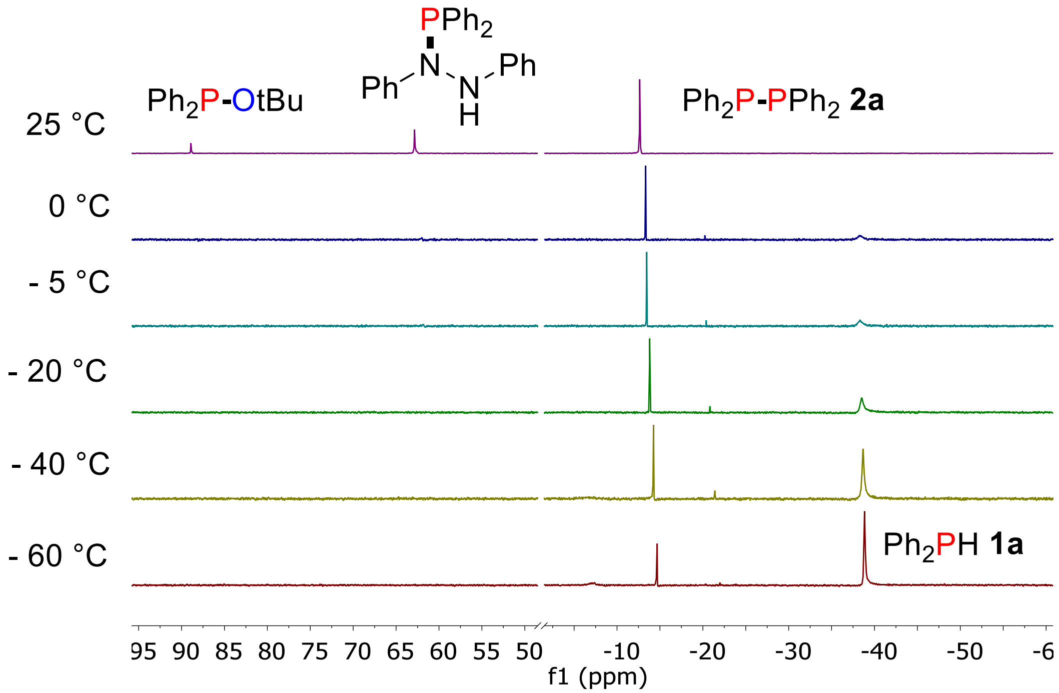

Supplementary Figure 13. Low-temperature $^{31}P\{^{1}H\}$ NMR spectra of *t*BuOK-catalysed homodehydrocoupling of **1a** in the presence of **HA-2** initially mixed at -60 °C and gradually warmed to 25 °C. Reaction was performed with 0.1 mmol of the phosphine, 0.01 mmol *t*BuOK, 0.1 mmol **HA-2**, in 0.5 mL THF in a J. Young NMR tube.

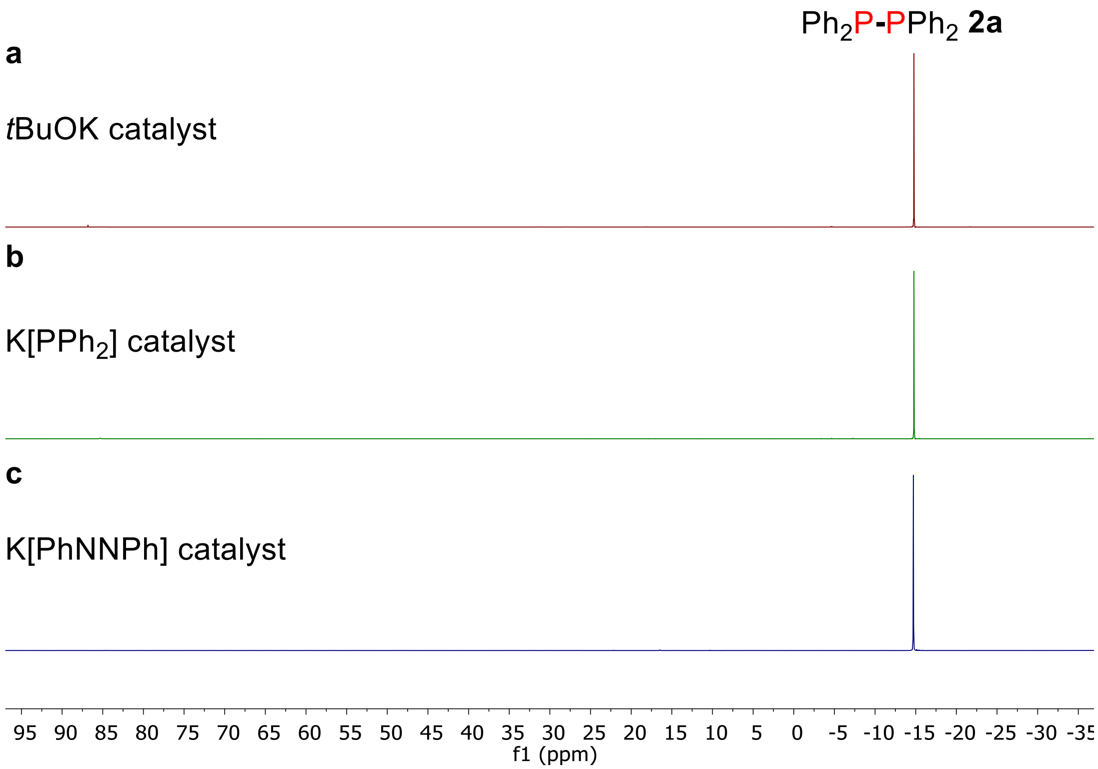

Supplementary Figure 14. Stacked $^{31}P\{^1H\}$ NMR spectra showing the formation of **2a** after the reaction of 0.22 mmol of **1a** and 0.1 mmol of **HA-2** in THF-d_8 at 25 °C with either **a)** 0.01 mmol of *t*BuOK, **b)** 0.01 mmol of K[PPh_2], or **c)** 0.01 mmol of K[PhNNPh] added as a catalyst.

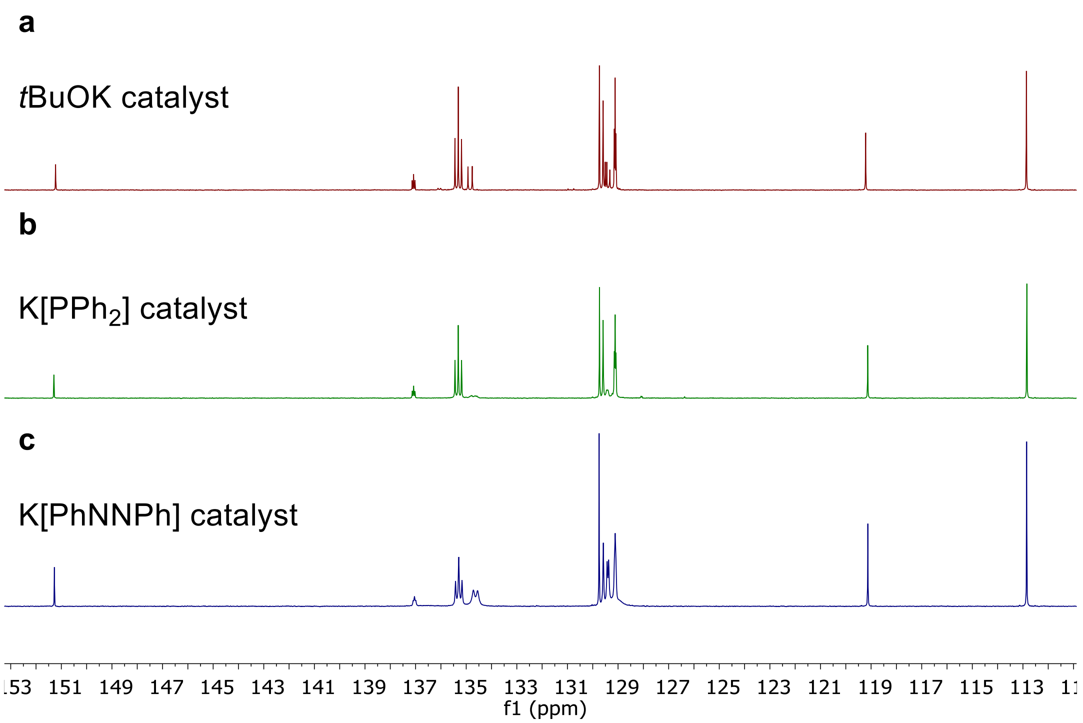

Supplementary Figure 15. Stacked $^{13}C\{^{1}H\}$ NMR spectra showing the formation of **2a** and hydrazobenzene after the reaction of 0.22 mmol of **1a** and 0.1 mmol of **HA-2** in THF-d_8 at 25 °C with either **a)** 0.01 mmol of *t*BuOK, **b)** 0.01 mmol of K[PPh_2], or **c)** 0.01 mmol of K[PhNNPh] added as a catalyst.

$^{13}C\{^{1}H\}$ NMR data for hydrazobenzene:
$^{13}C\{^{1}H\}$ NMR (101 MHz, THF-d_8) δ 151.26, 129.72, 119.20, 112.86.

$^{13}C\{^{1}H\}$ NMR data for **2a**:
^{13}C NMR (101 MHz, THF-d_8) δ 137.11 (t, J = 5.4 Hz), 135.33 (t, J = 13.1 Hz), 129.59, 129.12 (t, J = 3.4 Hz).

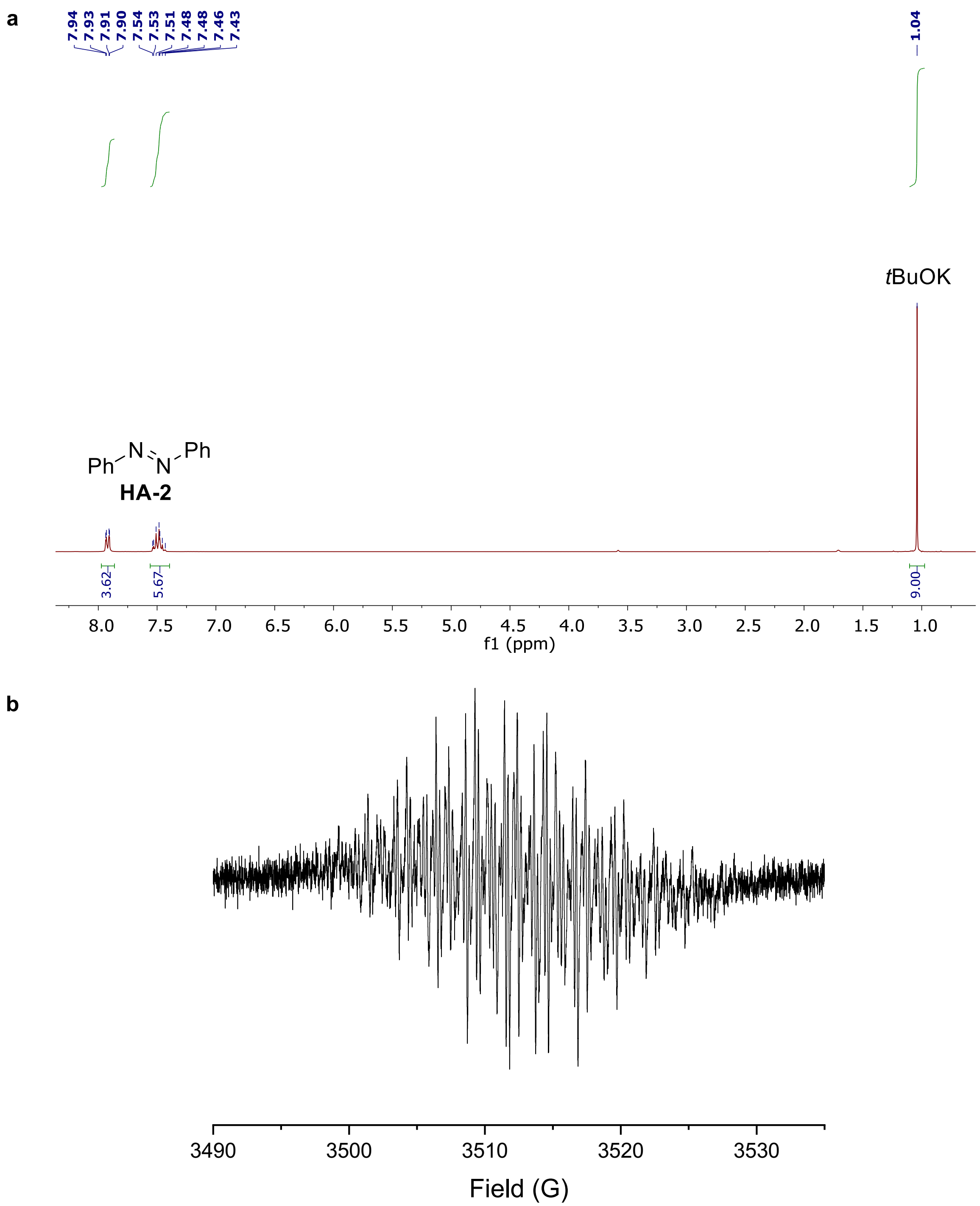

Supplementary Figure 16. a, ^{1}H NMR spectrum (300 MHz, 25 °C) recorded 16 h after mixing 0.1 mmol of **HA-2** with 0.1 mmol of *t*BuOK in 0.5 mL of THF-d_8. NMR spectrum shows unreacted **HA-2** and *t*BuOK, even though there was an immediate colour change from orange colour of **HA-2** to dark brown upon addition *t*BuOK. **b,** X-band EPR spectrum of K[PhNNPh] synthesised from the 1:1 stoichiometric reaction of **HA-2** with *t*BuOK, spectrum measured in THF:2-Me-THF (20:1) at ~5 mM concentration at 298 K with a microwave frequency of 9.870690 GHz.

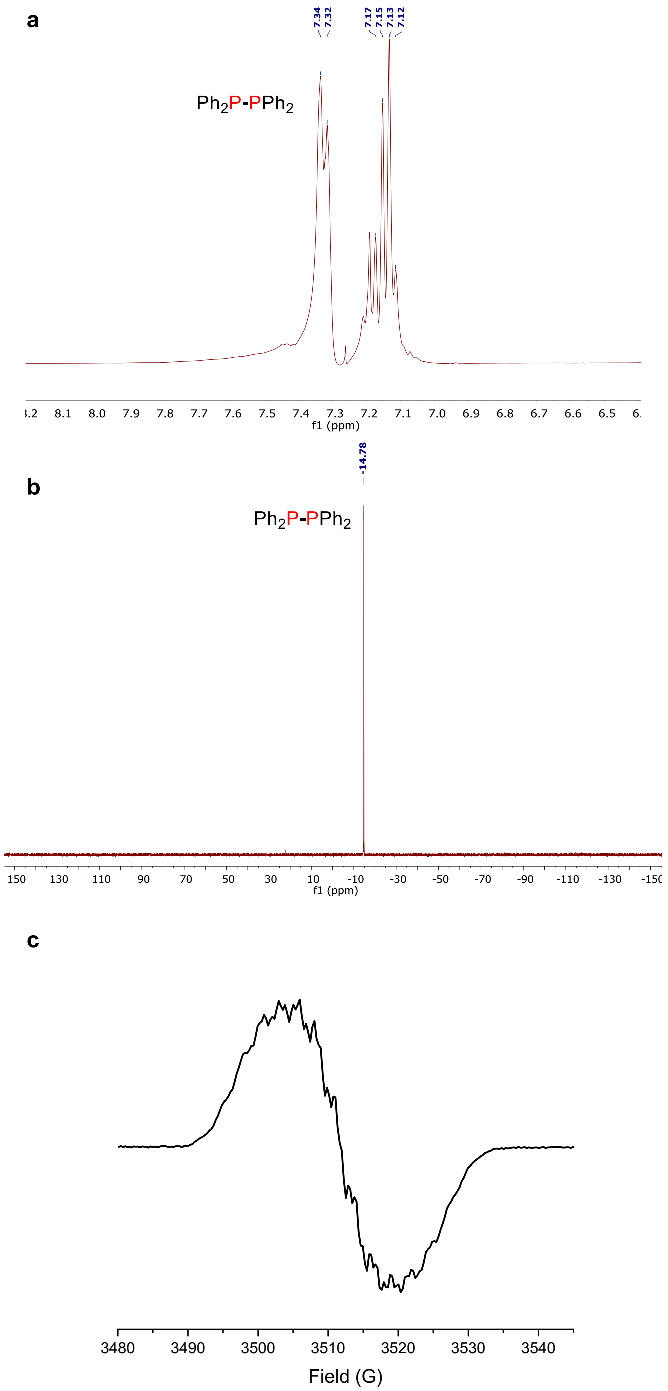

Supplementary Figure 17. a, Aromatic region of ^{1}H NMR spectrum (300 MHz, 25 °C) for **2a** and **b,** ^{31}P{^{1}H} NMR spectra (122 MHz, 25 °C) recorded after 5 min at 25 °C from the reaction of 0.1 mmol of **HA-2** with 0.1 mmol of K[PPh$_2$] (**1a**$^-$) in 0.5 mL of THF-d_8. NMR spectra show compound **2a** as the only diamagnetic species, and complete conversion of **HA-2** into an NMR-silent paramagnetic species (note lack of **HA-2** δ_H: multiplets at 7.94–7.90 and 7.54–7.43 ppm). **c,** X-band EPR spectrum of K[PhNNPh] from the 1:1 stoichiometric reaction of **HA-2** with **1a**$^-$, spectrum measured in 2-Me-THF at ~5 mM concentration at 298 K with a microwave frequency of 9.863949 GHz.

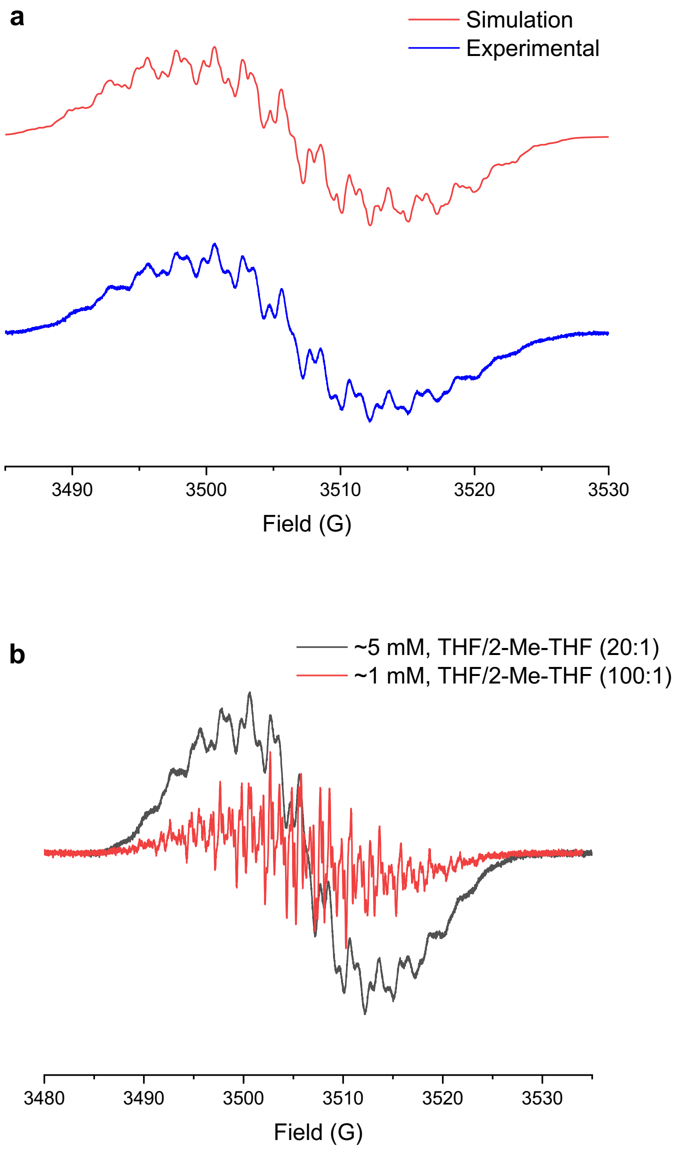

Supplementary Figure 18. a, (Experimental, bottom blue trace) X-band EPR spectrum of independently synthesised K[PhNNPh] from the reaction of potassium metal and **HA-2**, spectrum measured in THF/2-Me-THF (20:1) at ~5 mM concentration at 298 K with a microwave frequency of 9.851933 GHz. (Simulation, top red trace) Parameters used in simulation of EPR spectrum in EasySpin: g = 2.00746, $a(^{14}N)$ = 13.952 MHz, $a(^{1}H)$ = -6.093 MHz, $a(^{1}H)$ = 1.823 MHz, $a(^{1}H)$ = -8.747 MHz, $a(^{1}H)$ = -2.704 MHz, $a(^{1}H)$ = -7.778 MHz, $a(^{39}K)$ = 1.201 MHz, with Lorentzian line shapes with 0.05 mT (0.5 G) peak-to-peak line widths. **b,** Effect of concentration and solvent on X-band EPR spectrum of K[PhNNPh] at 298 K, grey trace (simulated above) is at ~5 mM concentration in THF/2-Me-THF (20:1) and red trace is at ~1 mM concentration in THF/2-Me-THF (100:1). Significant line broadenings are observed in more concentrated samples in solvent mixtures containing a higher proportion of 2-Me-THF, which is often less lossy and prone to absorption of microwaves than THF.

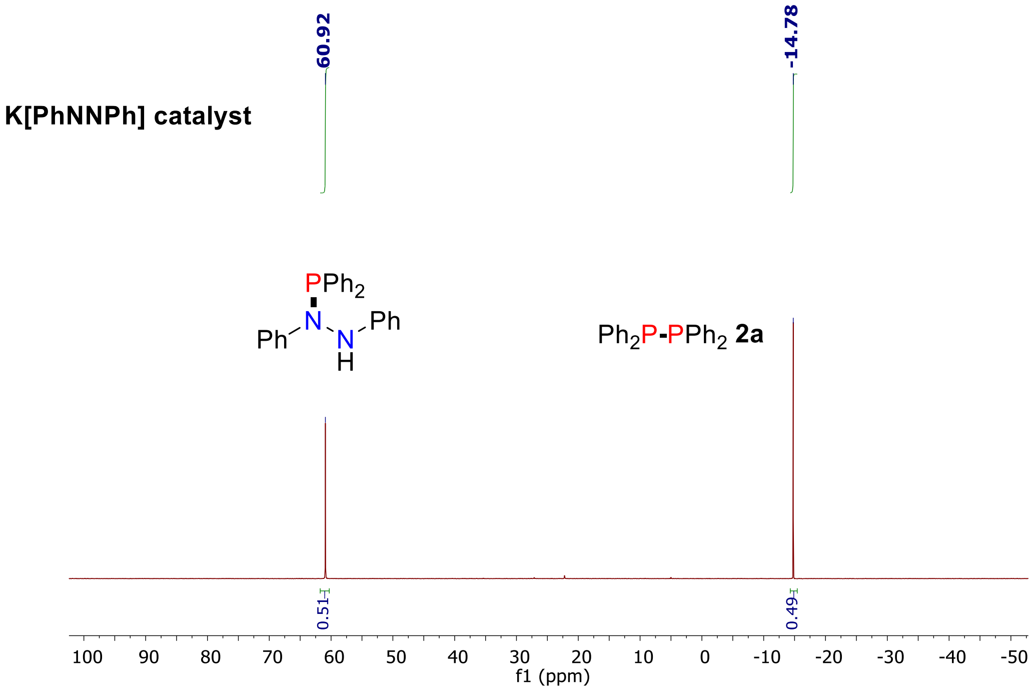

Supplementary Figure 19. $^{31}P\{^{1}H\}$ NMR spectrum recorded 5 min after reaction of 0.1 mmol of **1a** and 0.1 mmol of **HA-2** with 0.01 mmol of K[PhNNPh] at 25 °C in 0.5 mL of THF-d_8.

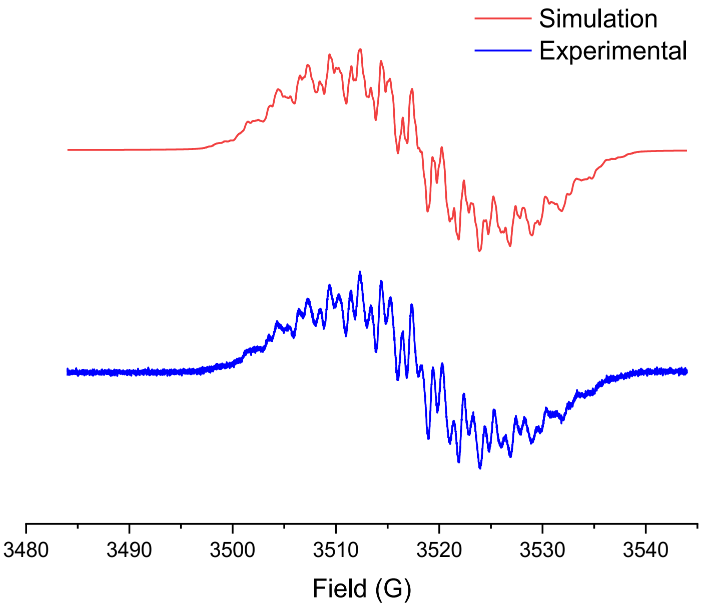

Supplementary Figure 20. (Experimental, bottom blue trace) X-band EPR spectrum of K[PhNNPh] from reaction of **HA-2** with 1 equiv. of *t*BuOK followed by the addition of 0.5 equiv. of **1a**, spectrum measured in THF/2-Me-THF (20:1) at 5 mM concentration at 298 K with a microwave frequency of 9.856775 GHz. (Simulation, top red trace) Parameters used in simulation of EPR spectrum in EasySpin: g = 2.00746, $a(^{14}N)$ = 13.984 MHz, $a(^{1}H)$ = -6.100 MHz, $a(^{1}H)$ = 1.850 MHz, $a(^{1}H)$ = -8.751 MHz, $a(^{1}H)$ = -2.665 MHz, $a(^{1}H)$ = -7.797 MHz, $a(^{39}K)$ = 1.181 MHz, with Lorentzian line shapes with 0.045 mT (0.45 G) peak-to-peak line widths.

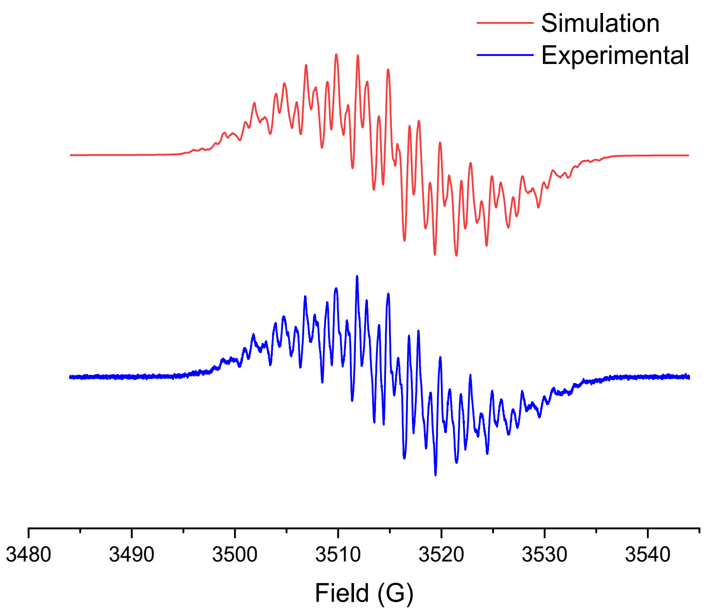

Supplementary Figure 21. (Experimental, bottom blue trace) X-band EPR spectrum of K[PhNNPh] from the 1:1 stoichiometric reaction of **7a** with *t*BuOK, spectrum measured in THF/2-Me-THF (20:1) at 5 mM concentration at 298 K with a microwave frequency of 9.849776 GHz. (Simulation, top red trace) Parameters used in simulation of EPR spectrum in EasySpin: *g* =2.00746, $a(^{14}N)$ = 14.083 MHz, $a(^{1}H)$ = -6.125 MHz, $a(^{1}H)$ = 1.949 MHz, $a(^{1}H)$ = -8.653 MHz, $a(^{1}H)$ = -2.566 MHz, $a(^{1}H)$ = -7.897 MHz, $a(^{39}K)$ = 1.081 MHz, with Lorentzian line shapes with 0.035 mT (0.35 G) peak-to-peak line widths.

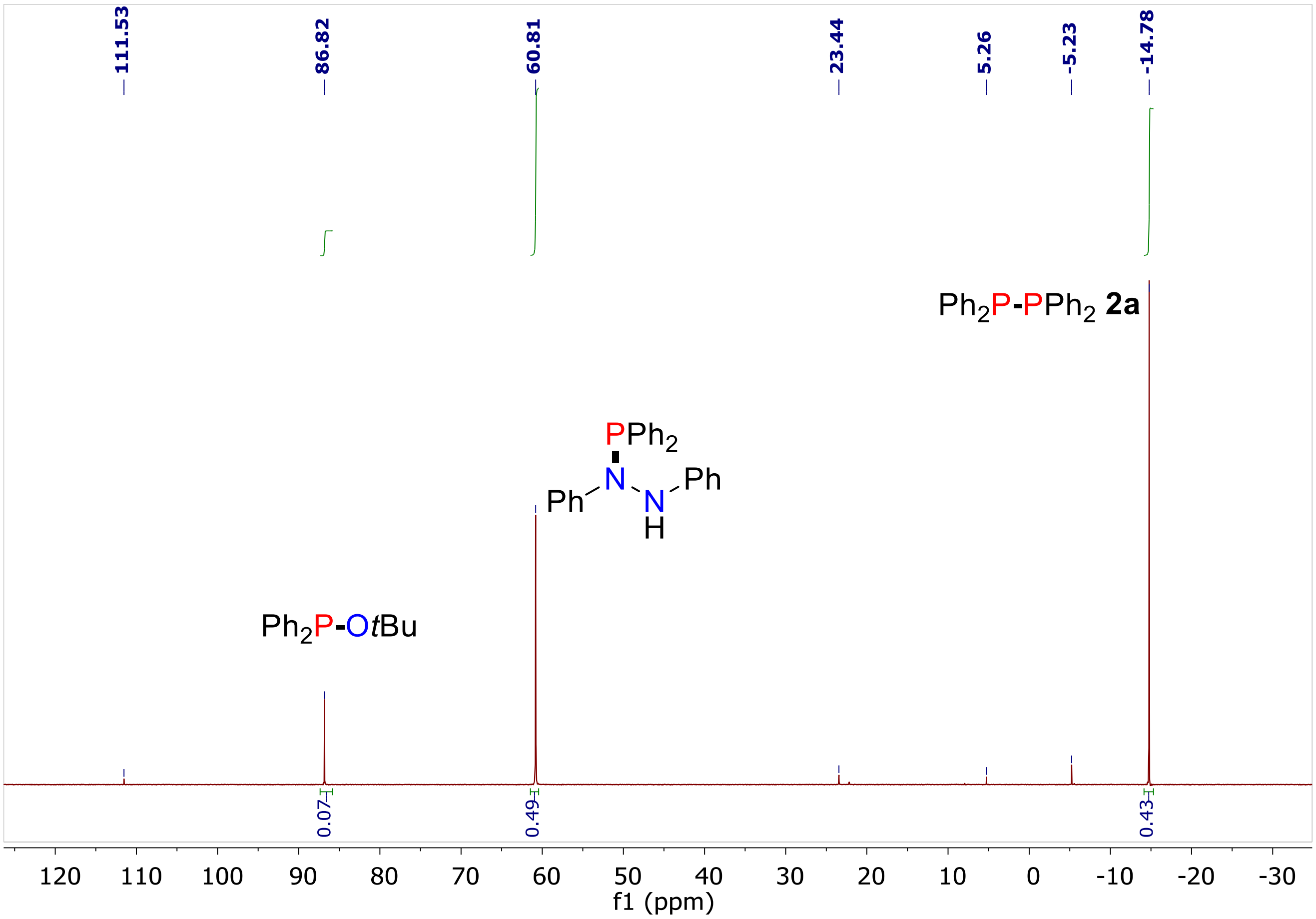

Supplementary Figure 22. $^{31}P\{^{1}H\}$ NMR spectrum (162 MHz, 25 °C) recorded 5 min after the reaction of 0.1 mmol of **7a** and 0.01 mmol of *t*BuOK in 0.5 mL of THF-d_8. Spectrum shows ~43 % conversion of **7a** to **2a**, and ~7% conversion to **5a**, along with other minor unidentified byproducts.

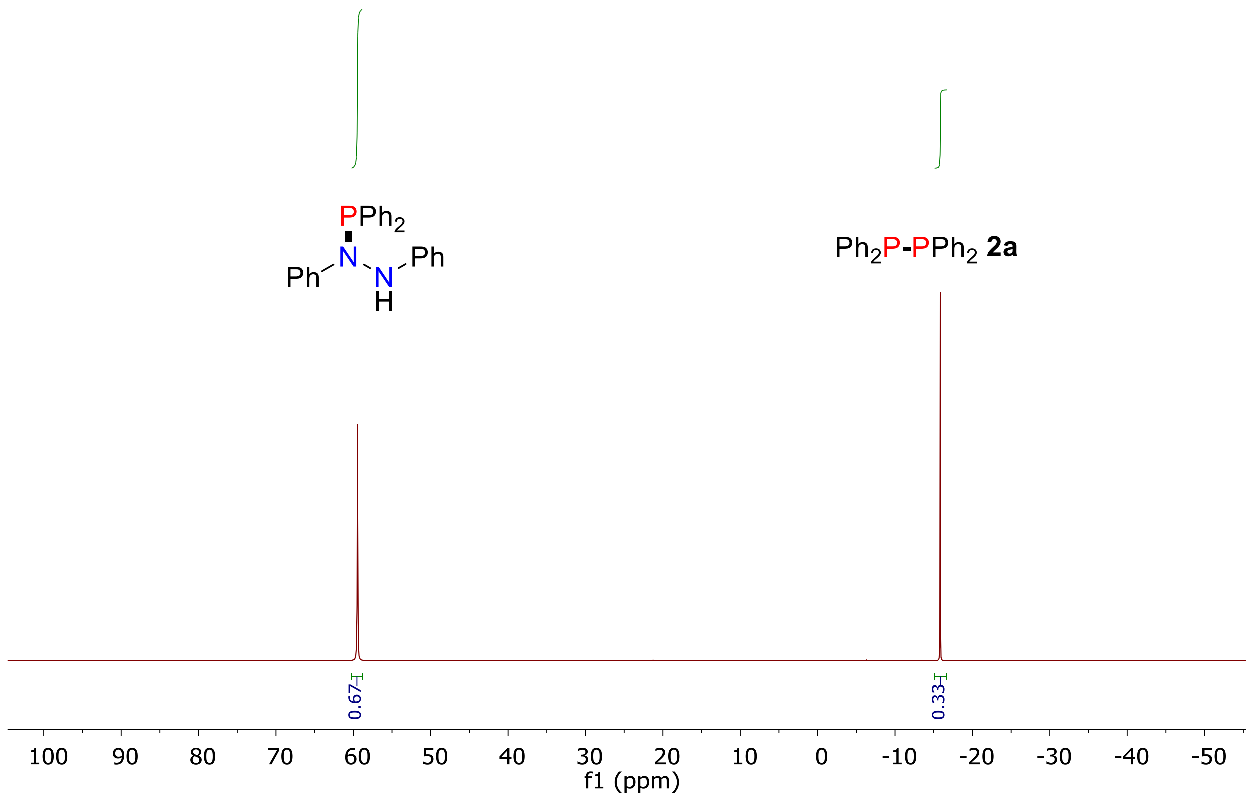

Supplementary Figure 23. $^{31}P\{^{1}H\}$ NMR spectrum (162 MHz, 25 °C) recorded 5 min after the reaction of 0.1 mmol of **7a** and 0.01 mmol of K[PPh$_2$] (**1a**$^-$) in 0.5 mL of THF-d_8. Spectrum shows ~33 % conversion of **7a** to **2a**.

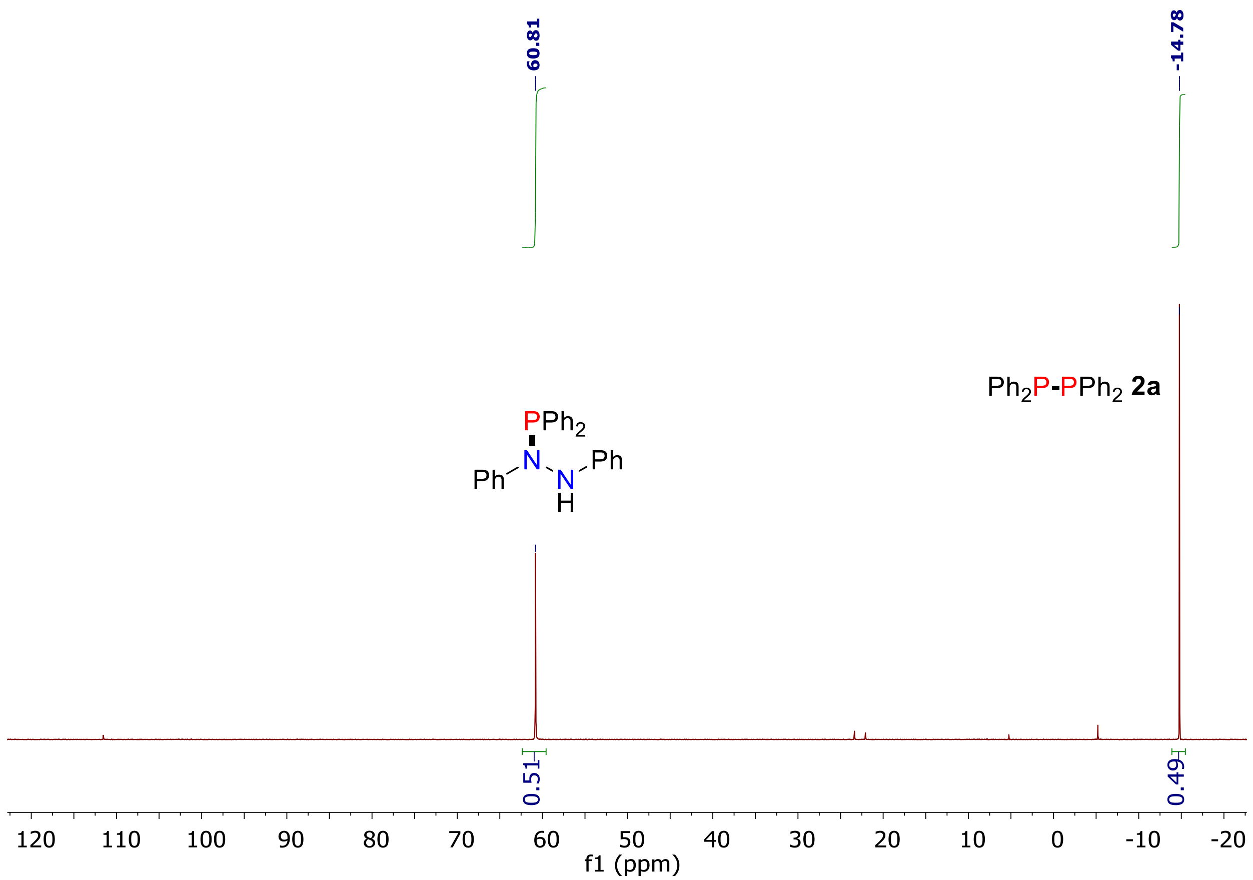

Supplementary Figure 24. $^{31}P\{^1H\}$ NMR spectrum (162 MHz, 25 °C) recorded 2 h after the reaction of 0.1 mmol of **7a** and 0.01 mmol of K[PhNNPh] in 0.5 mL of THF. Spectrum shows ~49 % conversion of **7a** to **2a**.

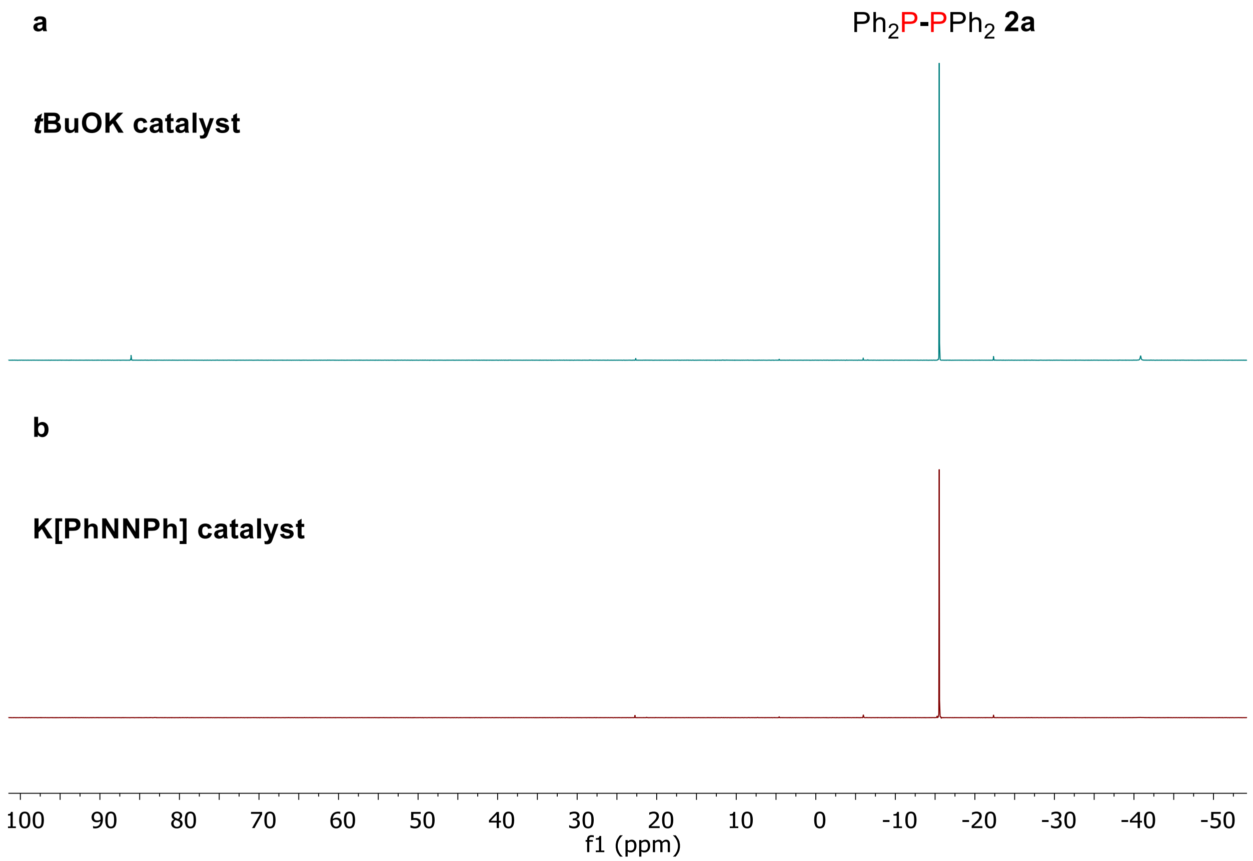

Supplementary Figure 25. a, $^{31}P\{^{1}H\}$ NMR spectrum recorded 5 min after reaction of 0.1 mmol of **1a** and 0.1 mmol of **7a** with 0.01 mmol of *t*BuOK added as a catalyst at 25 °C in 0.05 mL of THF-d_8. **b,** $^{31}P\{^{1}H\}$ NMR spectrum recorded 5 min after reaction of 0.1 mmol of **1a** and 0.1 mmol of **7a** with 0.01 mmol of K[PhNNPh] added as a catalyst at 25 °C in 0.5 mL of THF-d_8. Both NMR spectra show complete conversion of **7a** to **2a**.

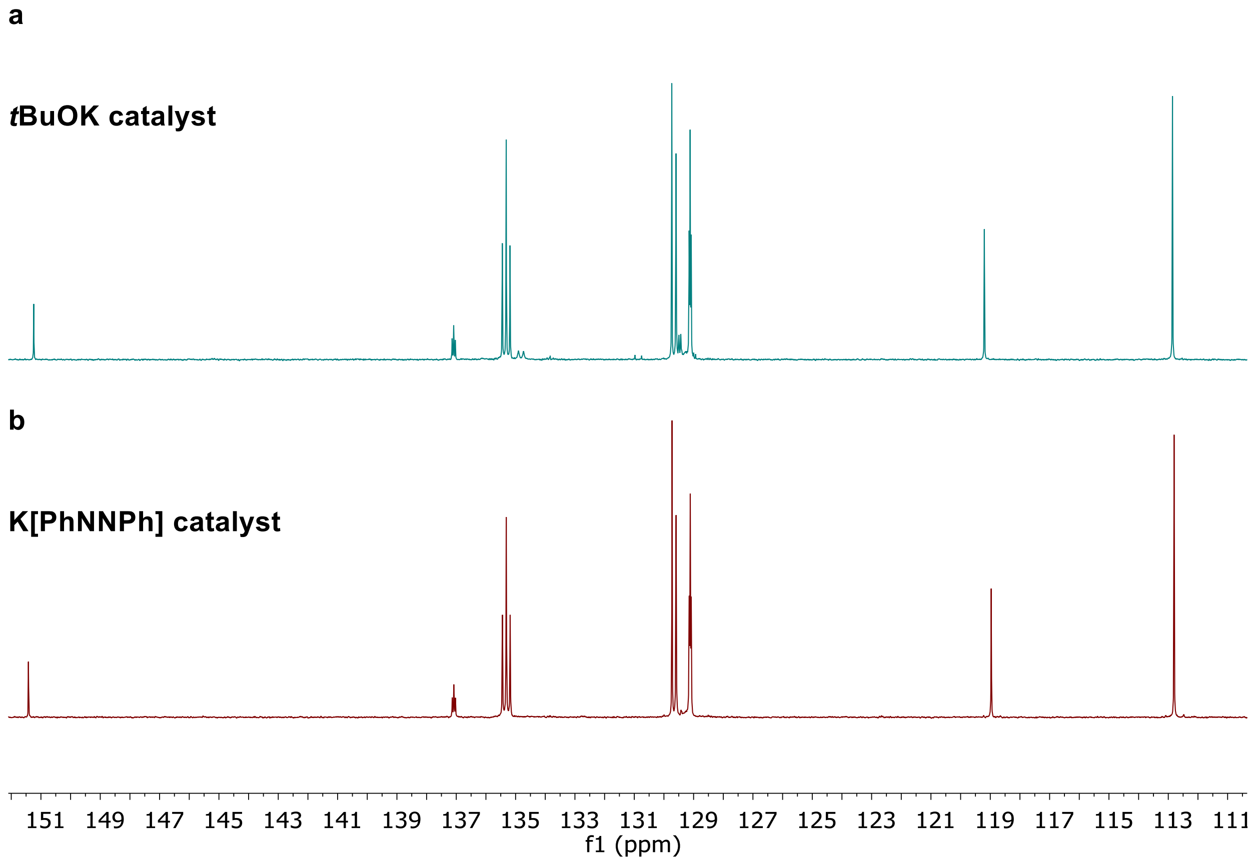

Supplementary Figure 26. a, $^{13}C\{^{1}H\}$ NMR spectrum recorded 5 min after reaction of 0.1 mmol of **1a** and 0.1 mmol of **7a** with 0.01 mmol of *t*BuOK added as a catalyst at 25 °C in 0.05 mL of THF-d_8. **b,** $^{13}C\{^{1}H\}$ NMR spectrum recorded 5 min after reaction of 0.1 mmol of **1a** and 0.1 mmol of **7a** with 0.01 mmol of K[PhNNPh] added as a catalyst at 25 °C in 0.5 mL of THF-d_8. Both NMR spectra show complete conversion of **7a** to **2a** and hydrazobenzene. See Supplementary Figure 15 for $^{13}C\{^{1}H\}$ NMR data for **2a** and hydrazobenzene.

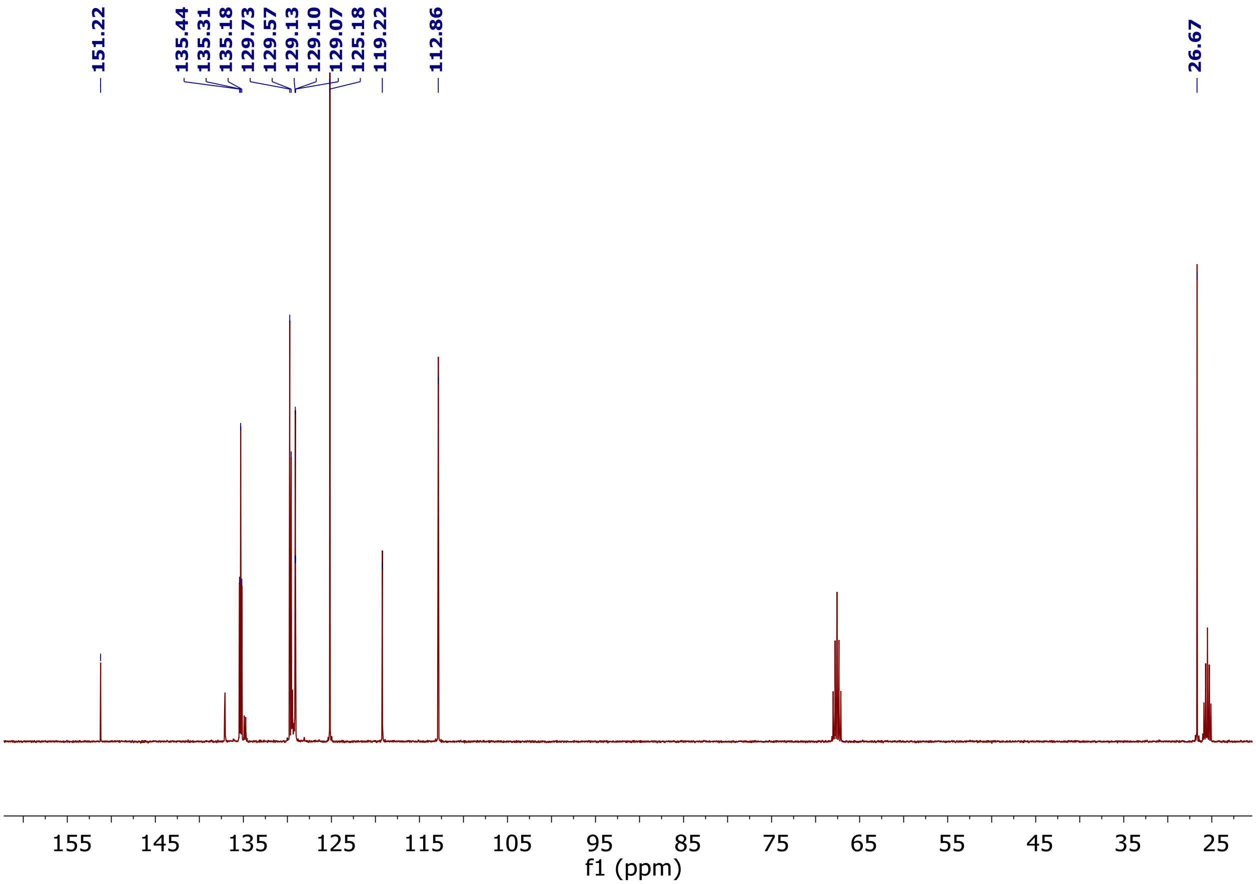

Supplementary Figure 27. $^{13}C\{^1H\}$ NMR spectrum from the attempted radical trapping experiment performed by adding 0.01 mmol of K[PhNNPh] to a mixture consisting of 0.5 mmol of 'radical trap' 1,4-cyclohexadiene, 0.2 mmol of **1a** and 0.1mmol of **HA-2** in THF-d_8. The spectrum displays a mixture of **2a**, hydrazobenzene and unreacted 1,4-cyclohexadiene, along with a lack of benzene formation at 128.84 ppm (the anticipated product of double H-atom abstraction from 1,4-cyclohexadiene).

Attempted radical trapping experiments were performed by adding 5 equiv. of the radical trap reagent 1,4-cyclohexadiene to a 2:1 stoichiometric mixture of **1a** and **HA-2** in THF. Upon the addition of 10 mol% of either *t*BuOK or K[PhNNPh] to the preceding mixture the full conversion to **2a** and hydrazobenzene and the complete lack of the H-atom abstraction product (benzene) was observed by $^{13}C\{^1H\}$ NMR (Fig. S27). The lack of formation of benzene even in the presence of added K[PhNNPh] radical anion in these experiments is suggestive that the secondary phosphine **1a** is a far more efficient radical trap than 1,4-cyclohexadiene and far more likely to engage in H-atom abstraction.[13]

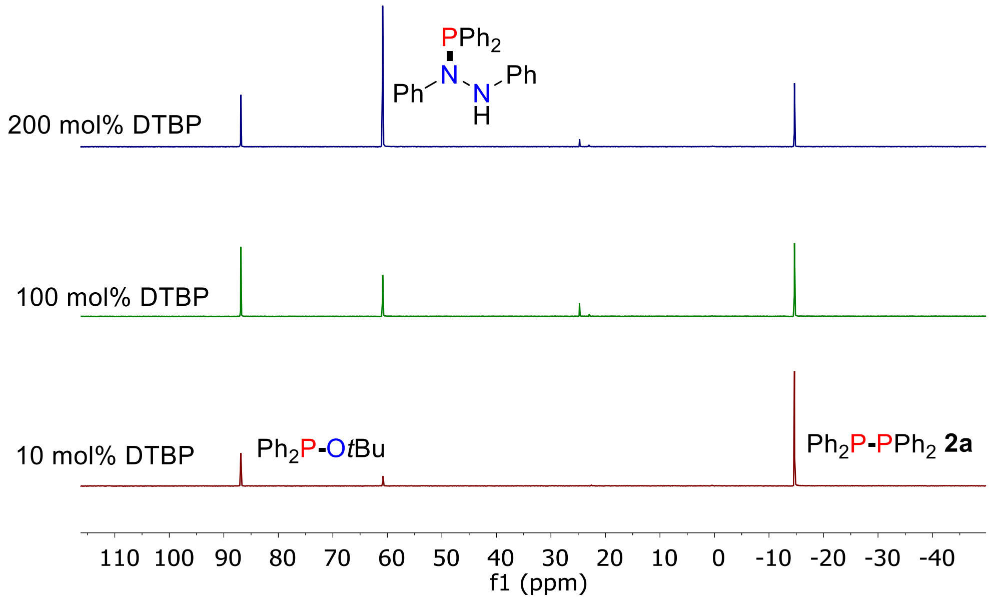

Supplementary Figure 28. $^{31}P\{^1H\}$ NMR spectra (THF) of the *t*BuOK-catalysed homodehydrocoupling of **1a** in the presence of **HA-2** with the addition of different amount of DTBP: reactions were performed with 0.1 mmol Ph_2PH, 0.01 mmol *t*BuOK, 0.1 mmol **HA-2** and 10 – 200 mol% DTBP with 0.5 mL THF in a J. Young NMR tube, 25 °C.

We can see from the above Figure, with more DTBP (radical source) the **HA-2** mediated homodehydrocoupling reaction of **1a** produced more **HA-2** hydrophosphination product and less dehydrocoupling product **2a** together with the fact that hydrophosphination product cannot easily be converted into **2a** support a radical mechanism for **HA-2** as hydrogen acceptor.

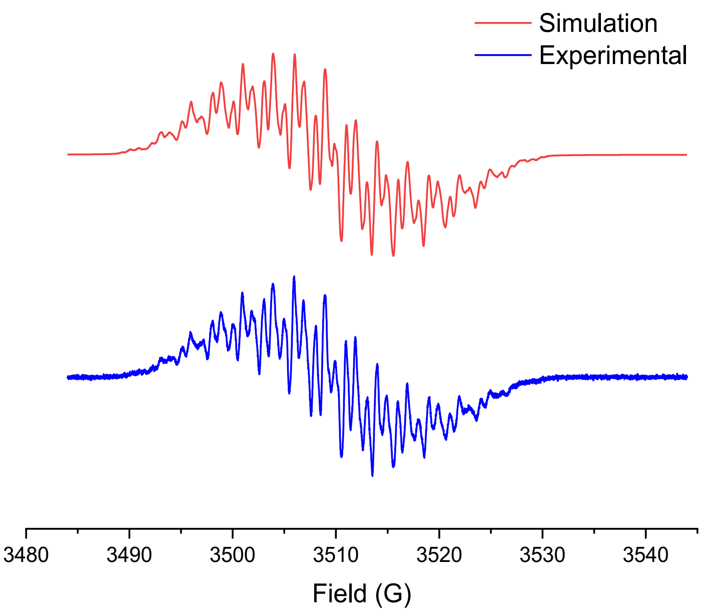

Supplementary Figure 29. (Experimental, bottom blue trace) X-band EPR spectrum of K[PhNNPh] from the 1:1 stoichiometric reaction of *t*BuOK and hydrazobenzene, spectrum measured in THF/2-Me-THF (20:1) at ~5 mM concentration at 298 K with a microwave frequency of 9.861312 GHz. (Simulation, top red trace) Parameters used in simulation of EPR spectrum in EasySpin: g = 2.00746, $a(^{14}N)$ = 14.083 MHz, $a(^{1}H)$ = -6.125 MHz, $a(^{1}H)$ = 1.949 MHz, $a(^{1}H)$ = -8.653 MHz, $a(^{1}H)$ = -2.566 MHz, $a(^{1}H)$ = -7.897 MHz, $a(^{39}K)$ = 1.081 MHz, with Lorentzian line shapes with 0.035 mT (0.35 G) peak-to-peak line widths.

Supplementary Discussion

Computational methods

All calculations were carried out by using the Gaussian 16 program.[14] All structures were optimised at the M062X[15] level of density functional theory (DFT) with the TZVP[16] basis set. All optimised structures were characterised either as energy minima without imaginary frequencies or transition states with only one imaginary frequency by frequency calculations; and the imaginary mode connects the initial and the final states. The thermal corrections to Gibbs free energy at 298 K from the frequency analysis are added to the total electronic energy, and we therefore used the corrected Gibbs free energy (ΔG) at 298 K for our discussions and comparisons. In our previous study,[17] we have done benchmark calculations comparing different functionals on the basis of the experimental observation and found that the M062X method gave the best agreement between theory and experiment in phosphine related chemistry; and this is also the reason for our choice to use M062X in our current study. In our computations we always used the whole molecules without any simplification or constraints.

Coupling reaction free energy

It is found that the direct homocoupling of Ph_2PH to Ph_2P-PPh_2 without hydrogen acceptor [2 equiv. $Ph_2PH = Ph_2P\text{-}PPh_2 + H_2$] is endergonic by 2.64 kcal·mol^{-1}, the thermodynamic equilibrium under stoichiometric conditions is less than 2% product; and therefore, hydrogen acceptor or other oxidants are needed to shift this reaction towards product formation. In the presence of an **HA** it is found that all of the dehydrocoupling reactions of 2 equiv. of **1a** to **2a** becomes exergonic and therefore thermodynamically downhill (Supplementary Figure 30). Apart from benzophenone (**HA-1**) with lower exergonic reaction energy (–3.48 kcal·mol^{-1}), other hydrogen acceptors have higher exergonic reaction energies (–10 to –13 kcal·mol^{-1}).

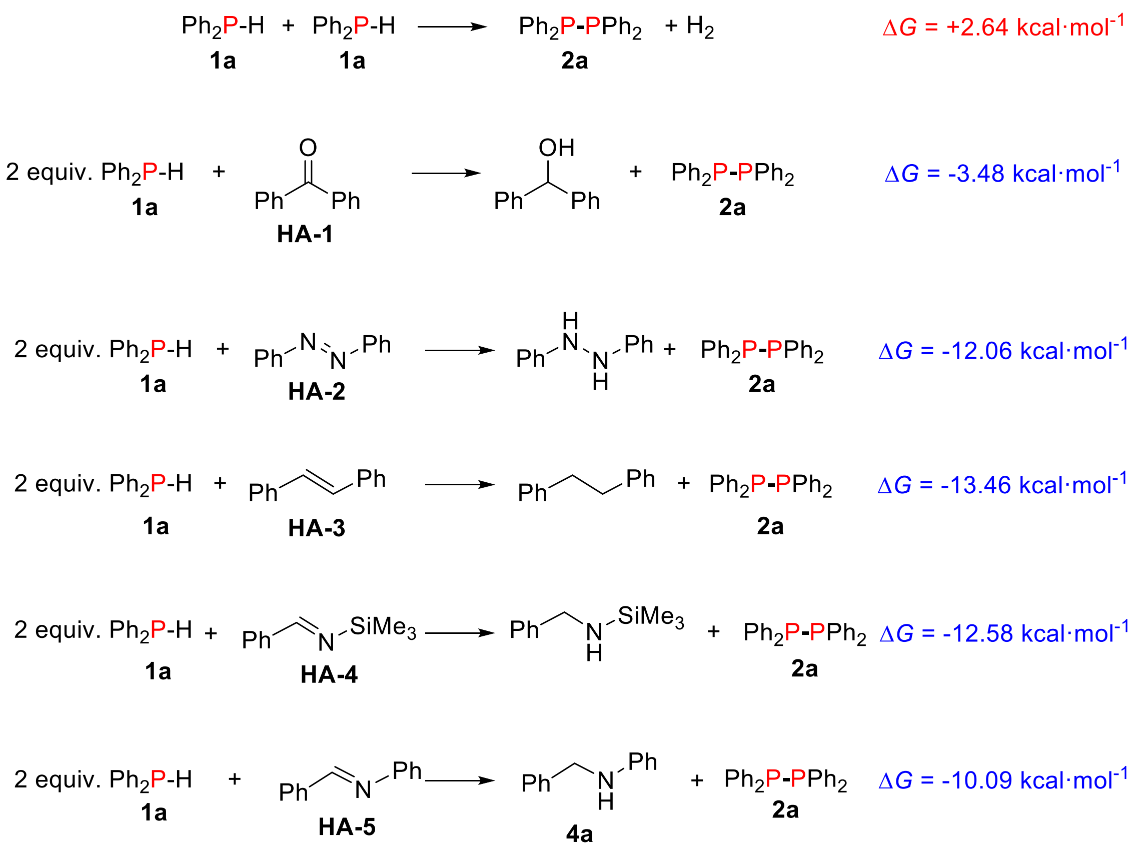

Supplementary Figure 30. M062X computed reaction Gibbs free energies for dehydrocoupling of **1a** in absence and presence of different **HA**s.

We computed the consecutive steps of the reaction by using *N*-benzylideneaniline (**HA-5**) as the hydrogen acceptor (Supplementary Figure 31). The first step is hydrophosphination and the second step is coupling. The first step is exergonic by 4.03 kcal·mol^{-1} and the second step is exergonic by 6.06 kcal·mol^{-1}; and the total reaction is exergonic by 10.09 kcal·mol^{-1}. The product of hydrophosphination is calculated to be an important intermediate, in agreement with the experimental results. It is also noted that the heterodehydrocoupling between **1a** and *t*BuOH is endergonic by 2.11 kcal·mol^{-1}, similar to the value calculated for P-P homocoupling without hydrogen acceptor, but becomes exergonic (–10.62 kcal·mol^{-1}) with hydrogen acceptor. The closeness in reaction Gibbs free energies between P-P and P-O coupling (–10.09 vs. –10.62 kcal·mol^{-1}) suggests that both reactions are possible and competitive; and this is also in agreement with the experimental results.

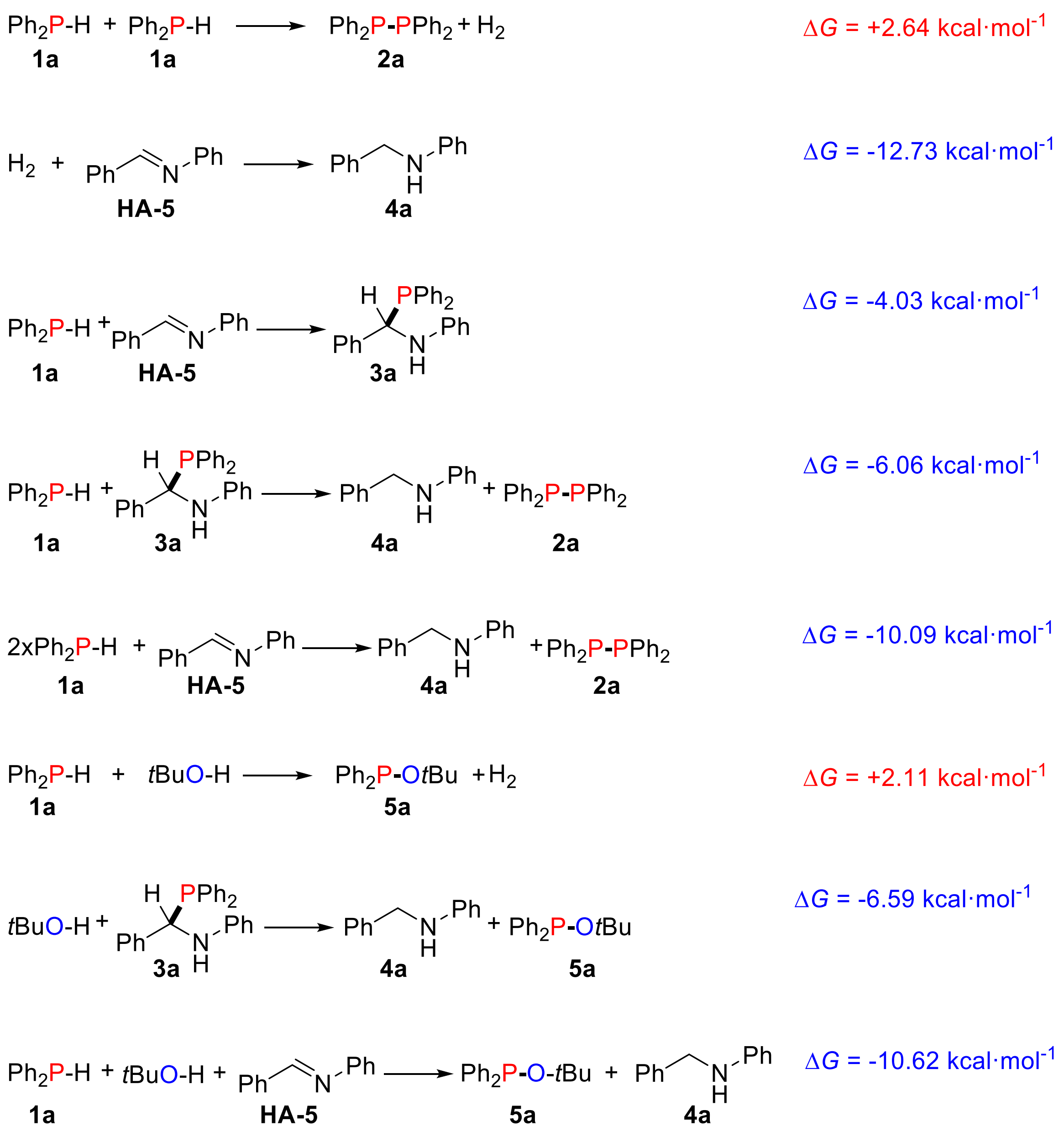

Supplementary Figure 31. M062X computed reaction Gibbs free energies for reactions involved in homodehydrocoupling and heterodehydrocoupling of **1a** with **HA-5**.

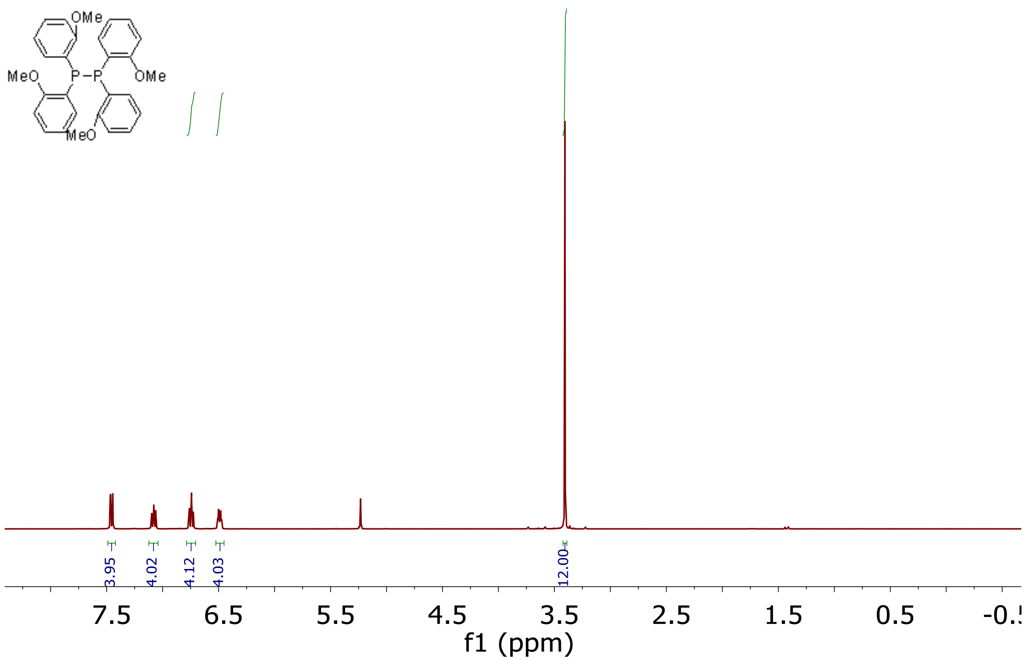

Supplementary Figure 32. ^{1}H NMR spectrum of **2e** (Methylene Chloride-d_2)

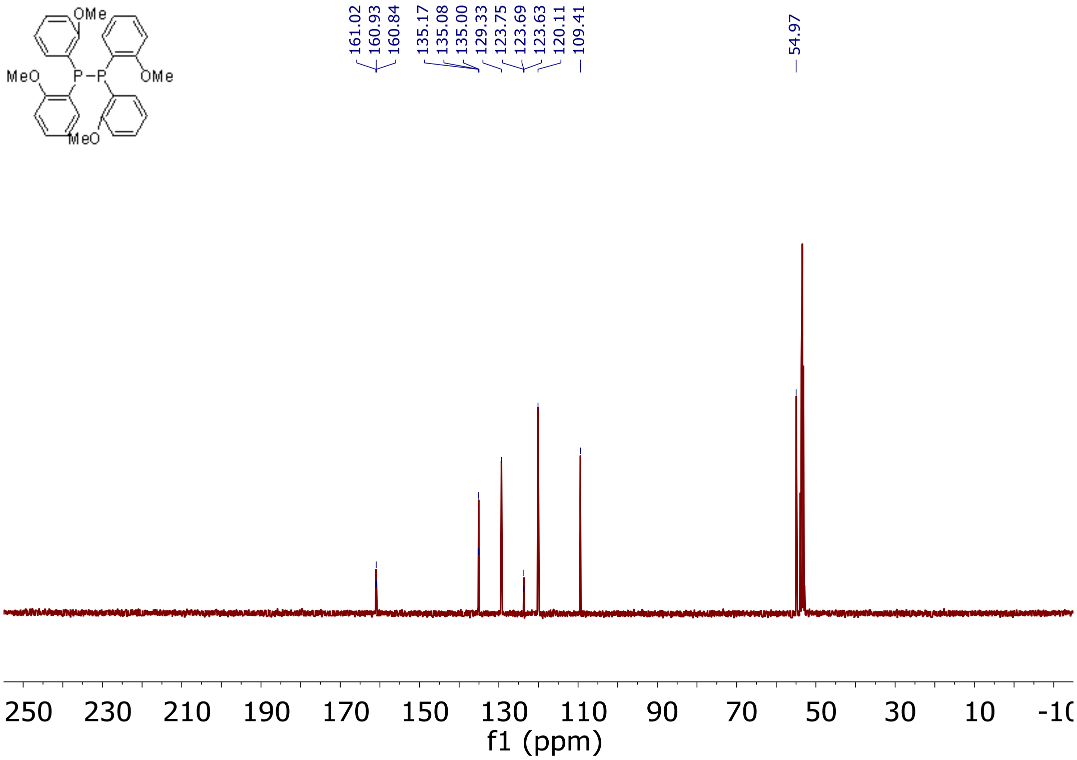

Supplementary Figure 33. ^{13}C NMR spectrum of **2e** (Methylene Chloride-d_2)

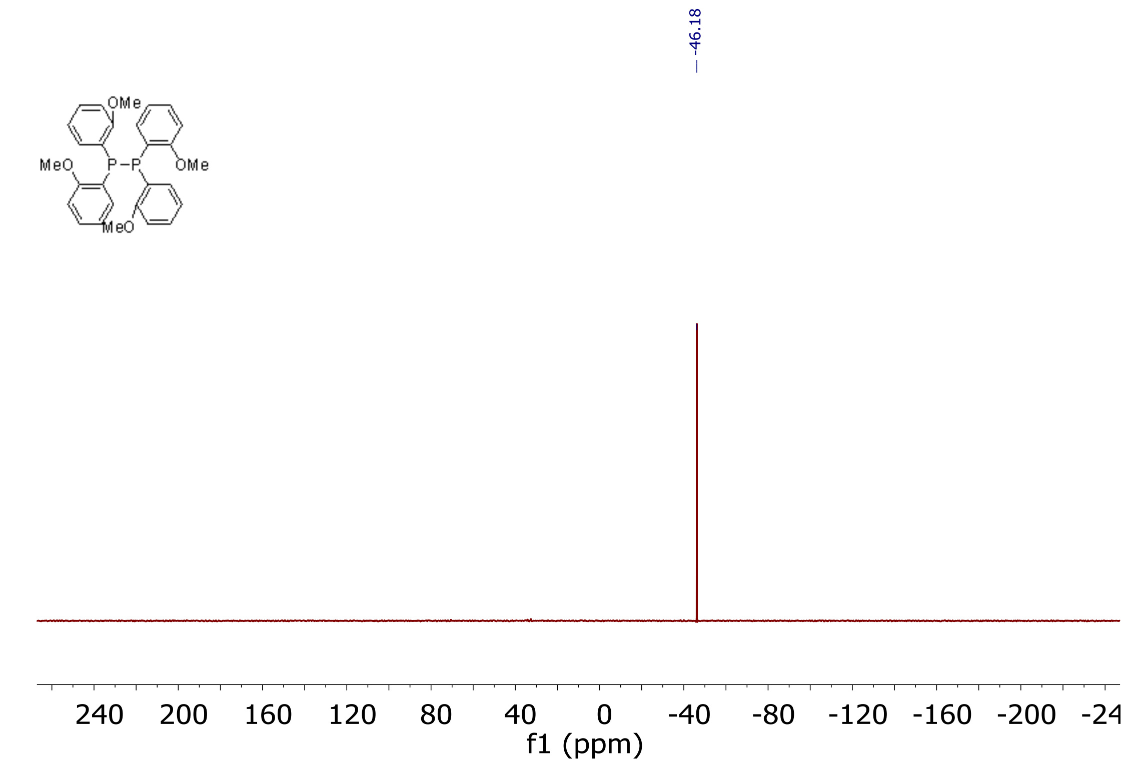

Supplementary Figure 34. $^{31}P\{^{1}H\}$ NMR spectrum of **2e** (Methylene Chloride-d_2)

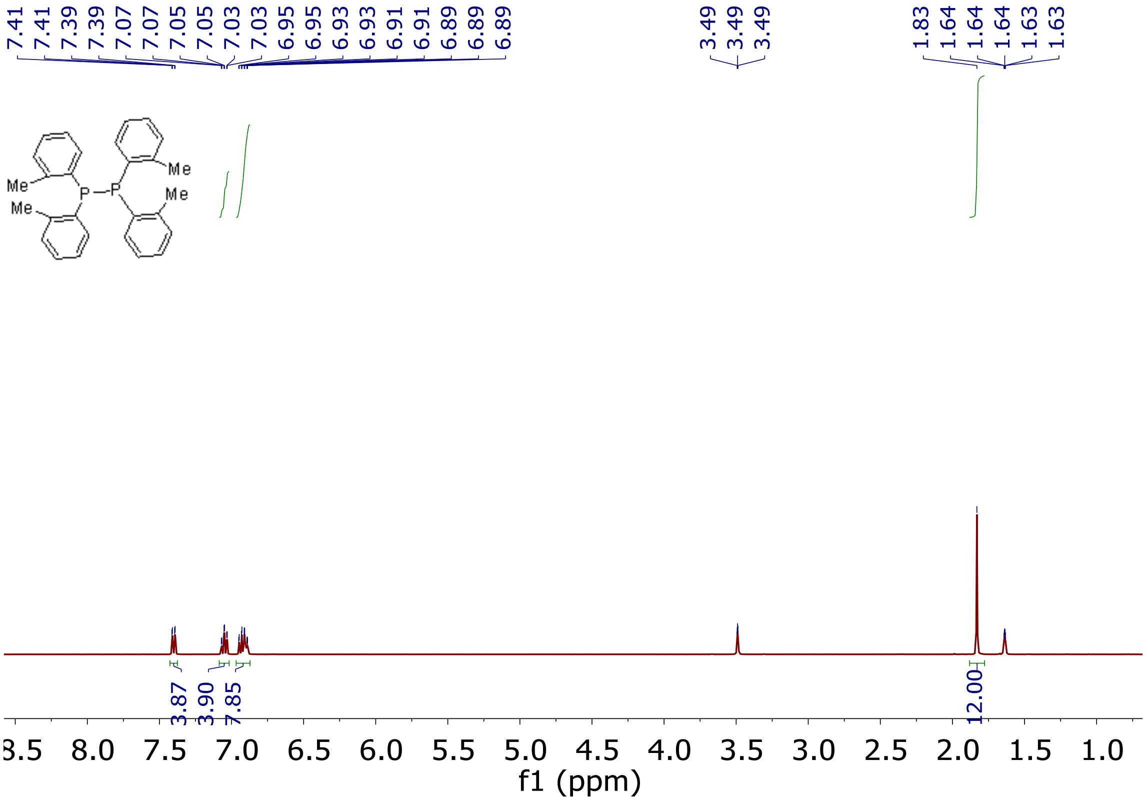

Supplementary Figure 35. ^{1}H NMR spectrum of **2f** (THF-d_8)

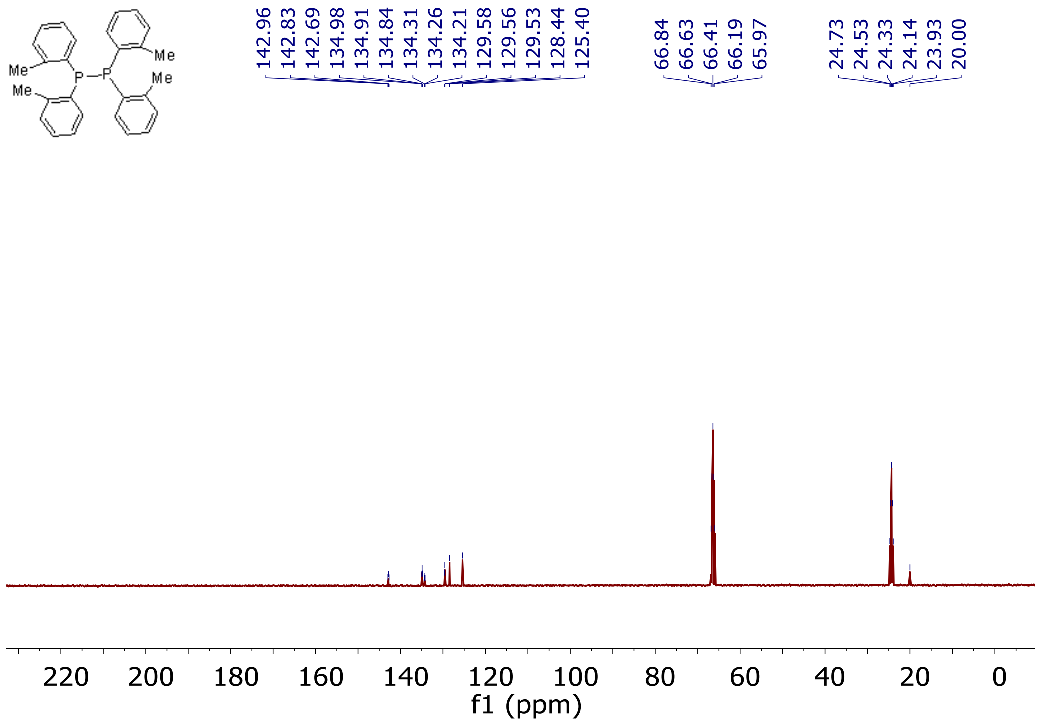

Supplementary Figure 36. ^{13}C NMR spectrum of **2f** (THF-d_8)

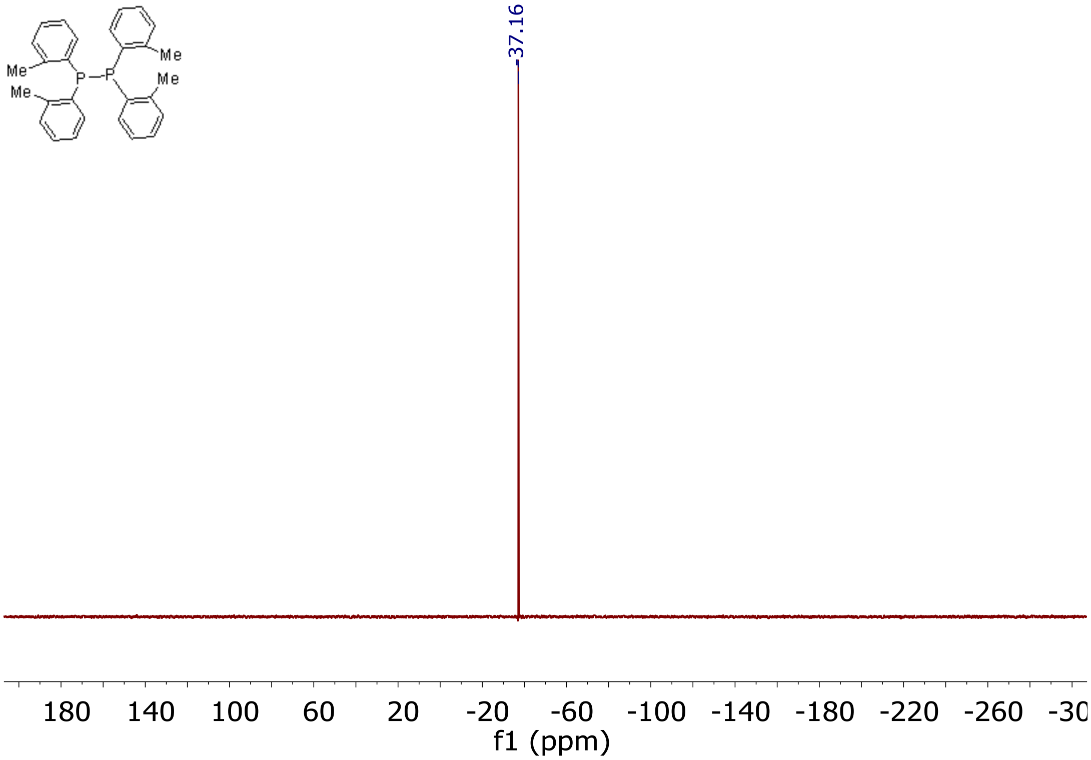

Supplementary Figure 37. $^{31}P\{^1H\}$ NMR spectrum of **2f** (THF-d_8)

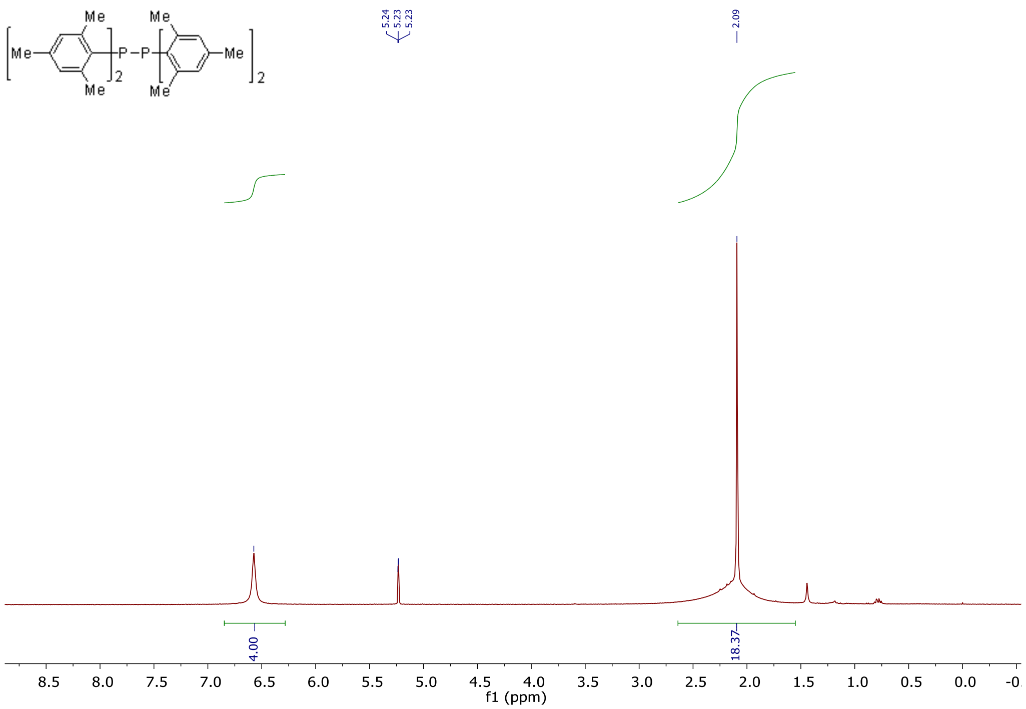

Supplementary Figure 38. ^{1}H NMR spectrum of **2h** (Methylene Chloride-d_2)

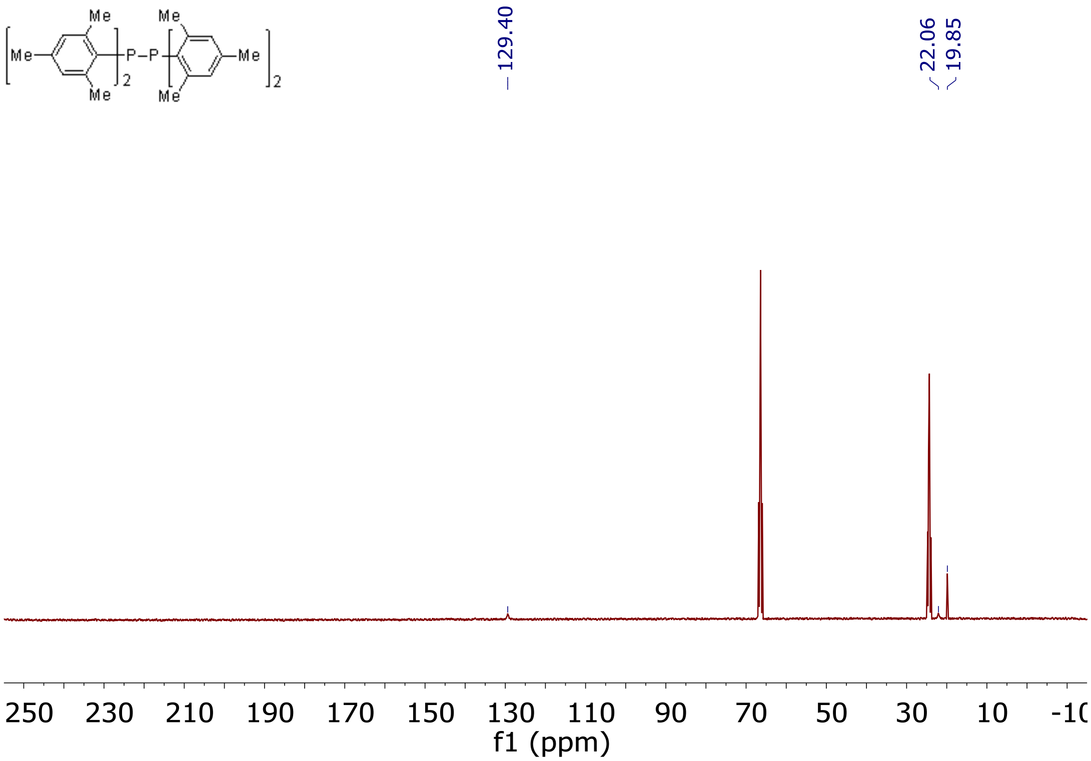

Supplementary Figure 39. ^{13}C NMR spectrum of **2h** (THF-d_8)

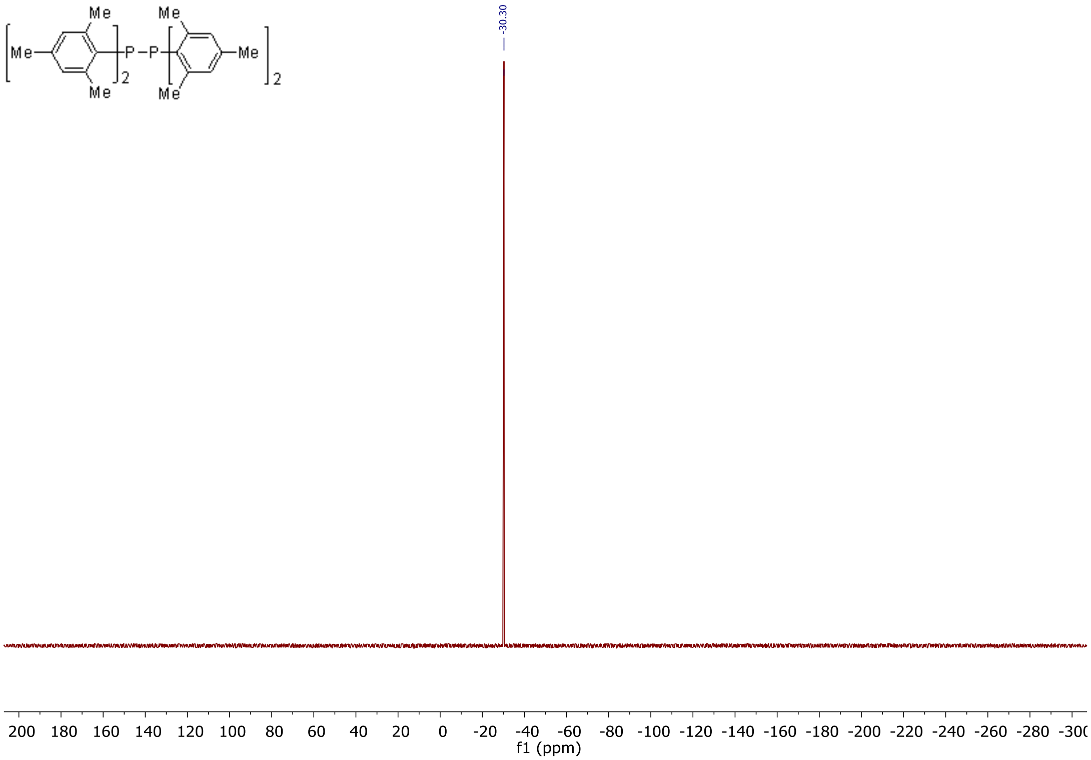

Supplementary Figure 40. ^{13}P{^{1}H} NMR spectrum of **2h** (Methylene Chloride-d_2)

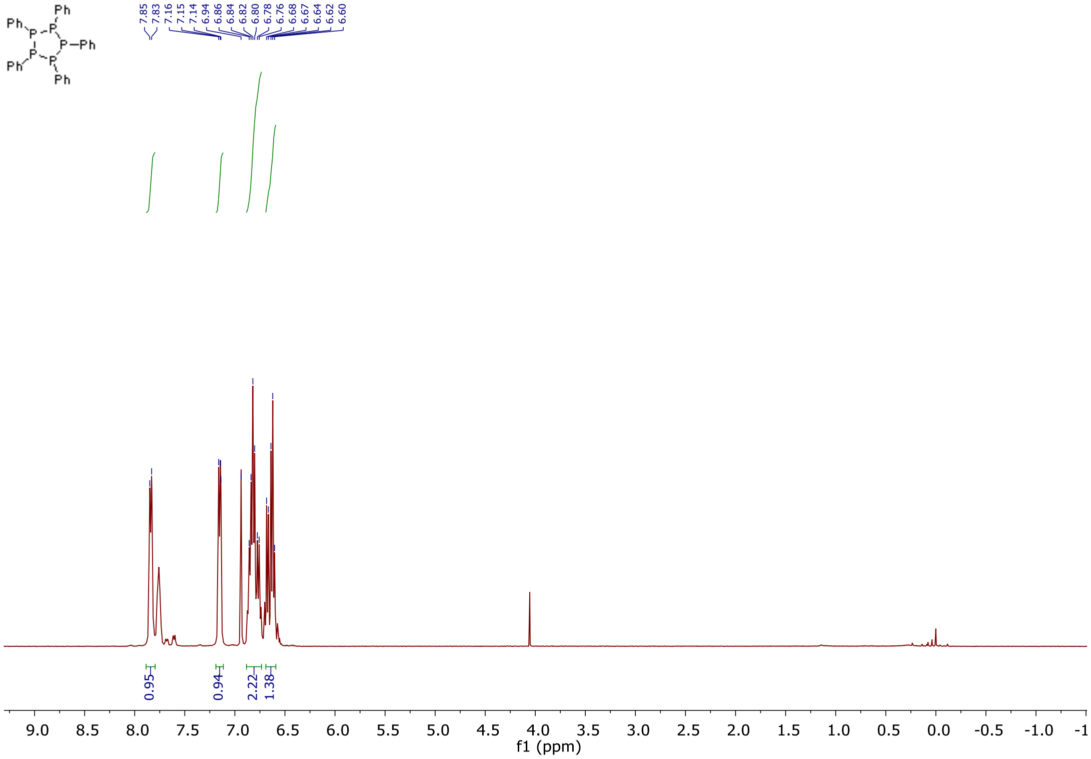

Supplementary Figure 41. ^{1}H NMR spectrum of **2i** (Benzene-d_6)

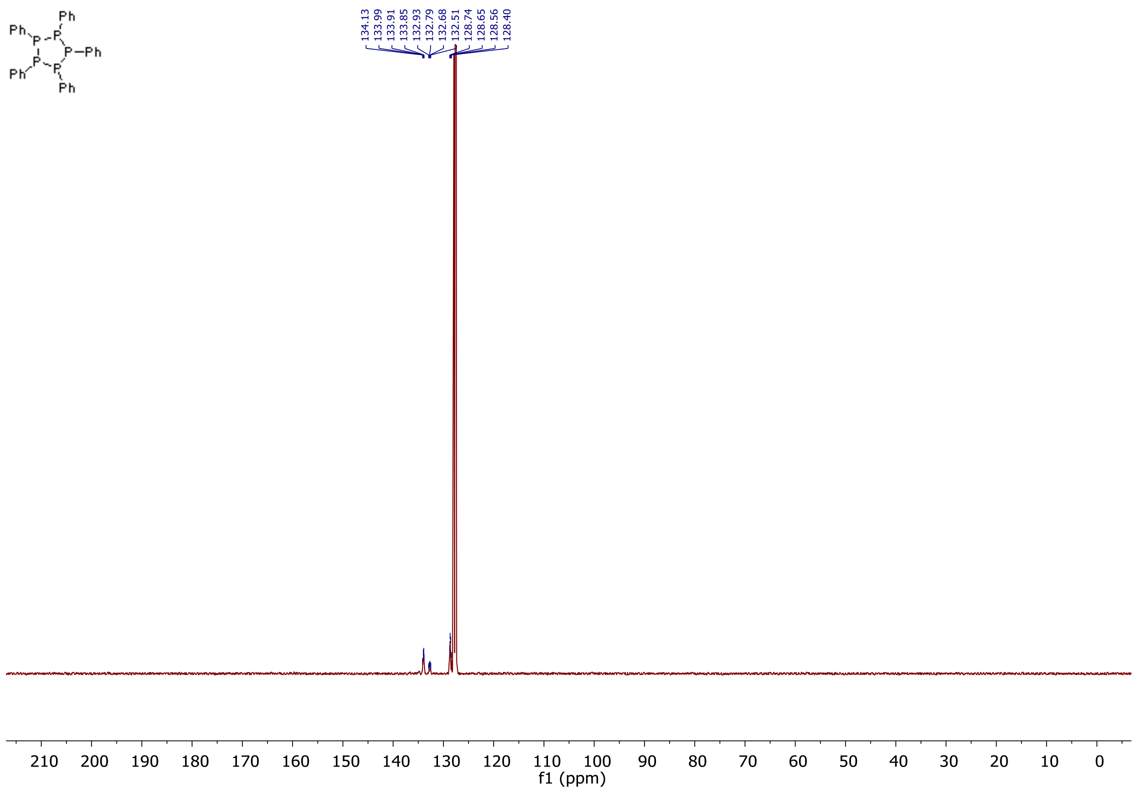

Supplementary Figure 42. ^{13}C NMR spectrum of **2i** (Benzene-d_6)

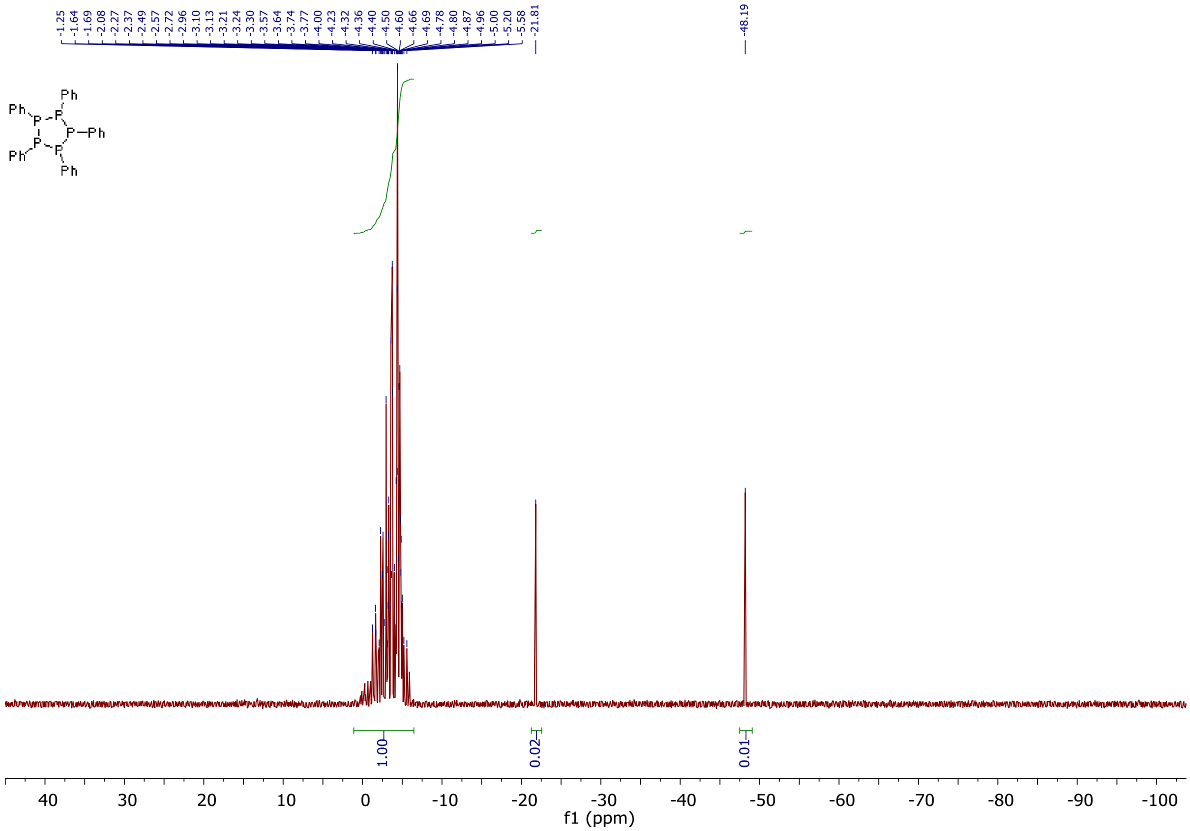

Supplementary Figure 43. ^{31}P{^{1}H} NMR spectrum of **2i** (Benzene-d_6)

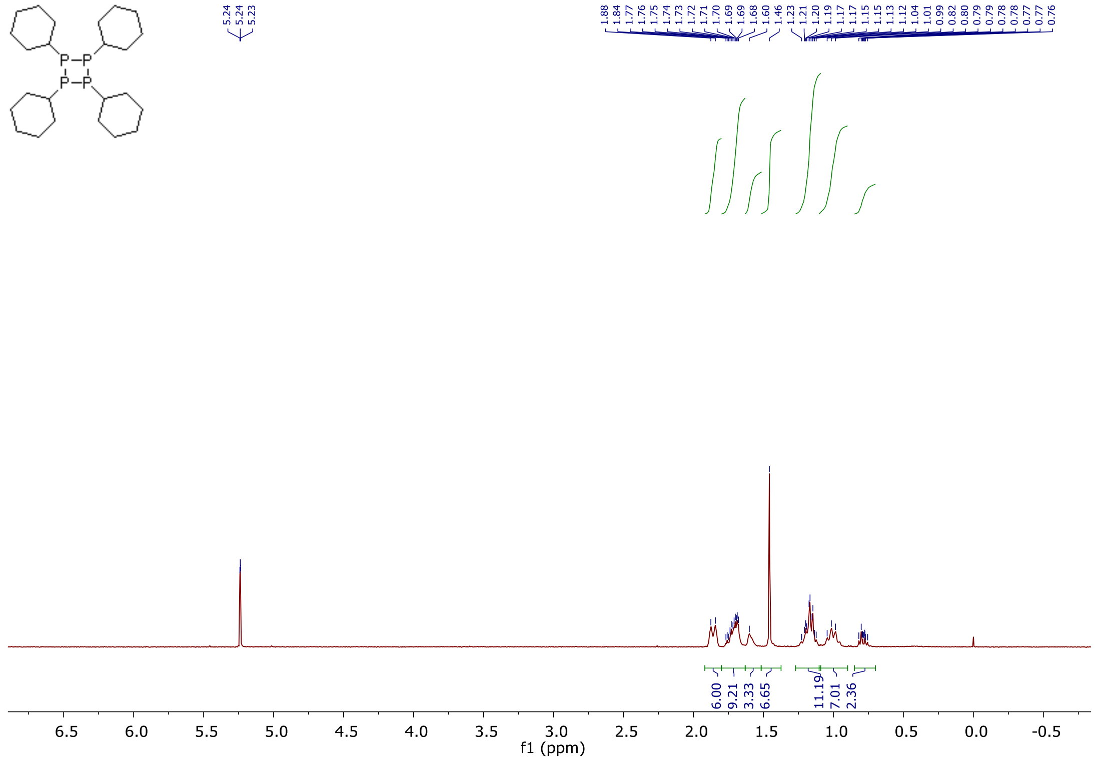

Supplementary Figure 44. ^{1}H NMR spectrum of **2j** (Methylene Chloride-d_2)

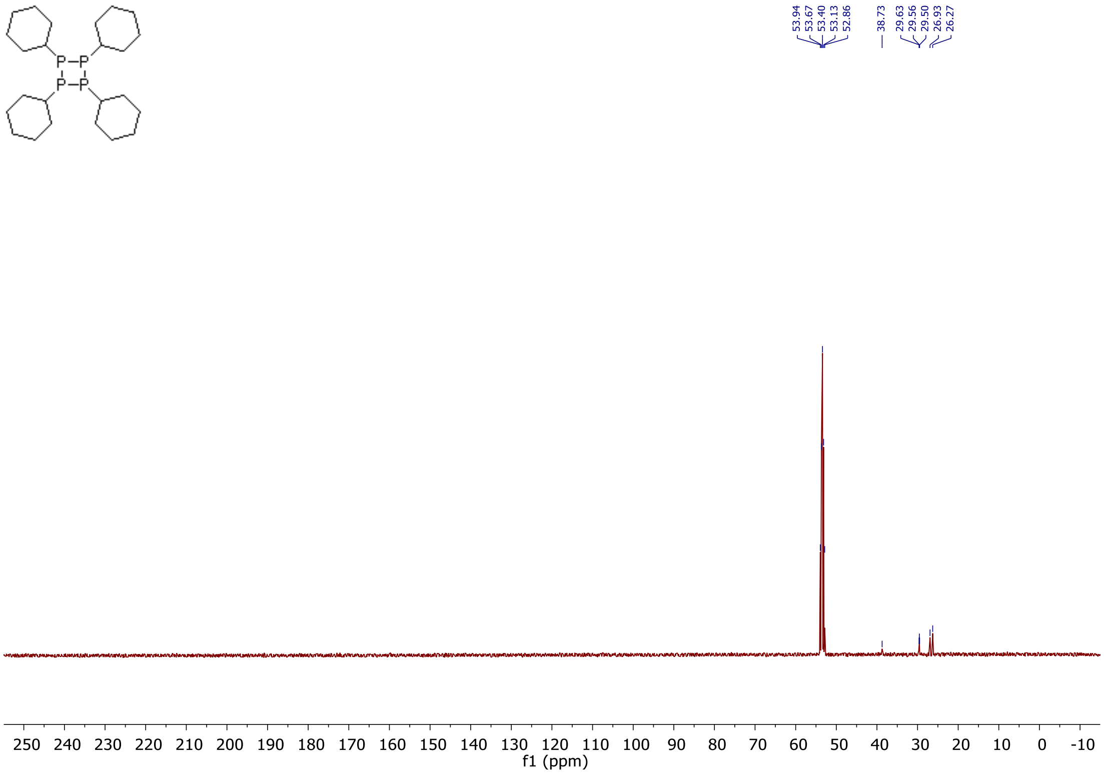

Supplementary Figure 45. ^{13}C NMR spectrum of **2j** (Methylene Chloride-d_2)

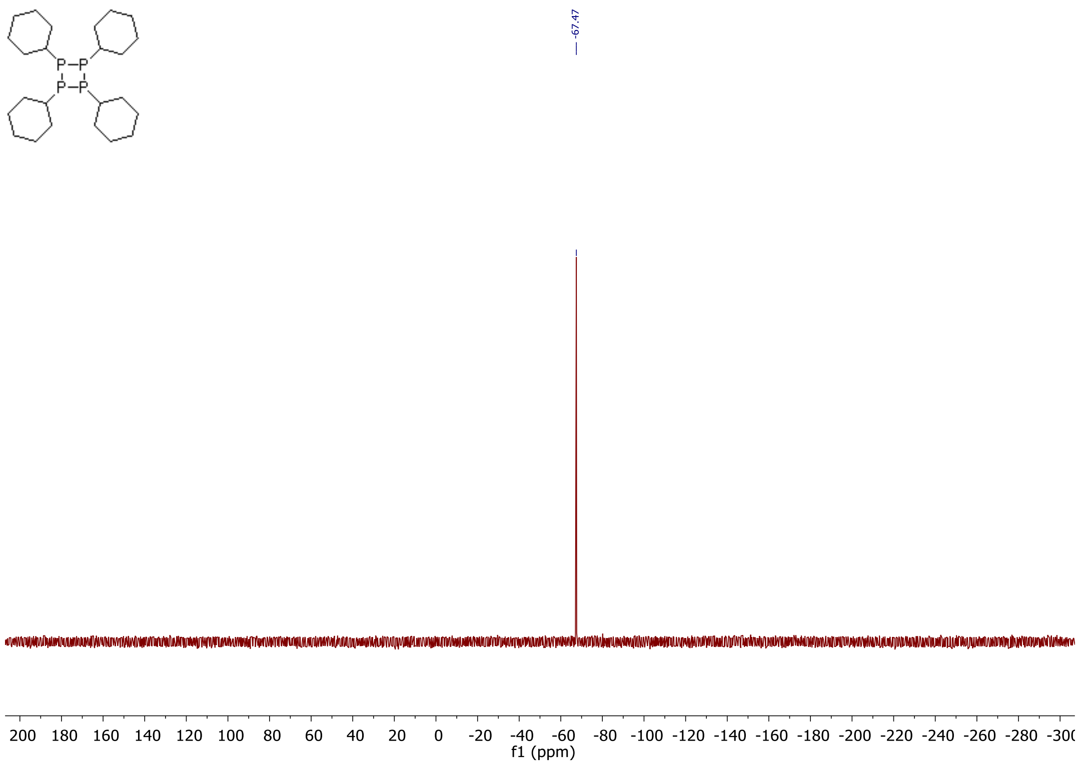

Supplementary Figure 46. ^{13}P{^{1}H} NMR spectrum of **2j** (Methylene Chloride-d_2)

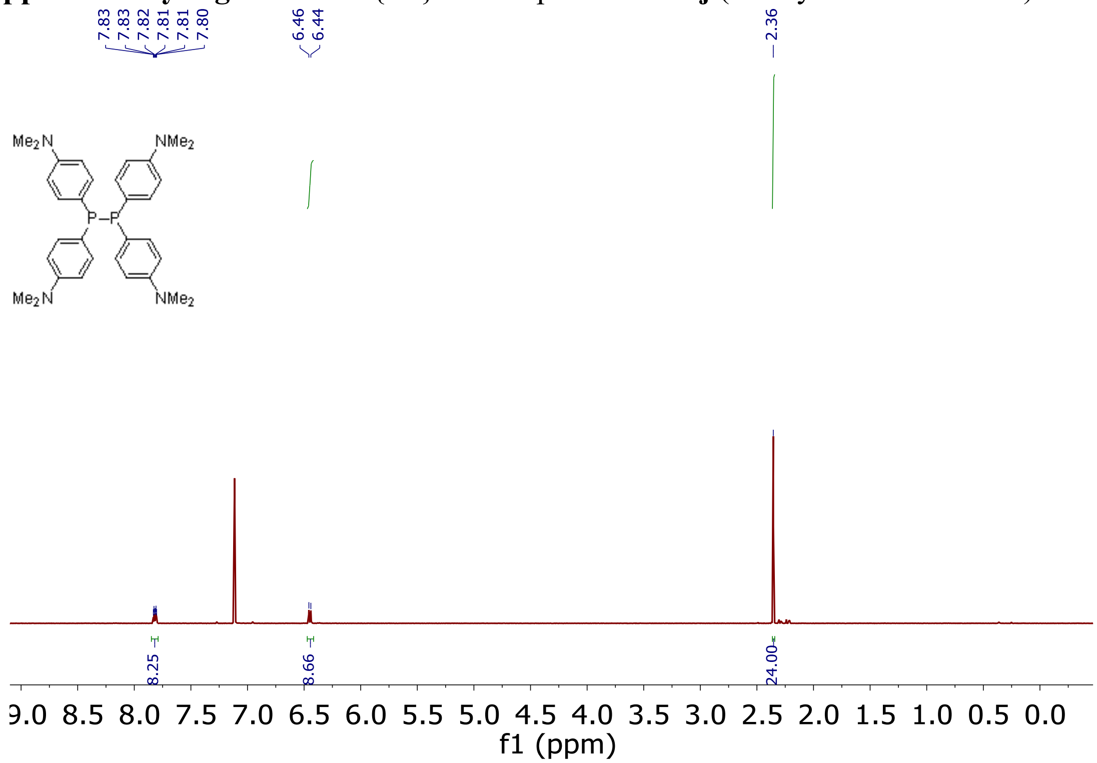

Supplementary Figure 47. ^{1}H NMR spectrum of **2l** (Benzene-d_6)

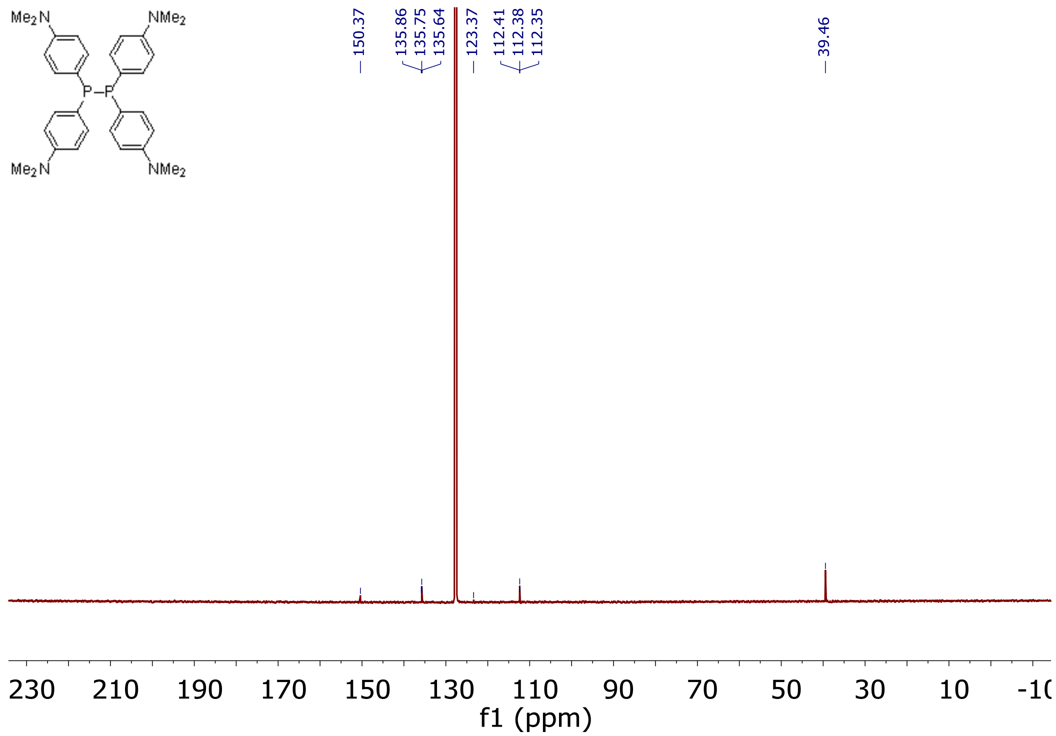

Supplementary Figure 48. ^{13}C NMR spectrum of **2l** (Benzene-d_6)

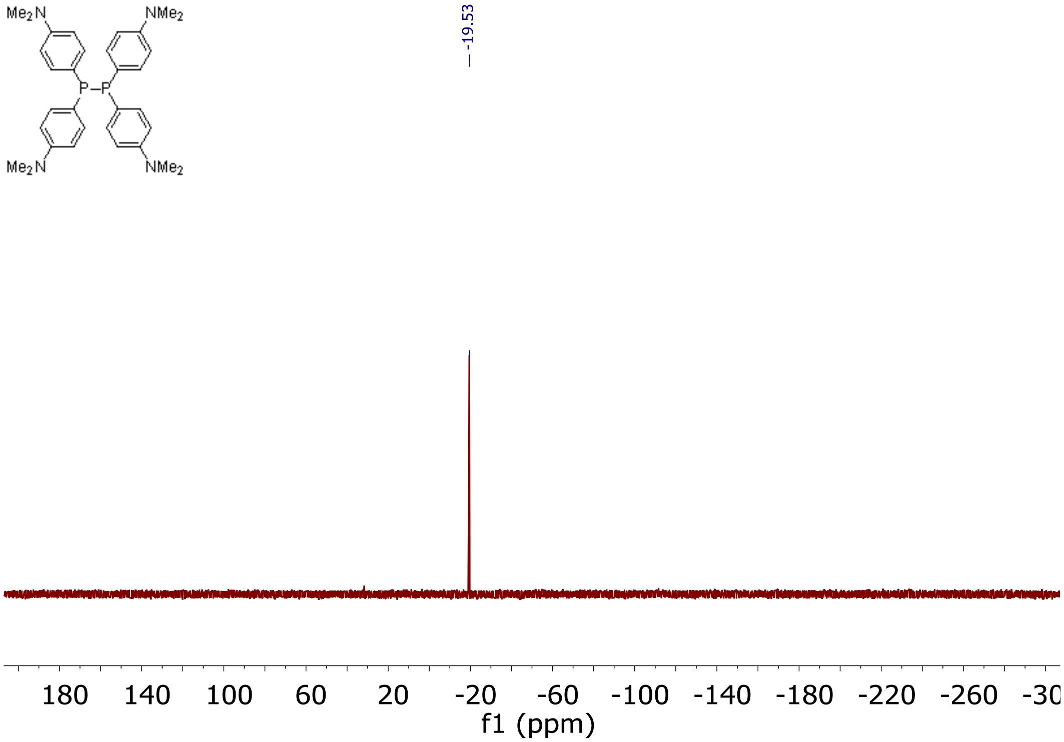

Supplementary Figure 49. ^{31}P NMR spectrum of **2l** (Benzene-d_6)

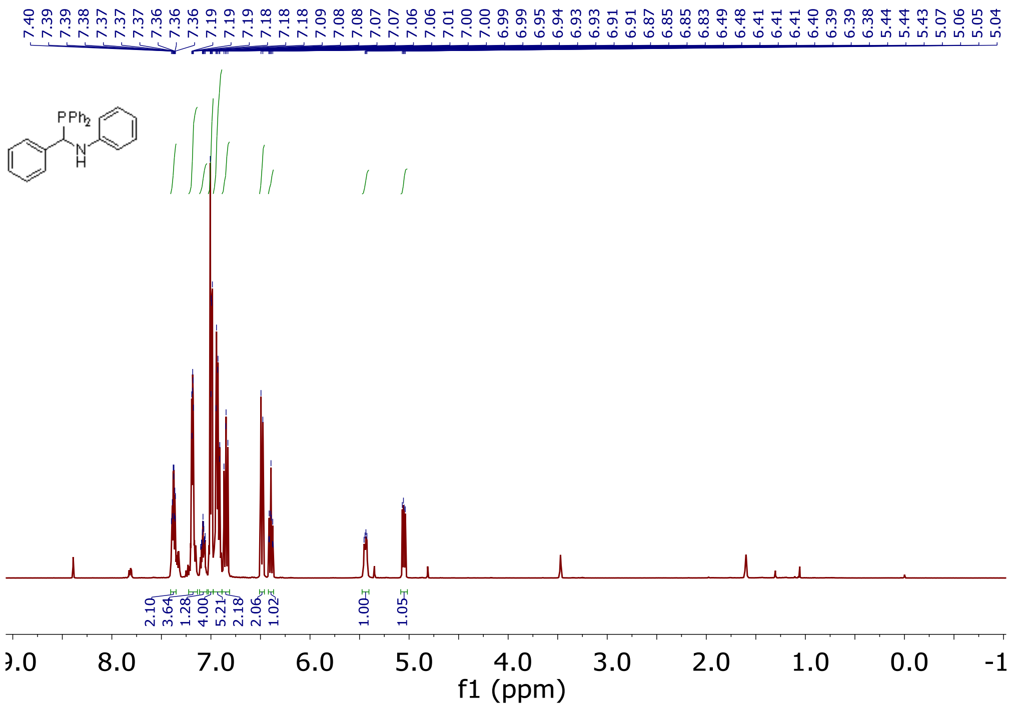

Supplementary Figure 50. ^{1}H NMR spectrum of **3a** (THF-d_8)

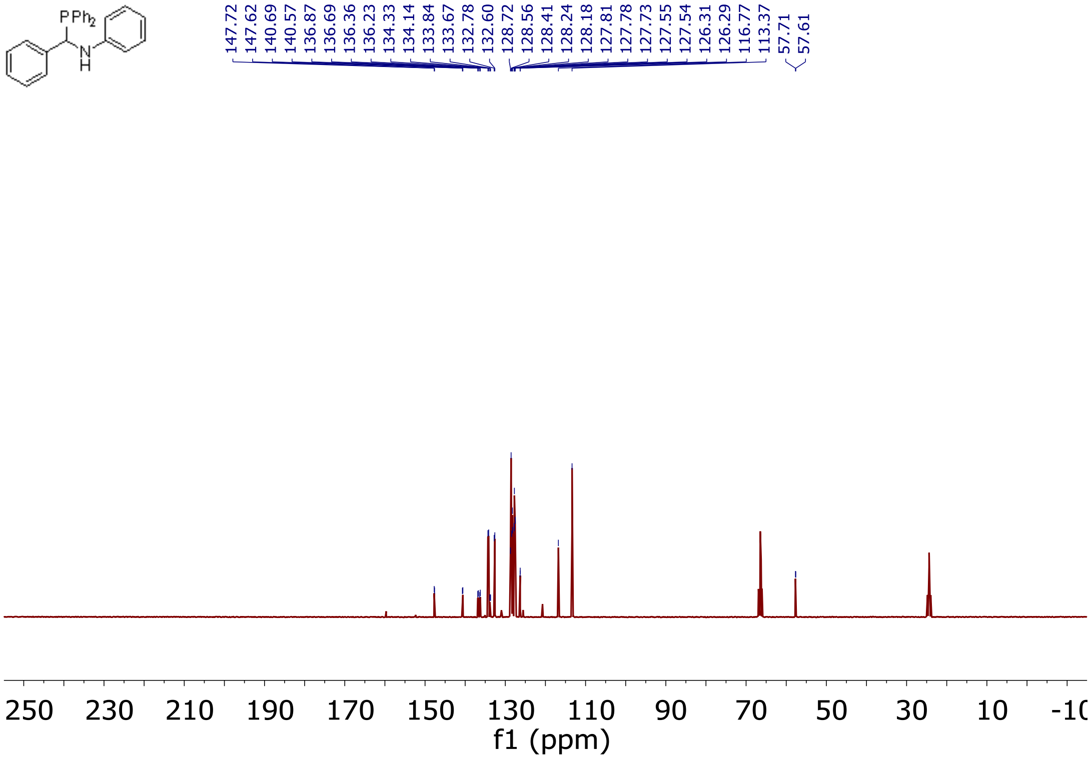

Supplementary Figure 51. ^{13}C NMR spectrum of **3a** (THF-d_8)

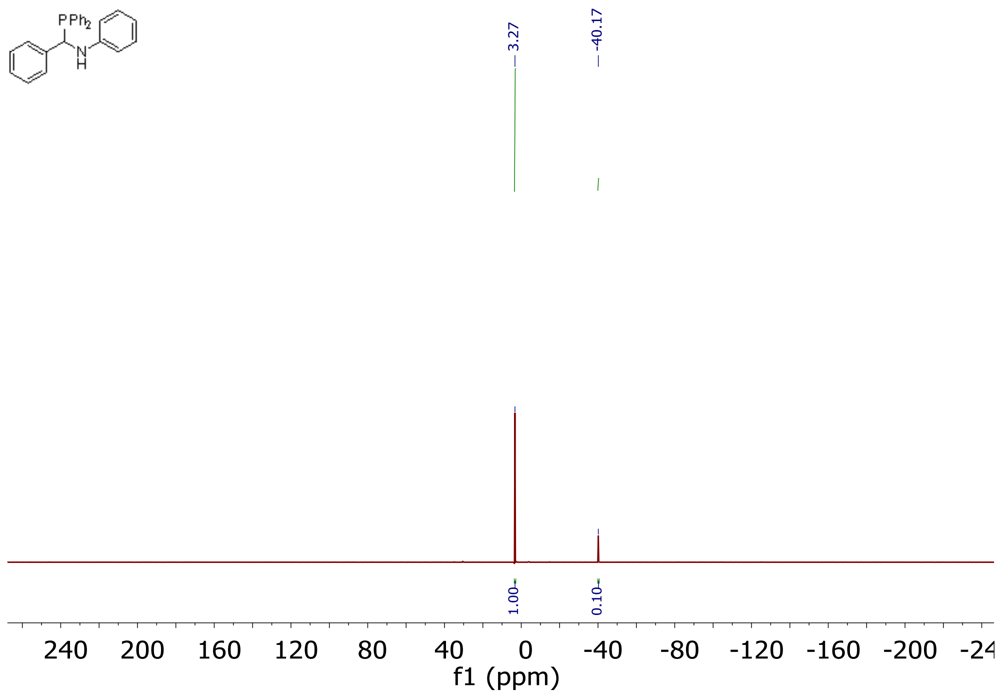

Supplementary Figure 52. $^{31}P\{^1H\}$ NMR spectrum of **3a** (THF-d_8)

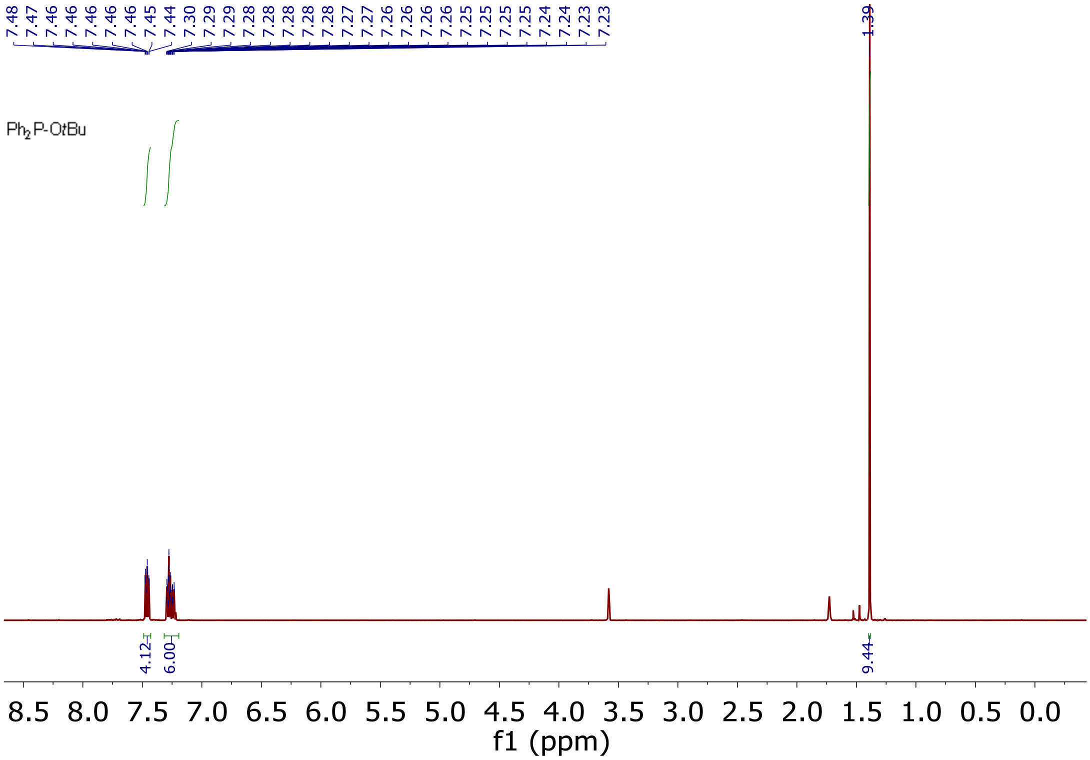

Supplementary Figure 53. ^1H NMR spectrum of **5a** (THF-d_8)

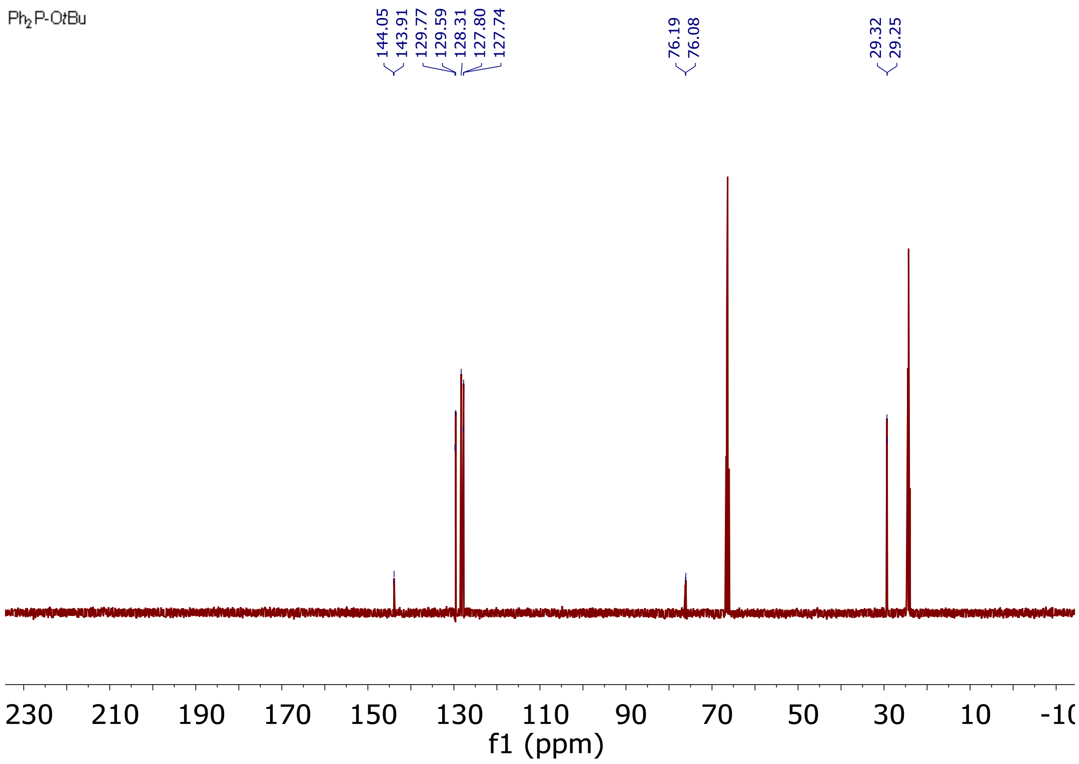

Supplementary Figure 54. ^{13}C NMR spectrum of **5a** (THF-d_8)

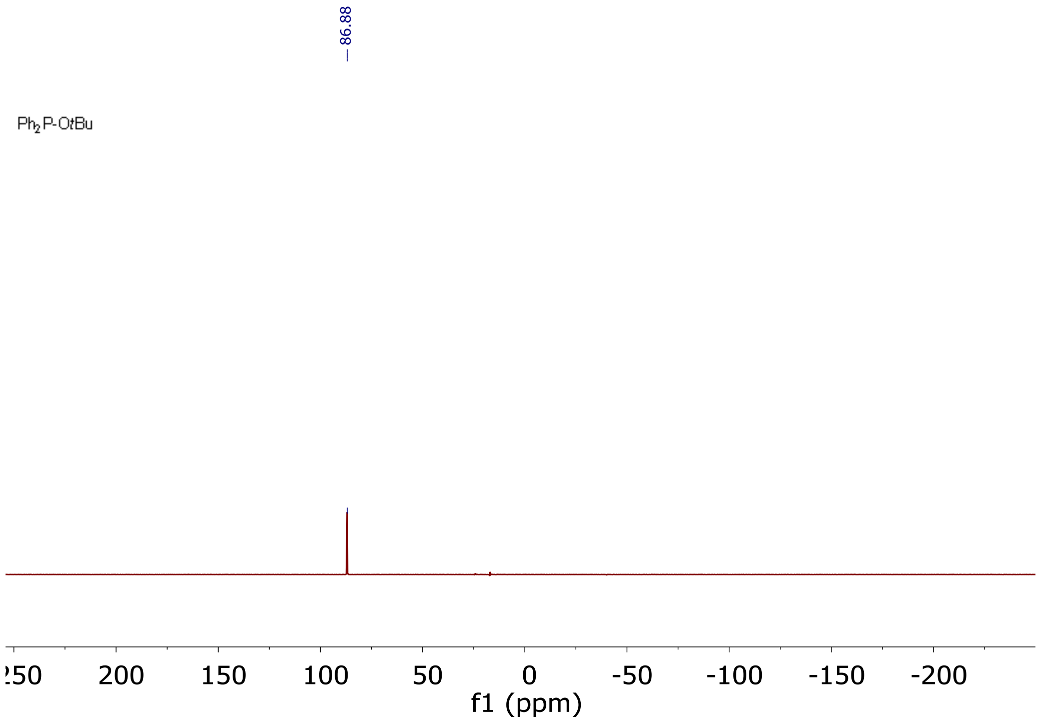

Supplementary Figure 55. ^{31}P NMR spectrum of **5a** (THF-d_8)

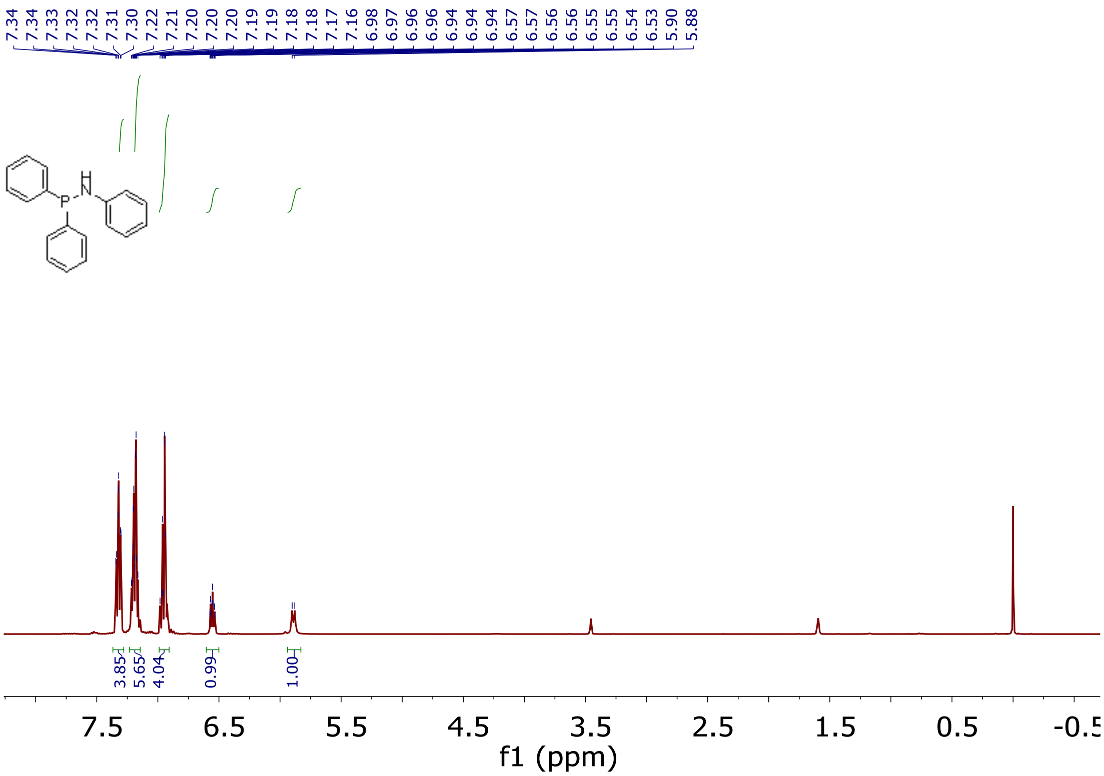

Supplementary Figure 56. ^{1}H NMR spectrum of **6a** (THF-d_8)

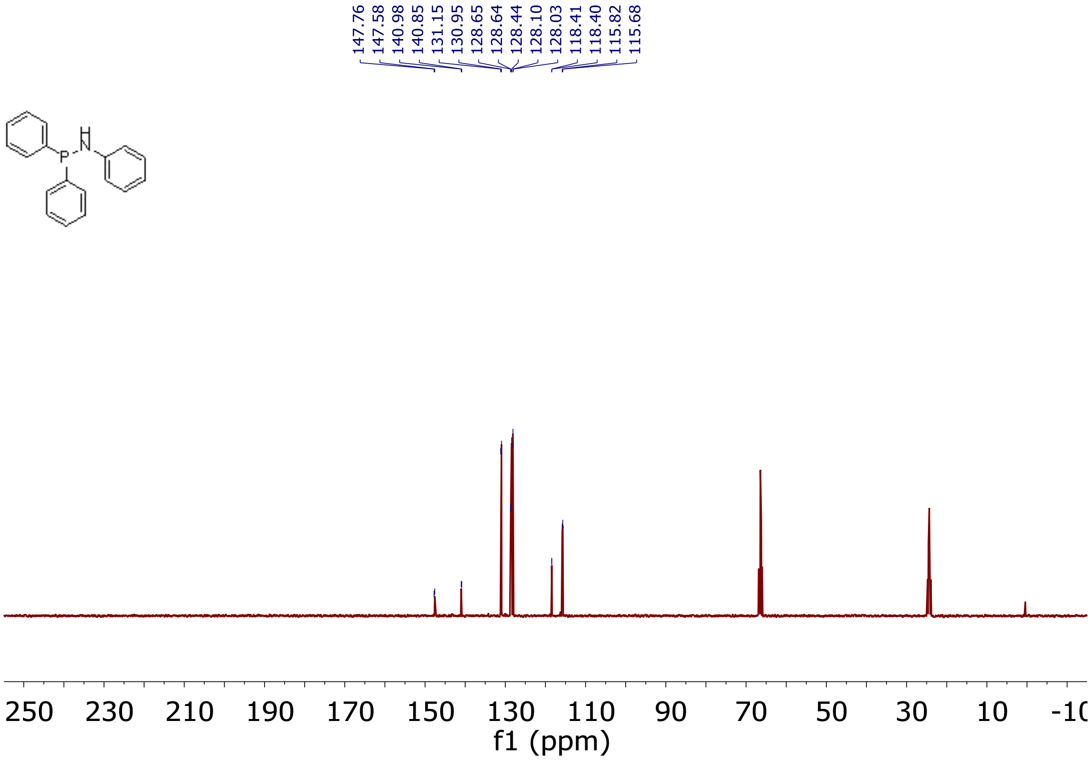

Supplementary Figure 57. ^{13}C NMR spectrum of **6a** (THF-d_8)

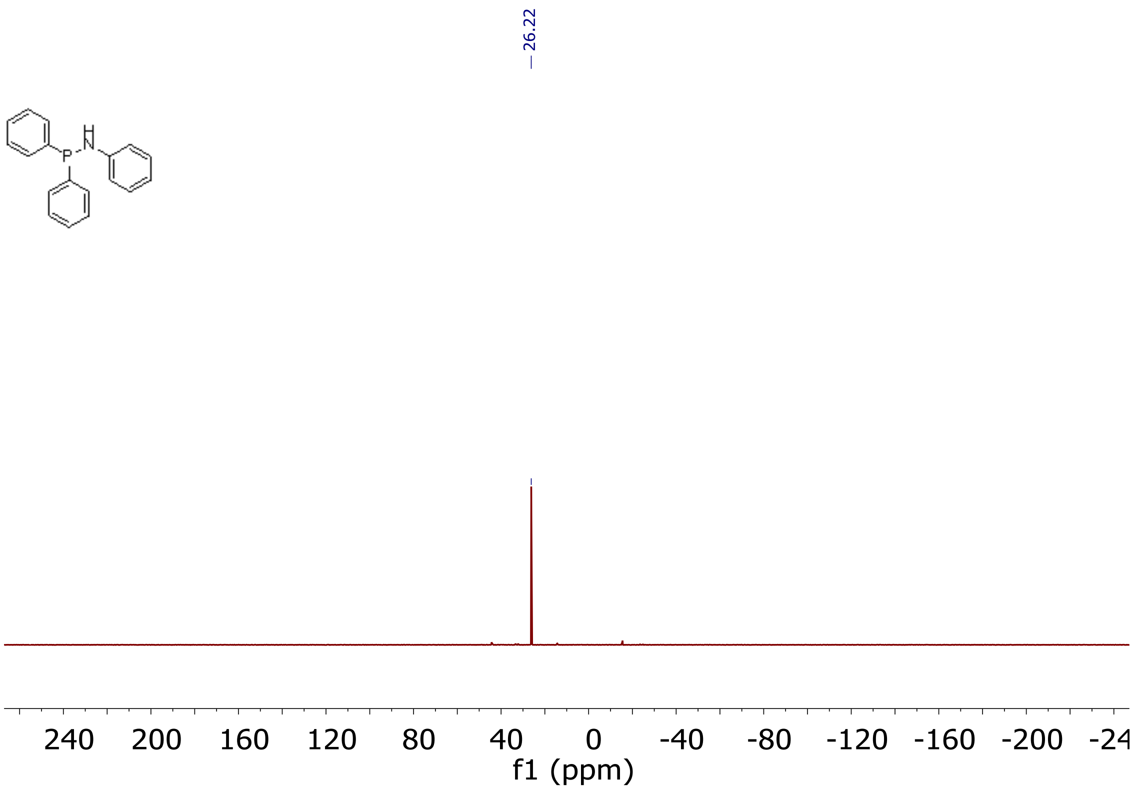

Supplementary Figure 58. ^{31}P NMR spectrum of **6a** (THF-d_8)

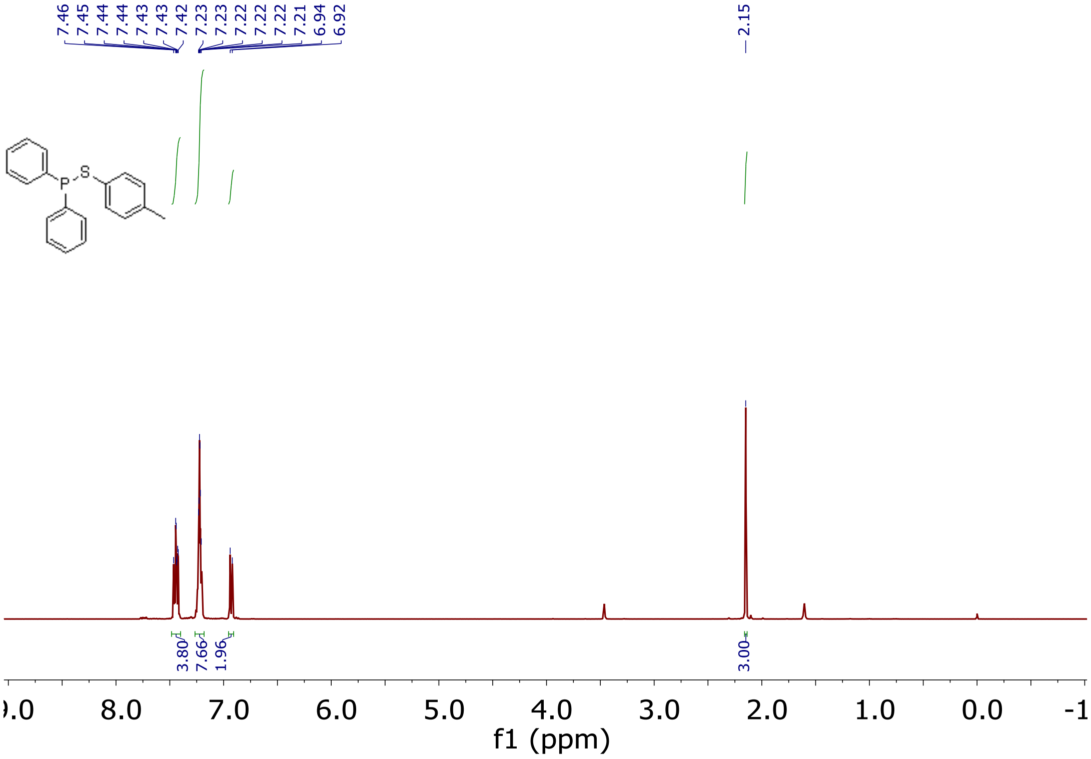

Supplementary Figure 59. ^{1}H NMR spectrum of **8a** (THF-d_8)

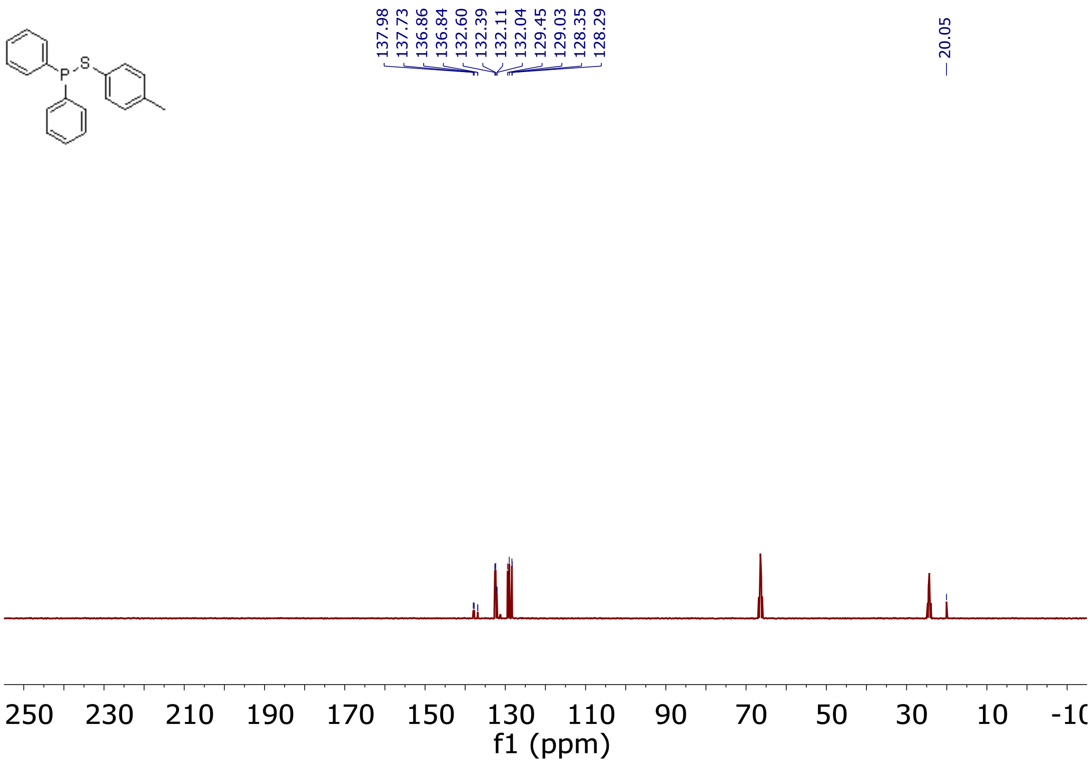

Supplementary Figure 60. ^{13}C NMR spectrum of **8a** (THF-d_8)

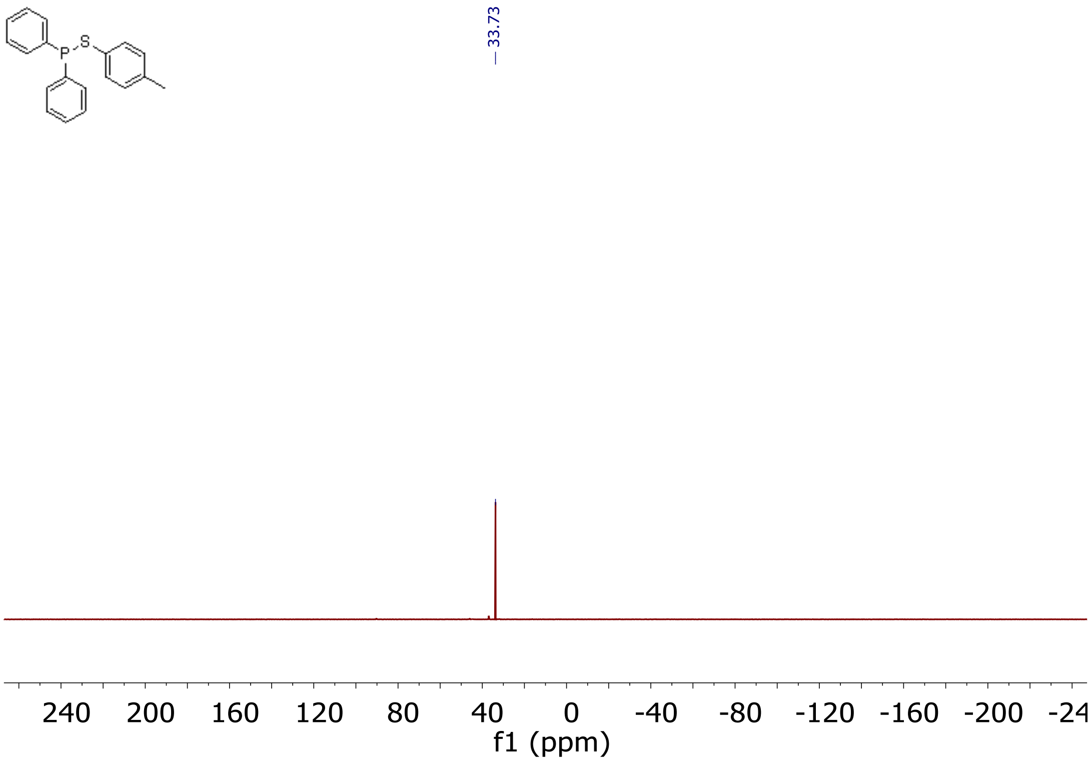

Supplementary Figure 61. ^{31}P NMR spectrum of **8a** (THF-d_8)

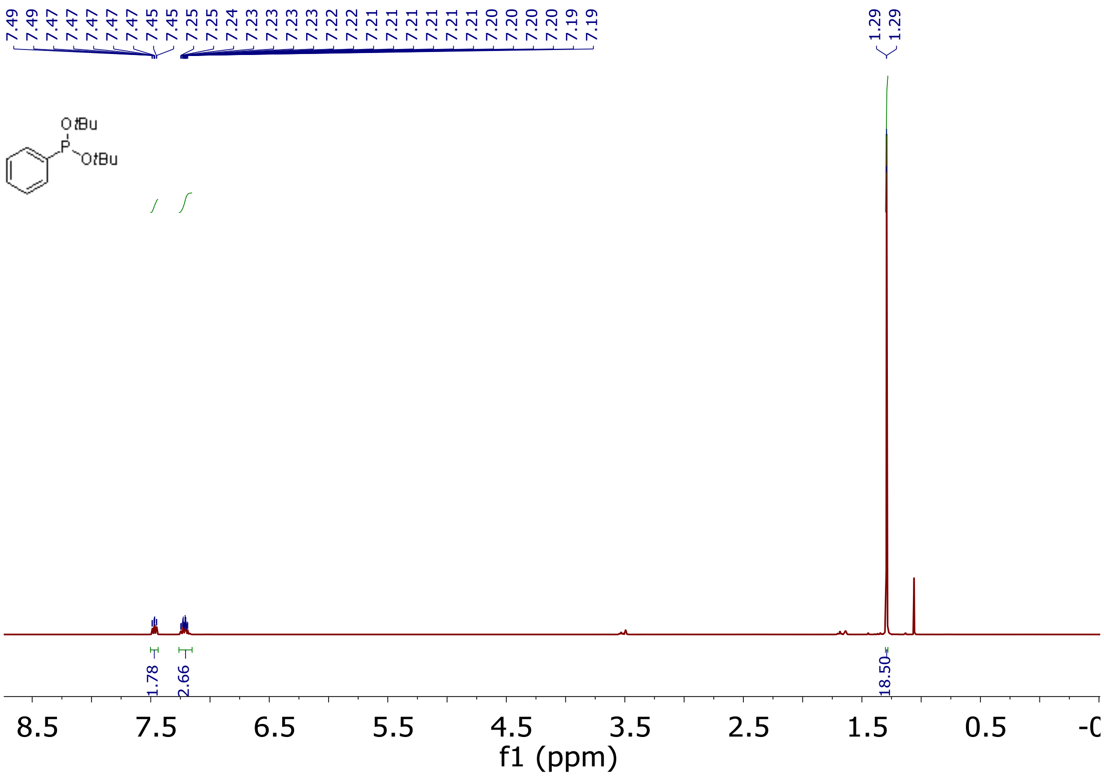

Supplementary Figure 62. ^{1}H NMR spectrum of **9a'** (THF-d_8)

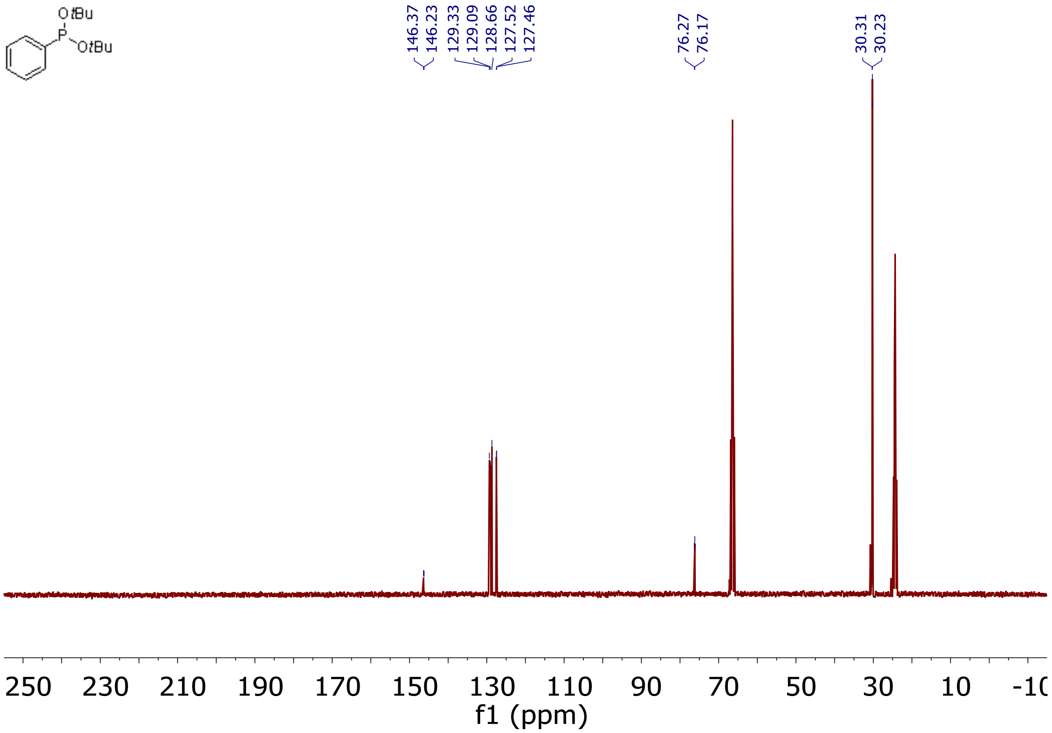

Supplementary Figure 63. ^{13}C NMR spectrum of **9a'** (THF-d_8)

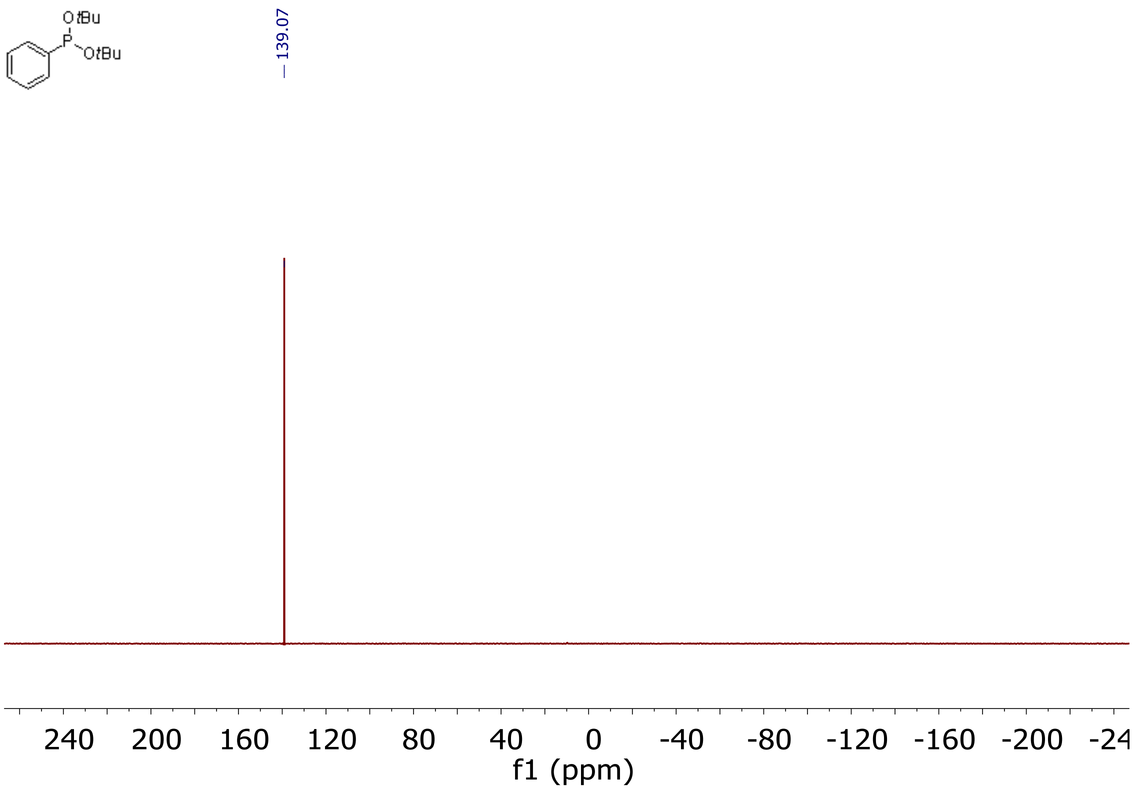

Supplementary Figure 64. ^{31}P NMR spectrum of **9a'** (THF-d_8)

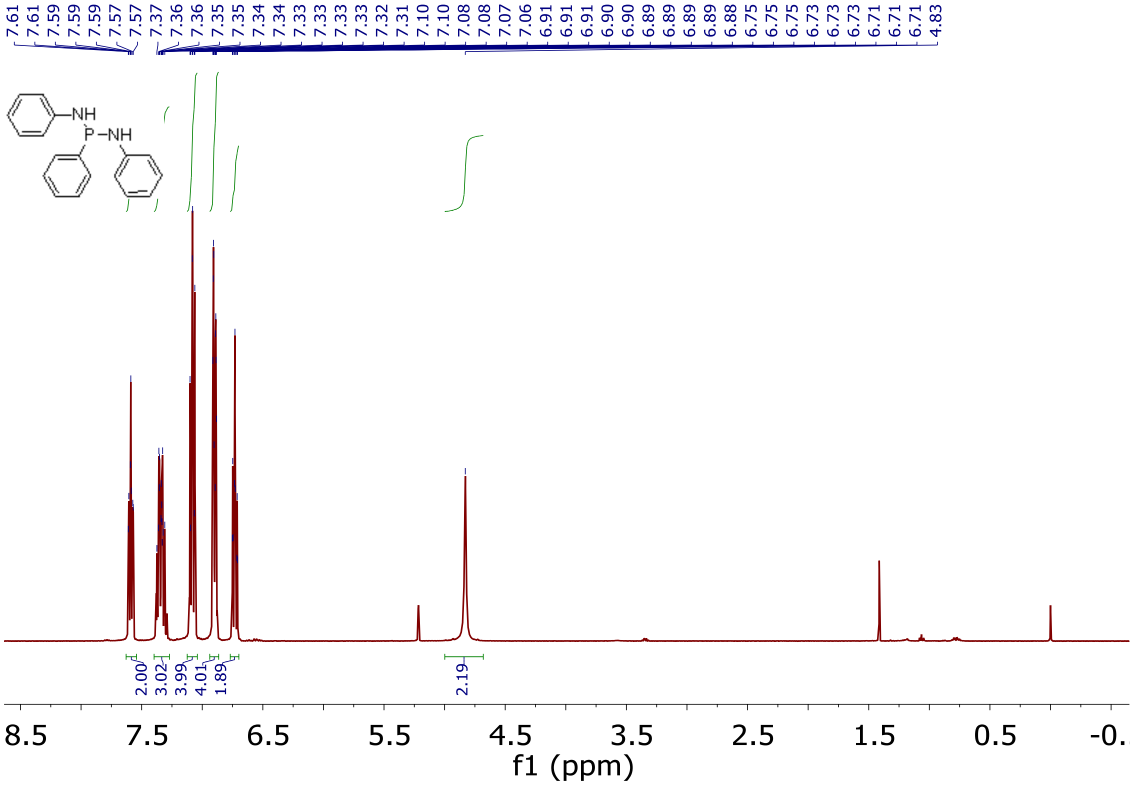

Supplementary Figure 65. ^{1}H NMR spectrum of **10a'** (THF-d_8)

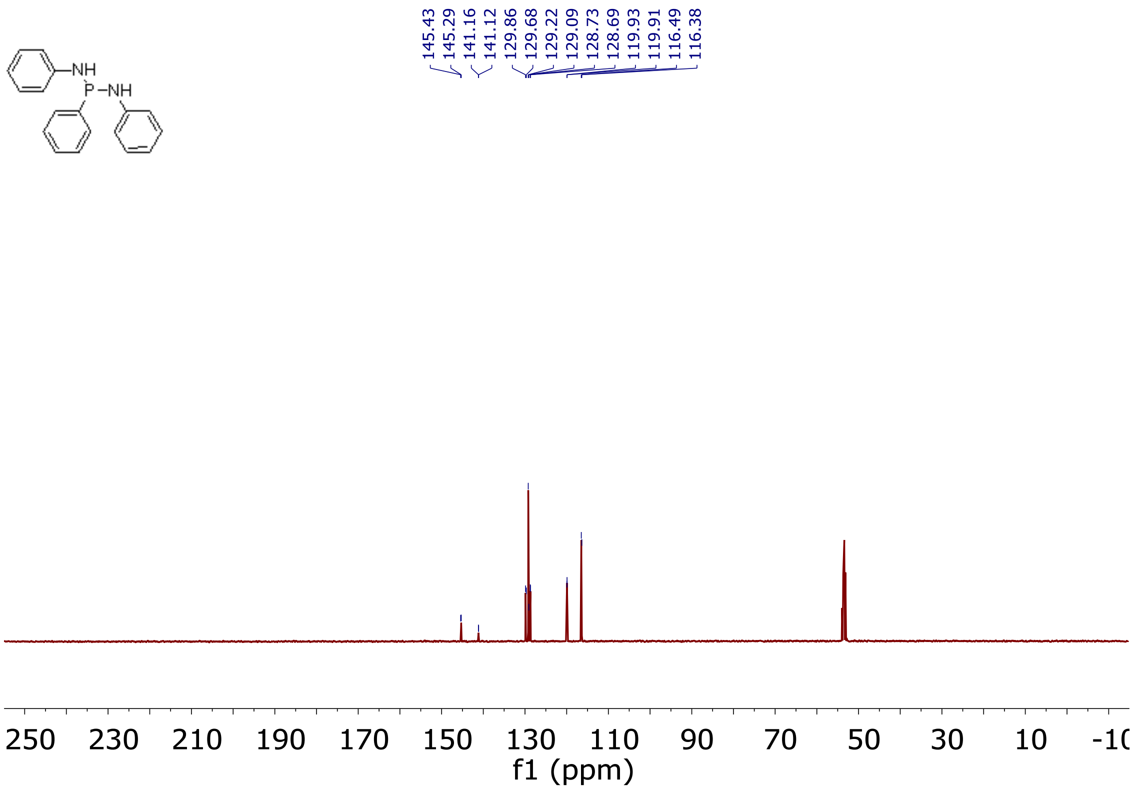

Supplementary Figure 66. ^{13}C NMR spectrum of **10a'** (THF-d_8)

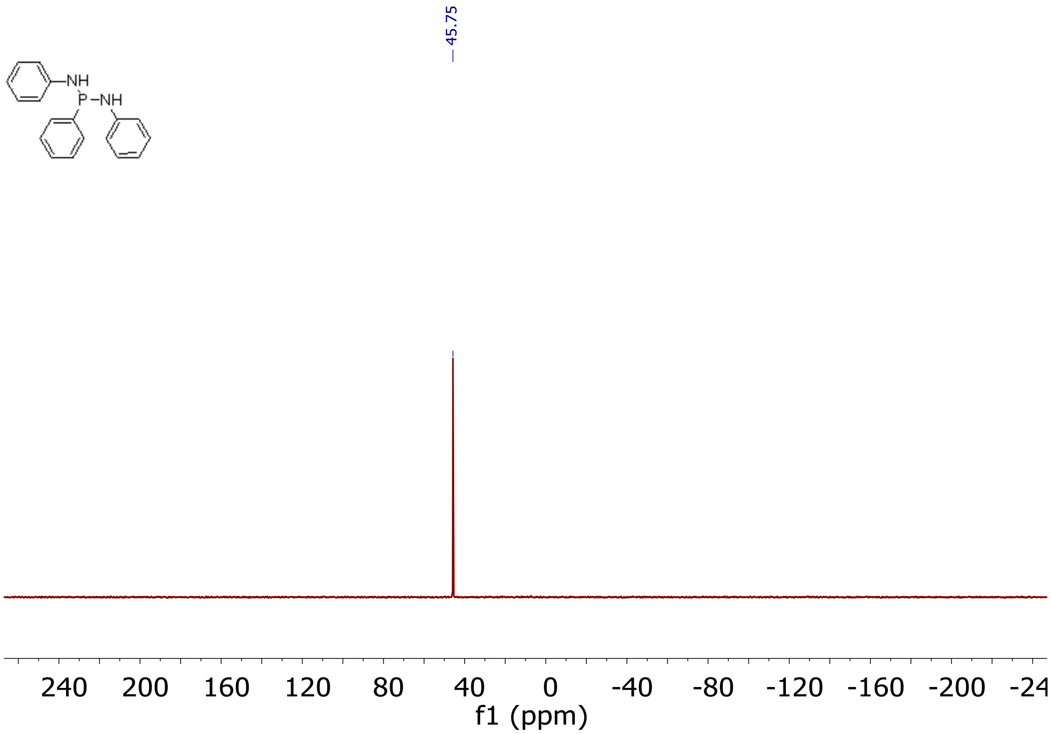

Supplementary Figure 67. ^{31}P NMR spectrum of **10a'** (THF-d_8)

Supplementary References

1 Taylor, R. C., Kolodny, R. & Walters, D. B. An Improved Method for the Preparation of Phenylphosphine and a Procedure for the Removal of the Toxic Vapors Produced. *Synth. React. Inorg. Met.-Org. Chem.* **3**, 175-179 (1973).

2 Molitor, S., Becker, J. & Gessner, V. H. Selective Dehydrocoupling of Phosphines by Lithium Chloride Carbenoids. *J. Am. Chem. Soc.* **136**, 15517-15520 (2014).

3 Bar-Nir Ben-Aroya, B. & Portnoy, M. Preparation of α-aminophosphines on solid support: model studies and parallel synthesis. *Tetrahedron* **58**, 5147-5158 (2002).

4 Fedotova, Y. V. *et al.* Phosphinohydrazines and phosphinohydrazides M(–N(R)–N(R)–PPh_2)$_n$ of some transition and main group metals: synthesis and characterization: Rearrangement of Ph_2P–NR–NR– ligands into aminoiminophosphorane, RNPPh_2–NR–, and related chemistry. *J. Organomet. Chem.* **689**, 3060-3074 (2004).

5 Buser, U., Ess, C. H. & Gerson, F. The radical anion of (E)-azobenzene revisited. *Magn. Reson. Chem.* **29**, 721-725 (1991).

6 Stoll, S. & Schweiger, A. EasySpin, a comprehensive software package for spectral simulation and analysis in EPR. *J. Magn. Reson.* **178**, 42-55 (2006).

7 Bruker-AXS Apex II software, Madison, WI, 2008.

8 Sheldrick, G. M. SADABS V2012/1, University of Gottingen, Germany.

9 Sheldrick, G. M. Acta Cryst. A64, 112 (2008).

10 Dolmanov, O. V.; Bourhis, L. J.; Gildea, R. J.; Howard, J. A. K.; Puschmann, H. J. Appl. Cryst., 42, 339 (2009).

11 Weigand, J. J., Burford, N., Davidson, R. J., Cameron, T. S. & Seelheim, P. New Synthetic Procedures to Catena-Phosphorus Cations: Preparation and Dissociation of the First cyclo-Phosphino-halophosphonium Salts. *J. Am. Chem. Soc.* **131**, 17943-17953 (2009).

12 Andrieu, J., Dietz, J., Poli, R. & Richard, P. Reversible P-C bond formation for saturated α-aminophosphine ligands in solution: stabilization by coordination to Cu(I). *New J. Chem.* **23**, 581-583 (1999).

13 Ashby, E. C. & Argyropoulos, J. N. Single electron transfer in the reaction of enolates with alkyl halides. *J. Org. Chem.* **50**, 3274-3283 (1985).

14 Gaussian 16, Revision B.01, Frisch, M. J.; Trucks, G. W.; Schlegel, H. B.; Scuseria, G. E.; Robb, M. A.; Cheeseman, J. R.; Scalmani, G.; Barone, V.; Petersson, G. A.; Nakatsuji, H.; Li, X.; Caricato, M.; Marenich, A. V.; Bloino, J.; Janesko, B. G.; Gomperts, R.; Mennucci, B.; Hratchian, H. P.; Ortiz, J. V.; Izmaylov, A. F.; Sonnenberg, J. L.; Williams-Young, D.; Ding, F.; Lipparini, F.; Egidi, F.; Goings, J.; Peng, B.; Petrone, A.; Henderson, T.; Ranasinghe, D.; Zakrzewski, V. G.; Gao, J.; Rega, N.; Zheng, G.; Liang, W.; Hada, M.; Ehara, M.; Toyota, K.; Fukuda, R.; Hasegawa, J.; Ishida, M.; Nakajima, T.; Honda, Y.; Kitao, O.; Nakai, H.; Vreven, T.; Throssell, K.; Montgomery, J. A., Jr.; Peralta, J. E.; Ogliaro, F.; Bearpark, M. J.; Heyd, J. J.; Brothers, E. N.; Kudin, K. N.; Staroverov, V. N.; Keith, T. A.; Kobayashi, R.; Normand, J.; Raghavachari, K.; Rendell, A. P.; Burant, J. C.; Iyengar, S. S.; Tomasi, J.; Cossi, M.; Millam, J. M.; Klene, M.; Adamo, C.; Cammi, R.; Ochterski, J. W.; Martin, R. L.; Morokuma, K.; Farkas, O.; Foresman, J. B.; Fox, D. J. Gaussian, Inc., Wallingford CT, 2016.

15 Zhao, Y. & Truhlar, D. G. The M06 suite of density functionals for main group thermochemistry, thermochemical kinetics, noncovalent interactions, excited states, and transition elements: two new functionals and systematic testing of four M06-class functionals and 12 other functionals. *Theor. Chem. Acc.* **120**, 215-241 (2008).

16 Schäfer, A., Huber, C. & Ahlrichs, R. Fully optimized contracted Gaussian basis sets of triple zeta valence quality for atoms Li to Kr. *J. Chem. Phys.* **100**, 5829-5835 (1994).

17 Wu, L., Chitnis, S. S., Jiao, H., Annibale, V. T. & Manners, I. Non-Metal-Catalyzed Heterodehydrocoupling of Phosphines and Hydrosilanes: Mechanistic Studies of $B(C_6F_5)_3$-Mediated Formation of P-Si Bonds. *J. Am. Chem. Soc.* **139**, 16780-16790 (2017).
