# Supplementary material for: Homo- and heterodehydrocoupling of phosphines mediated by alkali metal catalysts
Source: Nat Commun. 2019 Jun 26;10:2786. doi: 10.1038/s41467-019-09832-4 (PMC6594957; doi:10.1038/s41467-019-09832-4)
Supplement: Supplementary file 3 — Description of Additional Supplementary Files [file 41467_2019_9832_MOESM3_ESM.pdf]

Description of Additional Supplementary Information

File Name: Supplementary Data 1
Description: Computed energies for all molecules used at the M062X/TZVP level of density functional theory.

File Name: Supplementary Data 2
Description: Cartesian coordinates for used molecules at the M062X/TZVP level of density functional theory.
